# Author Correction: Association study in African-admixed populations across the Americas recapitulates asthma risk loci in non-African populations

**DOI:** 10.1038/s41467-019-12158-w

**Published:** 2019-09-04

**Authors:** Michelle Daya, Nicholas Rafaels, Tonya M. Brunetti, Sameer Chavan, Albert M. Levin, Aniket Shetty, Christopher R. Gignoux, Meher Preethi Boorgula, Genevieve Wojcik, Monica Campbell, Candelaria Vergara, Dara G. Torgerson, Victor E. Ortega, Ayo Doumatey, Henry Richard Johnston, Nathalie Acevedo, Maria Ilma Araujo, Pedro C. Avila, Gillian Belbin, Eugene Bleecker, Carlos Bustamante, Luis Caraballo, Alvaro Cruz, Georgia M. Dunston, Celeste Eng, Mezbah U. Faruque, Trevor S. Ferguson, Camila Figueiredo, Jean G. Ford, Weiniu Gan, Pierre-Antoine Gourraud, Nadia N. Hansel, Ryan D. Hernandez, Edwin Francisco Herrera-Paz, Silvia Jiménez, Eimear E. Kenny, Jennifer Knight-Madden, Rajesh Kumar, Leslie A. Lange, Ethan M. Lange, Antoine Lizee, Pissamai Maul, Trevor Maul, Alvaro Mayorga, Deborah Meyers, Dan L. Nicolae, Timothy D. O’Connor, Ricardo Riccio Oliveira, Christopher O. Olopade, Olufunmilayo Olopade, Zhaohui S. Qin, Charles Rotimi, Nicolas Vince, Harold Watson, Rainford J. Wilks, James G. Wilson, Steven Salzberg, Carole Ober, Esteban G. Burchard, L. Keoki Williams, Terri H. Beaty, Margaret A. Taub, Ingo Ruczinski, Rasika A. Mathias, Kathleen C. Barnes, CAAPA, Ayola Akim Adegnika, Ganiyu Arinola, Ulysse Ateba-Ngoa, Gerardo Ayestas, Hrafnhildur Bjarnadóttir, Adolfo Correa, Said Omar Leiva Erazo, Marilyn G. Foreman, Cassandra Foster, Li Gao, Jingjing Gao, Leslie Grammer, Mark Hansen, Tina Hartert, Yijuan Hu, Iain Königsberg, Kwang-Youn A. Kim, Pamela Landaverde-Torres, Javier Marrugo, Beatriz Martinez, Rosella Martinez, Luis F. Mayorga, Delmy-Aracely Mejia-Mejia, Catherine Meza, Solomon Musani, Shaila Musharoff, Oluwafemi Oluwole, Maria Pino-Yanes, Hector Ramos, Allan Saenz, Maureen Samms-Vaughan, Robert Schleimer, Alan F. Scott, Suyash S. Shringarpure, Wei Song, Zachary A. Szpiech, Raul Torres, Gloria Varela, Olga Marina Vasquez, Francisco M. De La Vega, Lorraine B. Ware, Maria Yazdanbakhsh

**Affiliations:** 1 Department of Medicine, University of Colorado Denver, Aurora, CO 80045 USA; 2 Department of Public Health Sciences, Henry Ford Health System, Detroit, MI 48202 USA; 3 Department of Genetics, Stanford University School of Medicine, Stanford, CA 94305 USA; 4 Department of Medicine, Johns Hopkins University, Baltimore, MD 21224 USA; 5 Department of Medicine, University of California San Francisco, San Francisco, CA 94143 USA; 6 Center for Human Genomics and Personalized Medicine, Wake Forest School of Medicine, Winston-Salem, 27157 USA; 7 Center for Research on Genomics & Global Health, National Institutes of Health, Bethesda, MD 20892 USA; 8 Department of Human Genetics, Emory University, Atlanta, GA 30322 USA; 9 Institute for Immunological Research, Universidad de Cartagena, Cartagena, 130000 Colombia; 10 Immunology Service, Universidade Federal da Bahia, Salvador, 401110170 Brazil; 11 Department of Medicine, Northwestern University, Chicago, IL 60611 USA; 12 Department of Genetics and Genomics, Icahn School of Medicine at Mount Sinai, New York, NY 10029 USA; 13 Department of Medicine, University of Arizona College of Medicine, Tucson, AZ 85724 USA; 14 Universidade Federal da Bahia, Salvador, 401110170 Brazil; 15 Department of Microbiology, Howard University College of Medicine, Washington, DC 20059 USA; 16 National Human Genome Center, Howard University College of Medicine, Washington, DC 20059 USA; 17 Caribbean Institute for Health Research, The University of the West Indies, Kingston, 00007 Jamaica; 18 Departamento de Biorregulacao, Universidade Federal da Bahia, Salvador, 401110170 Brazil; 19 Department of Medicine, Einstein Medical Center, Philadelphia, PA 19141 USA; 20 National Heart, Lung and Blood Institute, National Institutes of Health, Bethesda, MD 20892 USA; 21 Université de Nantes, INSERM, Centre de Recherche en Transplantation et Immunologie, UMR, 1064 ATIP-Avenir, Equipe 5, Nantes, France; 22 Department of Bioengineering and Therapeutic Sciences, University of California San Francisco, San Francisco, CA 94143 USA; 23 Facultad de Medicina, Universidad Católica de Honduras, San Pedro Sula, 21102 Honduras; 24 Universidad Tecnológica Centroamericana (UNITEC), Facultad de Ciencias Médicas, Tegucigalpa, Honduras; 25 Department of Pediatrics, Northwestern University, Chicago, IL 60611 USA; 26 Genetics and Epidemiology of Asthma in Barbados, The University of the West Indies, Chronic Disease Research Centre, Jemmots Lane, St. Michael, BB11115 Barbados; 27 Centro de Neumologia y Alergias, San Pedro Sula, 21102 Honduras; 28 Department of Medicine, University of Chicago, Chicago, IL 60637 USA; 29 Institute for Genome Sciences, University of Maryland School of Medicine, Baltimore, MD 21201 USA; 30 Laboratório de Patologia Experimental, Centro de Pesquisas Gonçalo Moniz, Salvador, 40296-710 Brazil; 31 Department of Medicine and Center for Global Health, University of Chicago, Chicago, IL 60637 USA; 32 Department of Biostatistics and Bioinformatics, Emory University, Atlanta, GA 30322 USA; 33 Faculty of Medical Sciences, The University of the West Indies, Queen Elizabeth Hospital, Bridgetown, St. Michael, BB11000 Barbados; 34 Department of Physiology and Biophysics, University of Mississippi Medical Center, Jackson, MS 39216 USA; 35 Departments of Biomedical Engineering and Biostatistics, Johns Hopkins University, Baltimore, MD 21205 USA; 36 Department of Human Genetics, University of Chicago, Chicago, IL 60637 USA; 37 Center for Individualized and Genomic Medicine Research, Henry Ford Health System, Detroit, MI 48202 USA; 38 Department of Epidemiology, Bloomberg School of Public Health, JHU, Baltimore, MD 21205 USA; 39 Department of Biostatistics, Bloomberg School of Public Health, JHU, Baltimore, MD 21205 USA; 40 Centre de Recherches Médicales de Lambaréné, BP:242, Lambaréné, 13901 Gabon; 41 Department of Chemical Pathology, University of Ibadan, Ibadan, 900001 Nigeria; 42 Faculty of Medicine, University of Iceland, 101 Reykjavík, Iceland; 43 Department of Medicine, University of Mississippi Medical Center, Jackson, MS 39216 USA; 44 Pulmonary and Critical Care Medicine, Morehouse School of Medicine, Atlanta, GA 30310 USA; 45 Data and Statistical Sciences, AbbVie, North Chicago, IL 60064 USA; 46 Illumina, Inc., San Diego, CA 92122 USA; 47 Department of Medicine, Vanderbilt University, Nashville, TN 37232 USA; 48 Department of Preventive Medicine, Northwestern University, Chicago, IL 60611 USA; 49 Instituto de Investigaciones Immunologicas, Universidad de Cartagena, Cartagena, 130000 Colombia; 50 Facultad de Ciencias de la Salud, Universidad Tecnológica Centroamericana (UNITEC), San Pedro Sula, 21102 Honduras; 51 Department of Child Health, The University of the West Indies, Kingston, 00007 Jamaica; 52 Department of Medicine, Johns Hopkins University, Baltimore, MD 21287 USA; 53 Biomedical Sciences Graduate Program, University of California San Francisco, San Francisco, CA 94158 USA; 54 Centro Medico de la Familia, San Pedro Sula, 21102 Honduras; 55 Department of Parasitology, Leiden University Medical Center, Leiden, 02333 Netherlands

**Keywords:** Genome-wide association studies, Genetic variation, Immunogenetics, Asthma, Genome-wide association studies, Genetic variation, Immunogenetics, Asthma

Correction to: *Nature Communications* 10.1038/s41467-019-08469-7, published online 20 February 2019.

The original version of this Article contained an error in the spelling of a member of the CAAPA Consortium, Hrafnhildur Bjarnadóttir which was incorrectly given as Hilda Bjarnadóttir. This has now been corrected in both the PDF and HTML versions of the Article.

