# Supplementary material for: Association study in African-admixed populations across the Americas recapitulates asthma risk loci in non-African populations
Source: Nat Commun. 2019 Feb 20;10:880. doi: 10.1038/s41467-019-08469-7 (PMC6382865; doi:10.1038/s41467-019-08469-7)
Supplement: Supplementary file 1 — Supplementary Information [file 41467_2019_8469_MOESM1_ESM.pdf]

Association study in African-admixed populations across the Americas recapitulates asthma risk loci in non-African populations

Michelle Daya, Nicholas Rafaels, Tonya M. Brunetti, Sameer Chavan, Albert M. Levin, Aniket Shetty, Christopher R. Gignoux, Meher Preethi Boorgula, Genevieve Wojcik, Monica Campbell, Candelaria Vergara, Dara G. Torgerson, Victor E. Ortega, Ayo Doumatey, Henry Richard Johnston, Nathalie Acevedo, Maria Ilma Araujo, Pedro C. Avila, Gillian Belbin, Eugene Bleecker, Carlos Bustamante, Luis Caraballo, Alvaro Cruz, Georgia M. Dunston, Celeste Eng, Mezbah U. Faruque, Trevor S. Ferguson, Camila Figueiredo, Jean G. Ford, Weiniu Gan, Pierre-Antoine Gourraud, Nadia N. Hansel, Ryan D. Hernandez, Edwin Francisco Herrera-Paz, Silvia Jiménez, Eimear E. Kenny, Jennifer Knight-Madden, Rajesh Kumar, Leslie A. Lange, Ethan M. Lange, Antoine Lizee, Pissamai Maul, Trevor Maul, Alvaro Mayorga, Deborah Meyers, Dan L. Nicolae, Timothy D. O'Connor, Ricardo Riccio Oliveira, Christopher O. Olopade, Olufunmilayo Olopade, Zhaohui S. Qin, Charles Rotimi, Nicolas Vince, Harold Watson, Rainford J. Wilks, James G. Wilson, Steven Salzberg, Carole Ober, Esteban G. Burchard, L. Keoki Williams, Terri H. Beaty, Margaret A. Taub, Ingo Ruczinski, Rasika Ann Mathias, and Kathleen C. Barnes on behalf of the Consortium on Asthma among African Populations of the Americas (CAAPA).

Supplementary Figures

Supplementary Figure 1: CAAPA principal components. This figure summarizes the first two principal components generated by GENESIS, using a combined dataset of 20,482 overlapping and linkage disequilibrium pruned SNPs, in 84 African (YRI) and 84 European (CEU) 1000 Genomes Project phase 3 subjects, 43 Native American (NAT) subjects and 13,775 CAAPA subjects genotyped on the African Diaspora Power Chip (ADPC) or Multi-Ethnic Genotyping Array (MEGA).

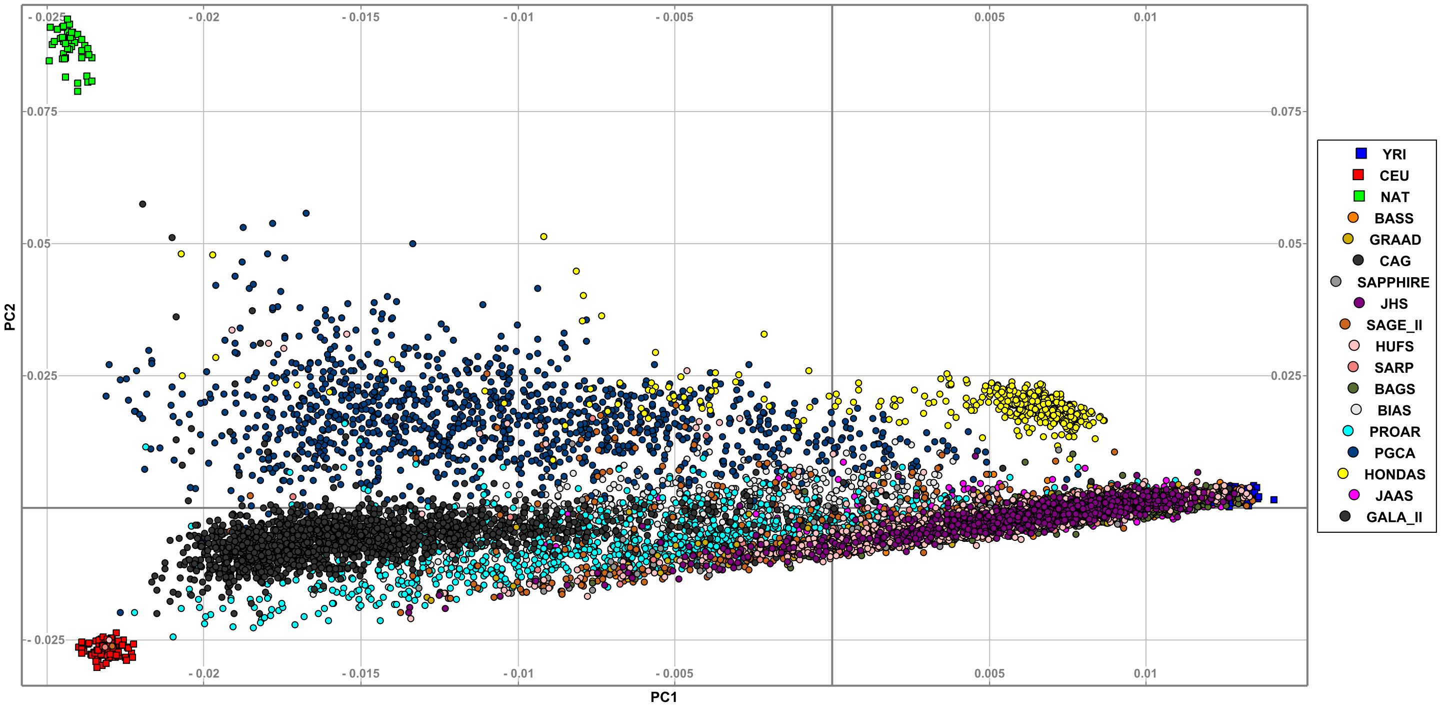

Supplementary Figure 2: Scree plots of the within-dataset principal component analysis. Principal components were calculated using GENESIS separately for each dataset, excluding reference population panels. The plots show the eigenvalues of each principal component. Principal component 1 explains most of the variance in the data for all the CAAPA datasets.

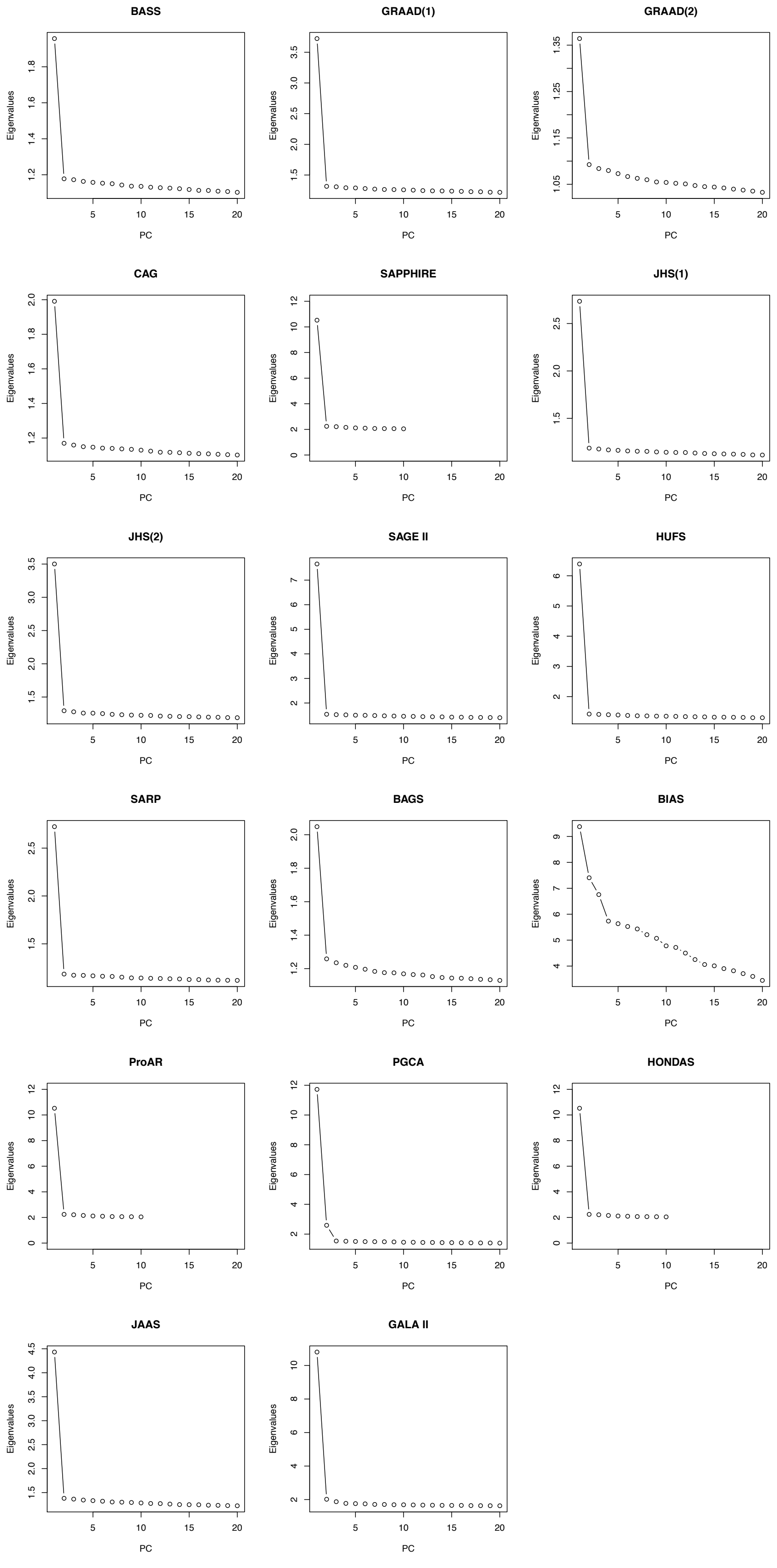

Supplementary Figure 3: QQ plots of genome-wide SNP associations with asthma for common SNPs (SNPs with MAF ≥ 0.05) by CAAPA data set. Inflation factors were calculated by transforming p-values to 1 degree of freedom (df) Chi-square statistics, and dividing the median of these statistics by the median of the theoretical Chi-square (1 df) distribution.

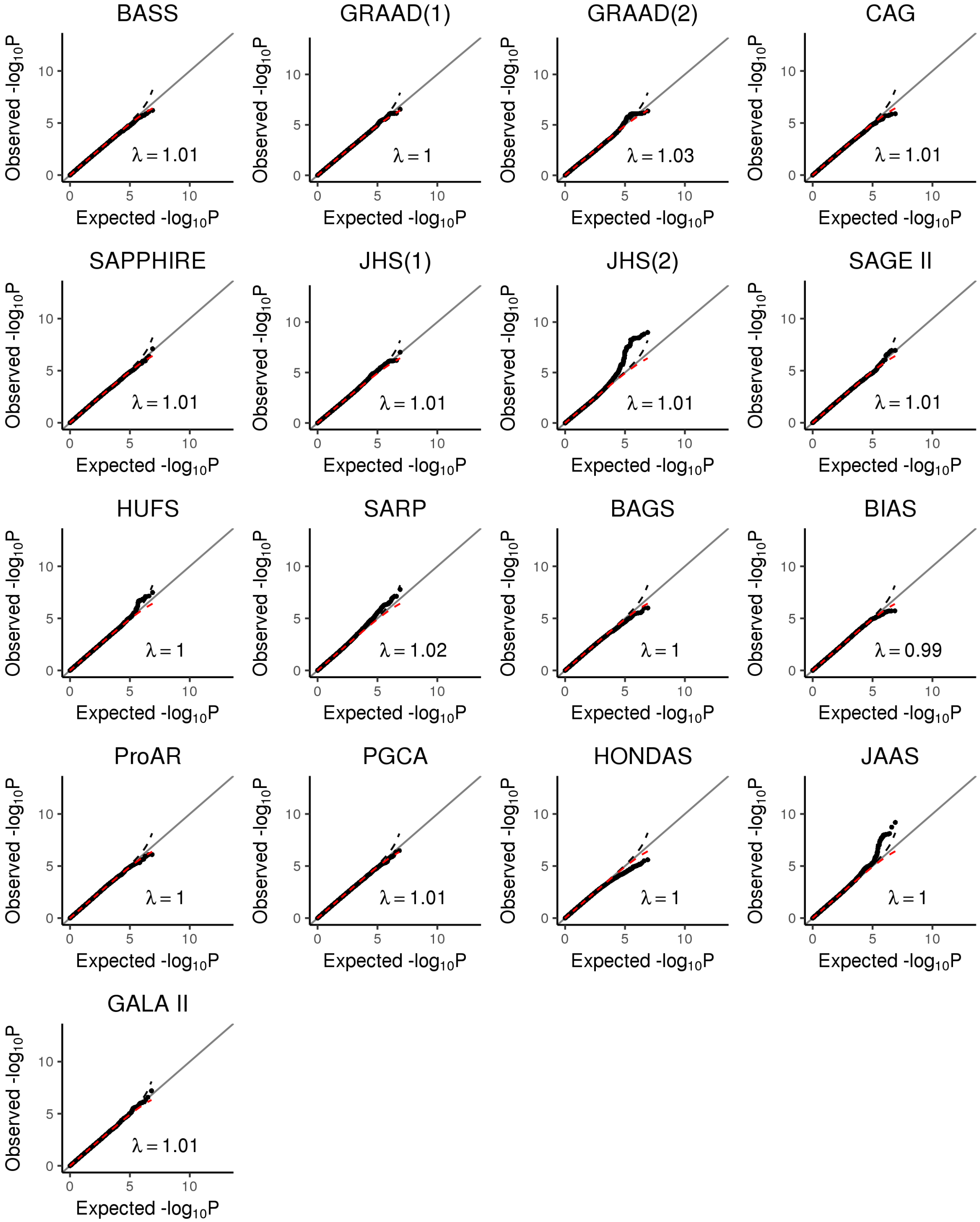

Supplementary Figure 4: QQ plots of genome-wide SNP associations with asthma for rare SNPs (SNPs with MAF < 0.05) by CAAPA data set. SNPs with minor allele count ≤ 10 were filtered out from the result sets. Inflation factors were calculated by transforming p-values to 1 degree of freedom (df) Chi-square statistics, and dividing the median of these statistics by the median of the theoretical Chi-square (1 df) distribution.

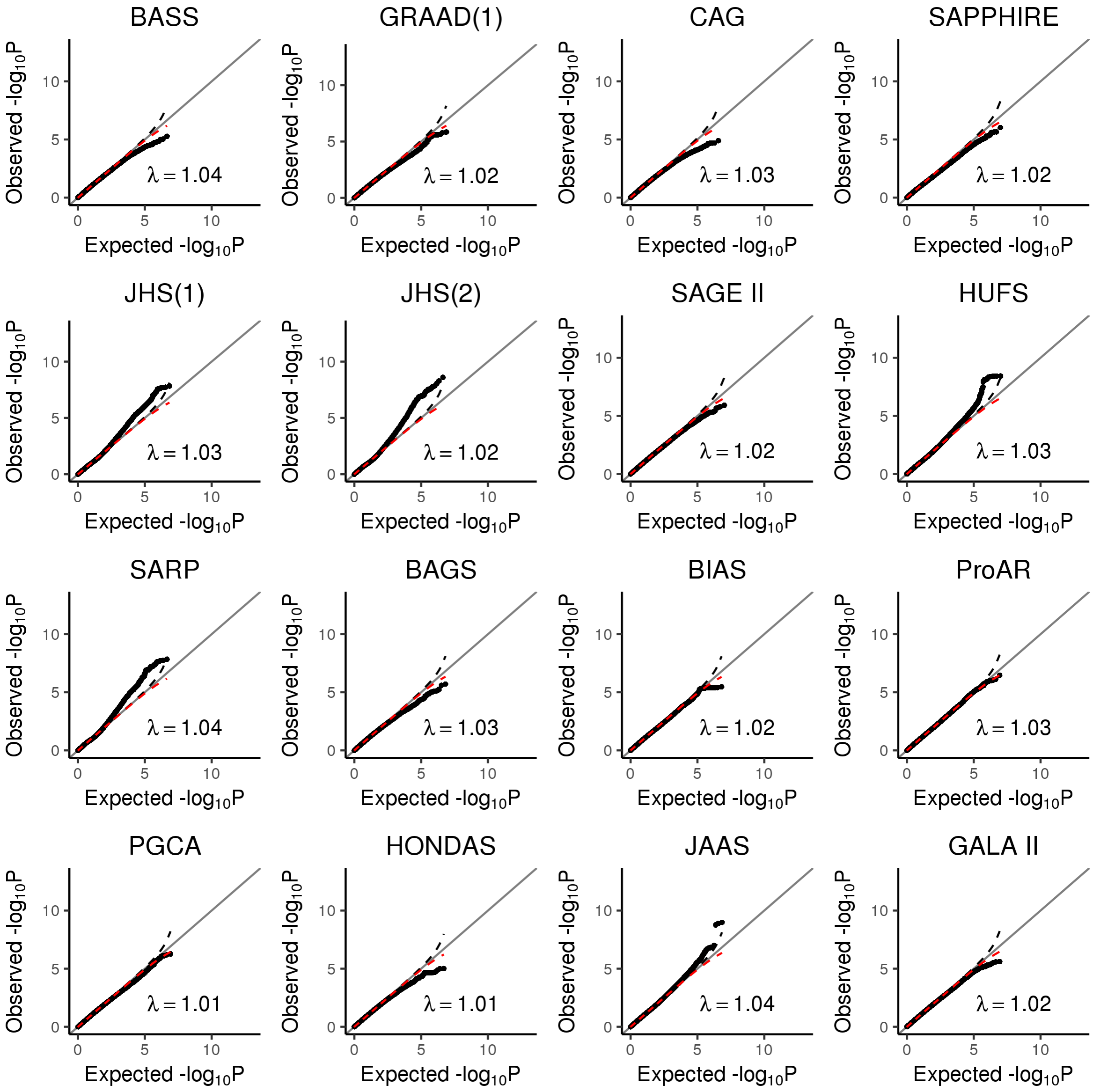

Supplementary Figure 5. **Genetic axes of variation inferred by MR-MEGA for the 17 CAAPA data sets included in the meta-analysis.** The x and y axes represent the first and second multi-dimensional scaling (MDS) components, respectively. The position of each data set on these genetic axes is plotted, colored by the mean proportion of African (YRI), European (CEU) and Native American (NAT) ancestry in the data set (estimated by ADMIXTURE), in the three panels, respectively.

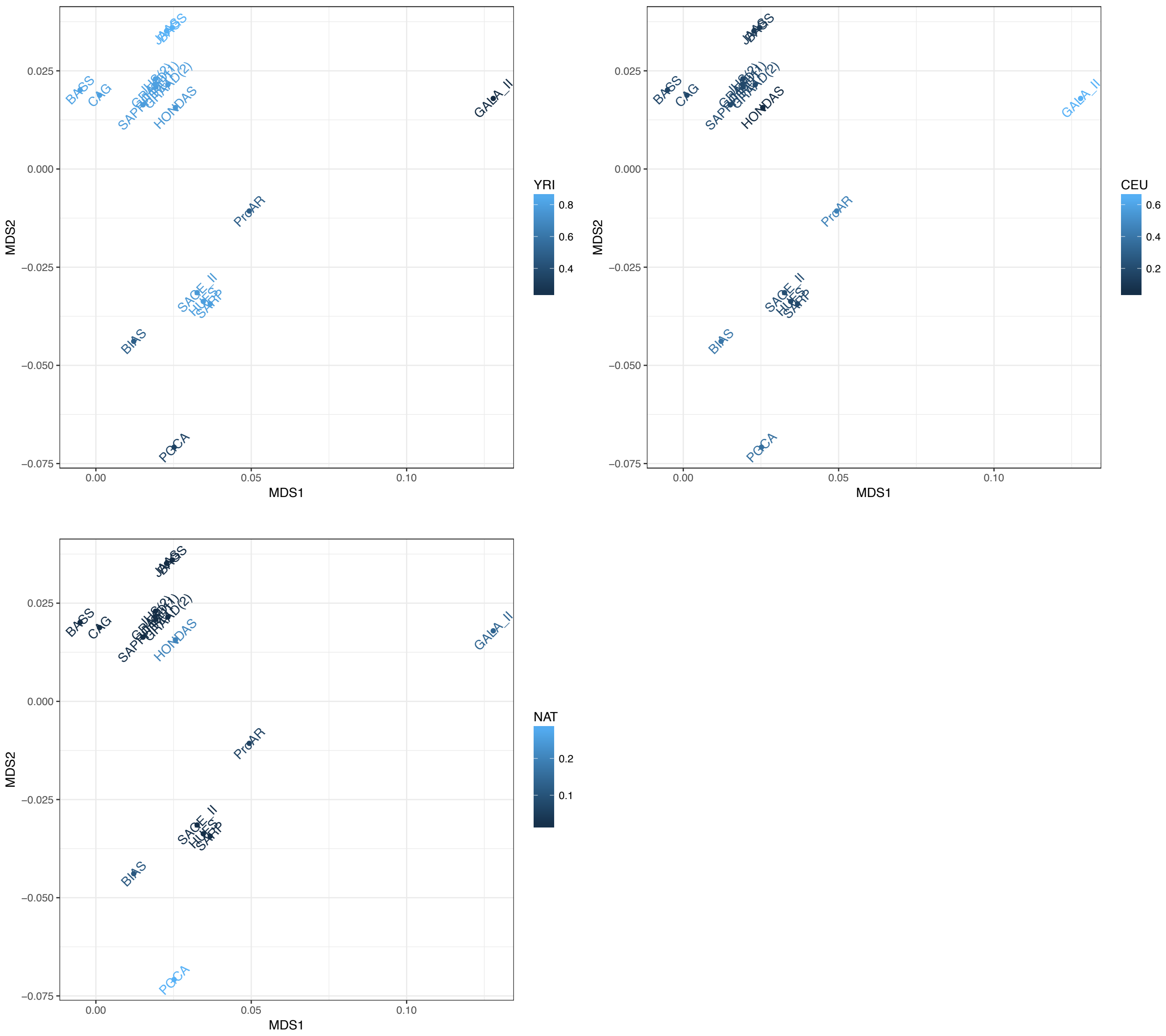

Supplementary Figure 6: Locus zoom plot of associations in the chromosome 6 admixture mapping peak. The p-values were estimated by inverse-variance meta-analysis of the SNP associations of studies included in the admixture mapping discovery dataset. The r^2 between the SNP colored in purple and other SNPs in the data set were estimated using African American subjects in the CAAPA whole genome sequence reference panel.

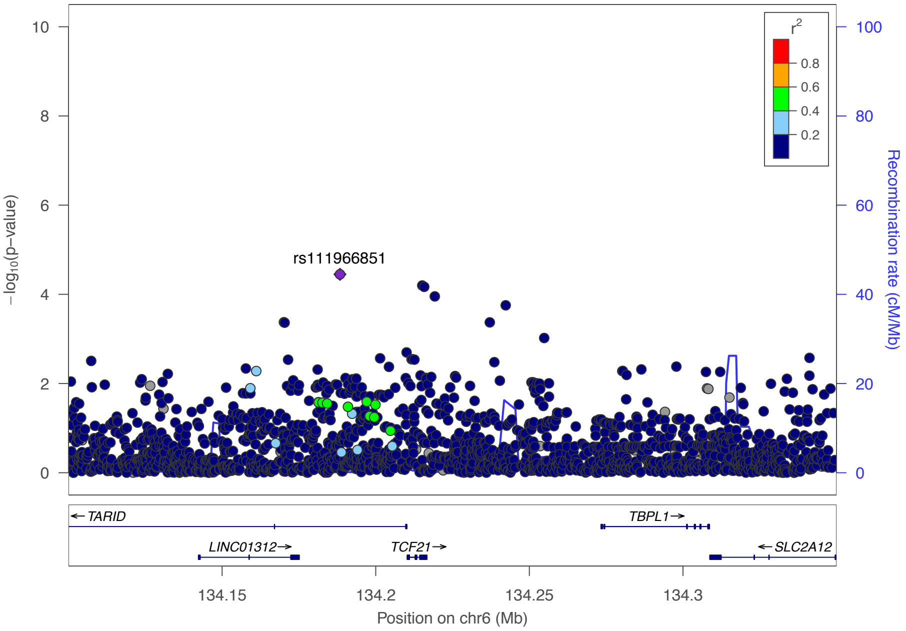

Supplementary Figure 7: Locus zoom plot of the chromosome 8p23 region identified by the CAAPA MR-MEGA meta-analysis. The r^2 between the SNP colored in purple and other SNPs in the data set were estimated using African American subjects in the CAAPA whole genome sequence reference panel.

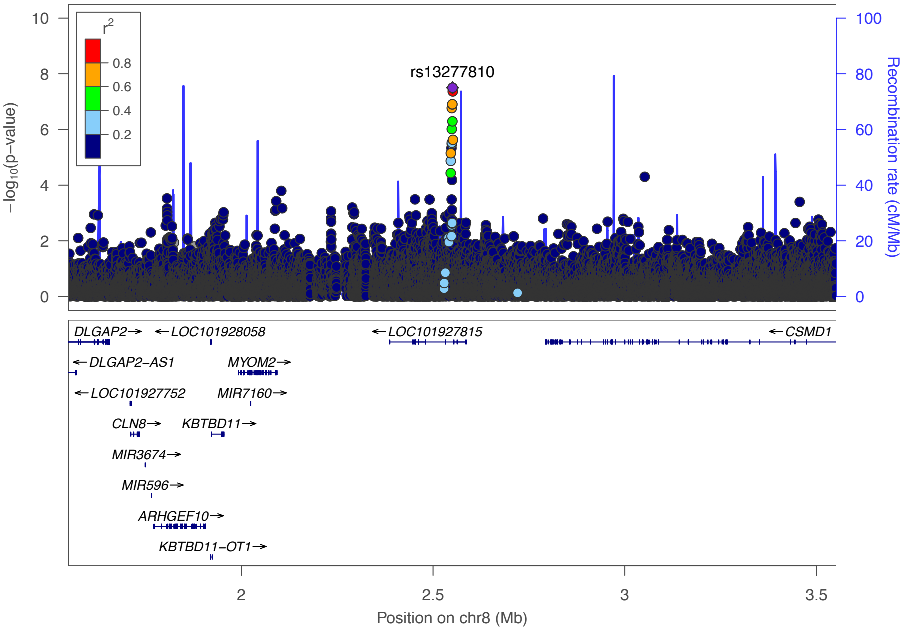

Supplementary Figure 8: Locus zoom plot of the chromosome 8q24 region identified by the CAAPA MR-MEGA meta-analysis. The ${}^2r$ between the SNP colored in purple and other SNPs in the data set were estimated using African American subjects in the CAAPA whole genome sequence reference panel.

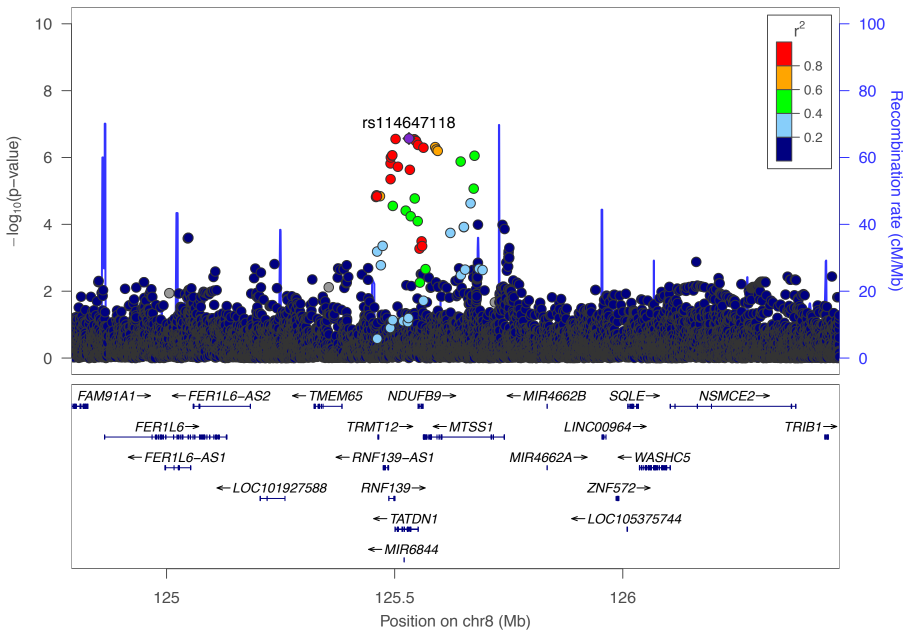

Supplementary Figure 9: Locus zoom plot of the chromosome 12q13 region identified by the CAAPA MR-MEGA meta-analysis. The r^2 between the SNP colored in purple and other SNPs in the data set were estimated using African American subjects in the CAAPA whole genome sequence reference panel.

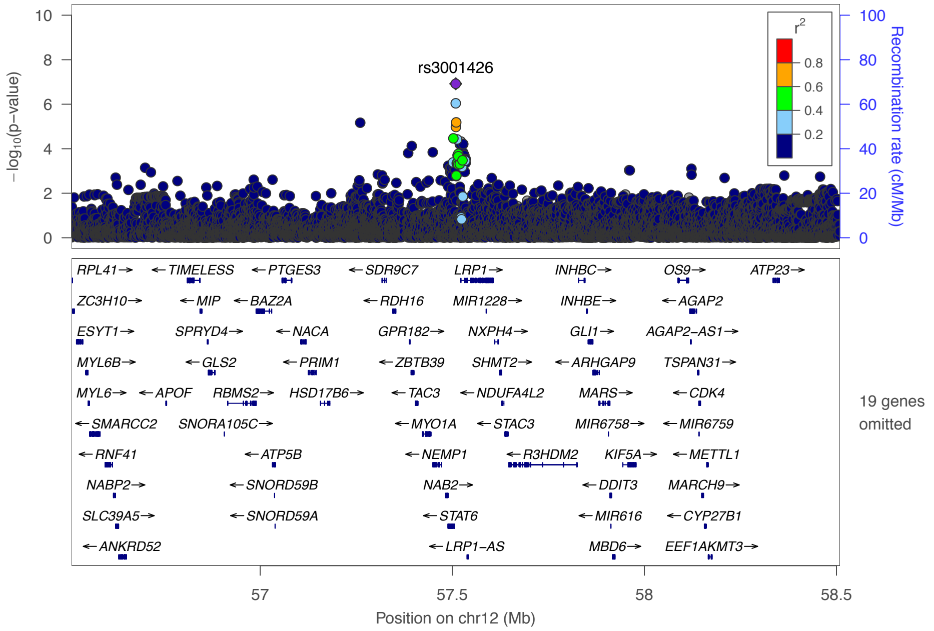

Supplementary Figure 10: Locus zoom plot of the chromosome 15q22 region identified by the CAAPA MR-MEGA meta-analysis. The r^2 between the SNP colored in purple and other SNPs in the data set were estimated using African American subjects in the CAAPA whole genome sequence reference panel.

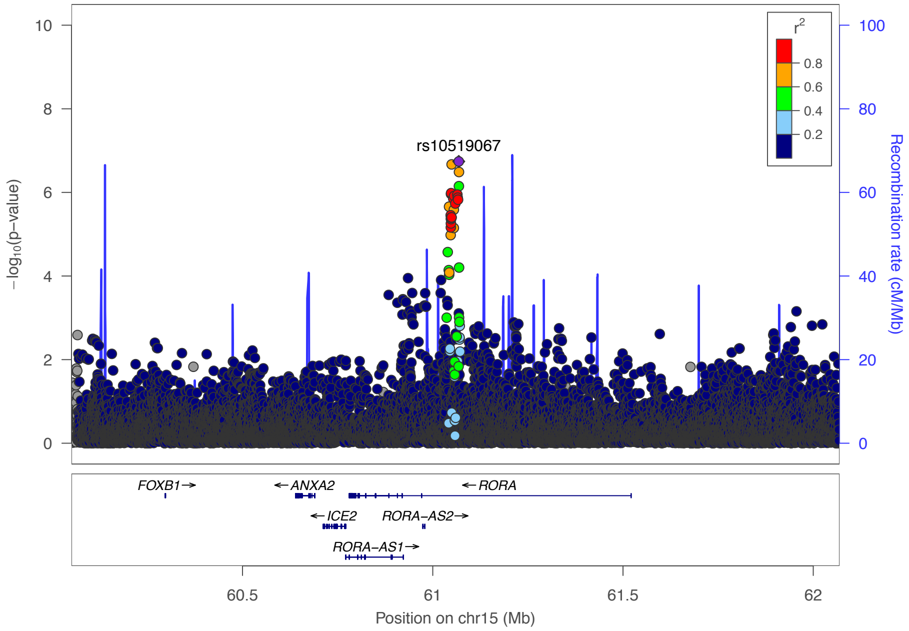

Supplementary Figure 11: Locus zoom plot of the chromosome 17q21 in studies with high African ancestry. Association statistics were generated by means of inverse-variance meta-analysis of CAAPA studies with mean African ancestry percentage > 75% (BASS, CAG, SAPPHIRE, HONDAS, JHS, JAAS, GRAAD, BAGS, SAGE II, HUFS, SARP). The r^2 between the SNP colored in purple (lead SNP from CAAPA MR-MEGA meta-analysis) and other SNPs in the data set were estimated using African American subjects in the CAAPA whole genome sequence reference panel.

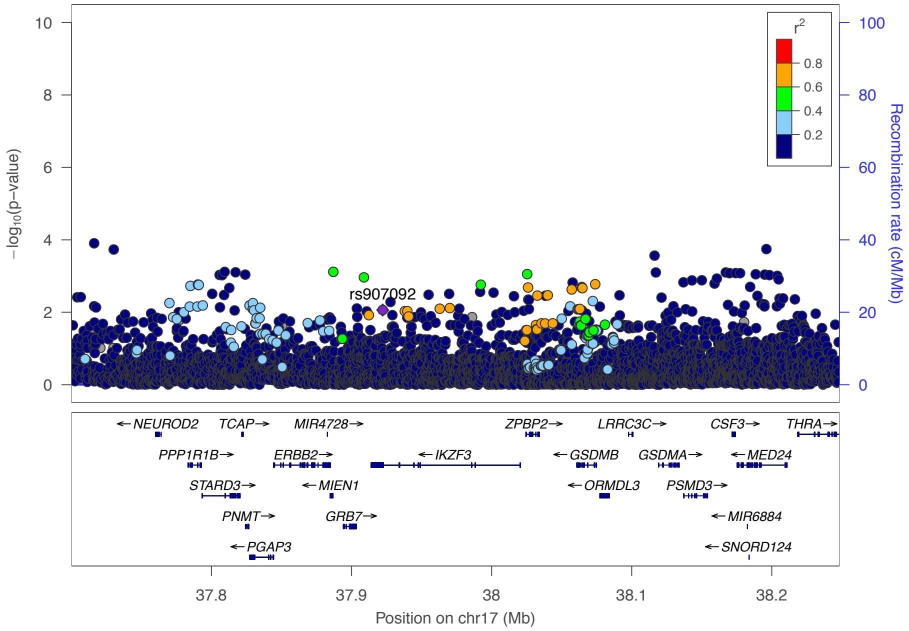

Supplementary Figure 12: Locus zoom plot of the chromosome 17q21 in studies with low African ancestry. Association statistics were generated by means of inverse-variance meta-analysis of CAAPA studies with mean African ancestry percentage < 50% (BIAS, PGCA, ProAR, GALA II). The r^2 between the SNP colored in purple (lead SNP from CAAPA MR-MEGA meta-analysis) and other SNPs in the data set were estimated using African American subjects in the CAAPA whole genome sequence reference panel.

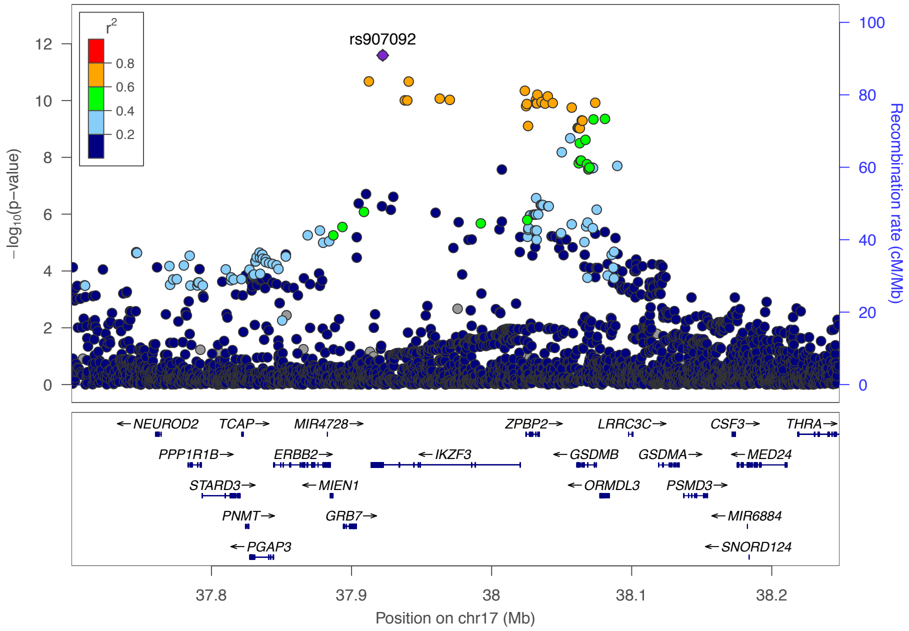

Supplementary Figure 13: Forest plots of SNPs from previous reports of African ancestry-specific associations in CAAPA. The chromosome 1 SNP was reported by EVE, and CAAPA studies included in the EVE meta-analysis discovery is indicated with a *. The chromosome 9 SNP was reported by eMERGE. RA/EA = Reference Allele/Effect Allele.

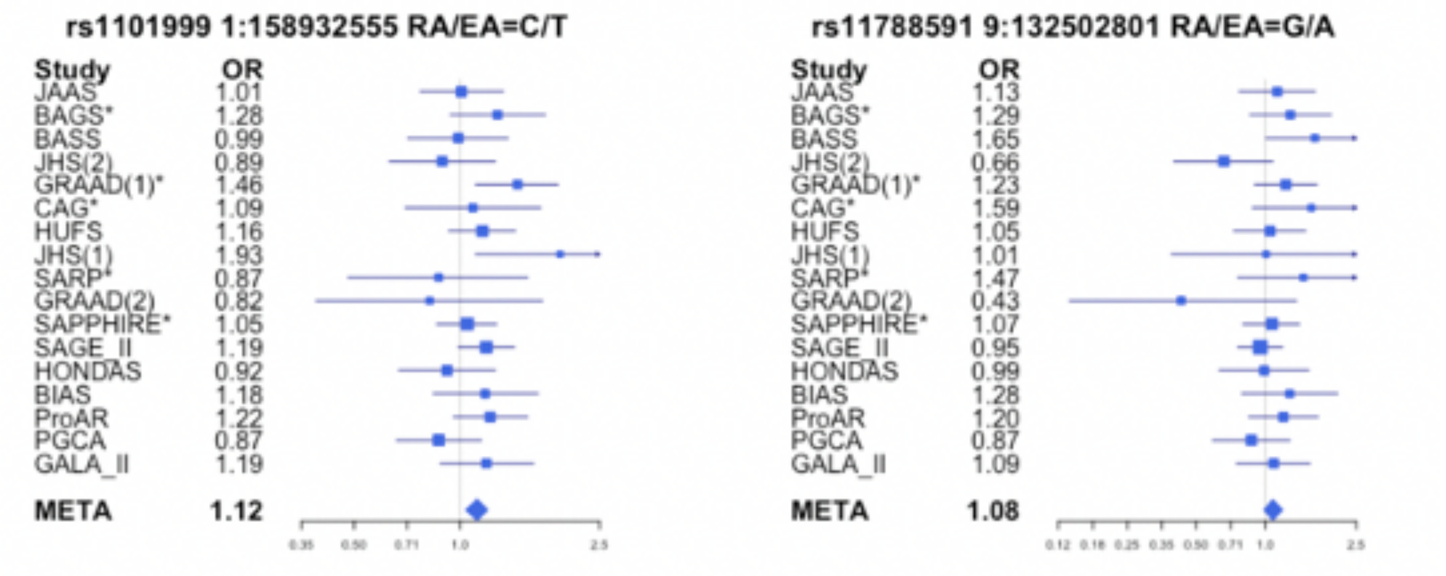

Supplementary Figures 14: Forest plots of the TAGC lead SNP associations in CAAPA. RA/EA = Reference Allele/Effect Allele.

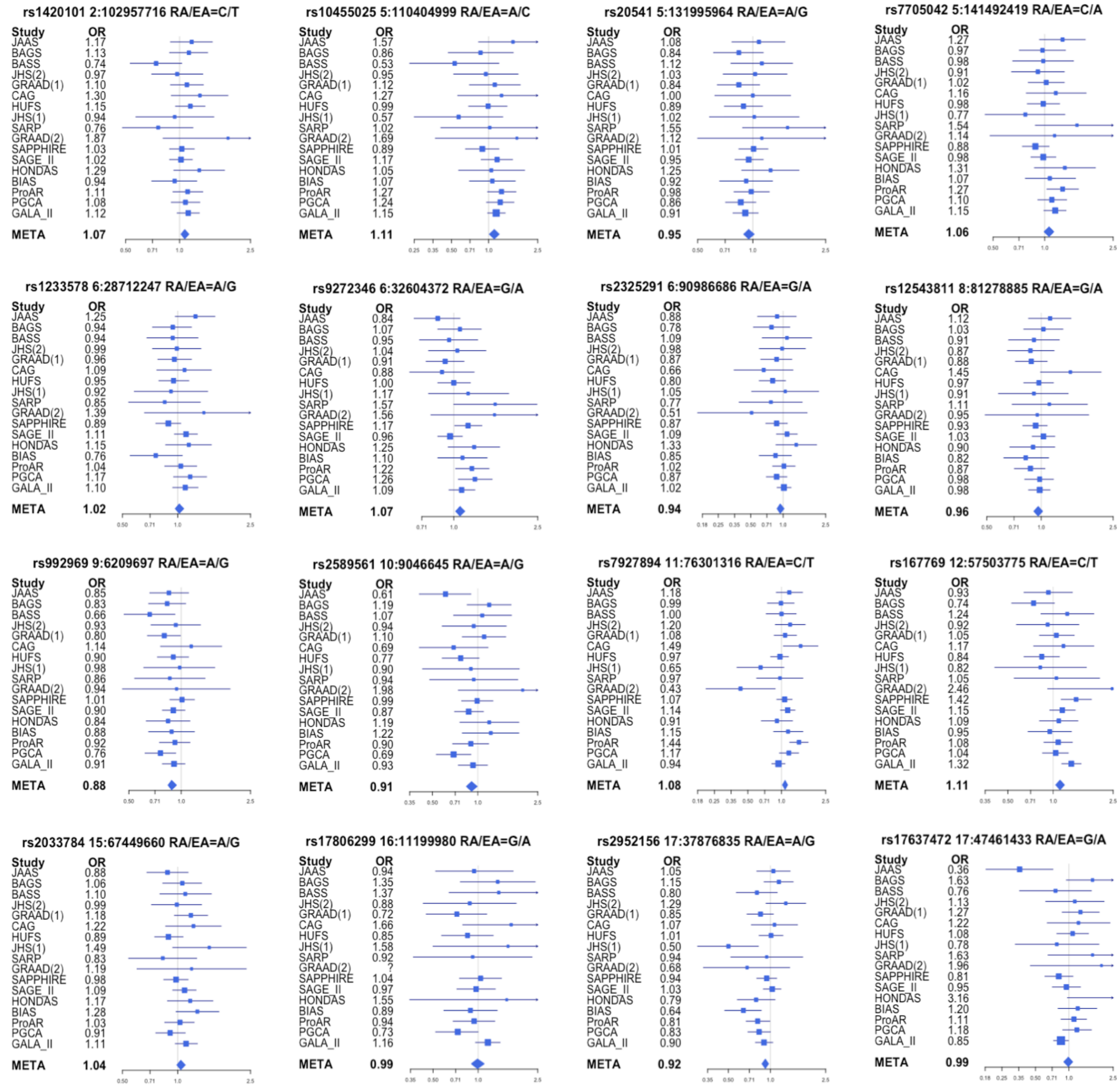

Supplementary Figures 15: Forest plots of the CAAPA meta-analysis lead SNPs with p-values < 10^{-6}. RA/EA = Reference Allele/Effect Allele.

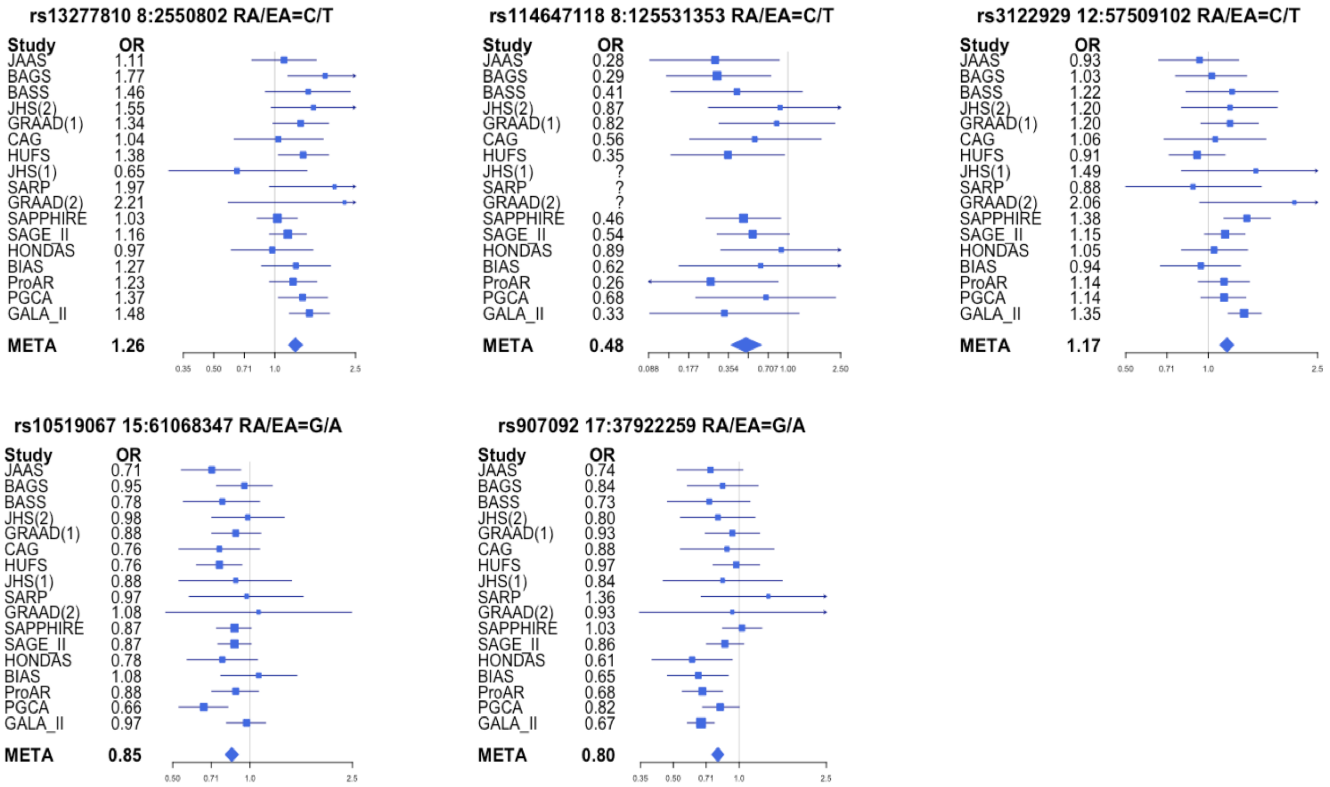

Supplementary Figures 16: Forest plots of the lead SNP in the admixture mapping peak. The plot is split by studies included in the admixture mapping discovery group (Discovery) and studies not included in the discovery (Non-discovery). RA/EA = Reference Allele/Effect Allele.

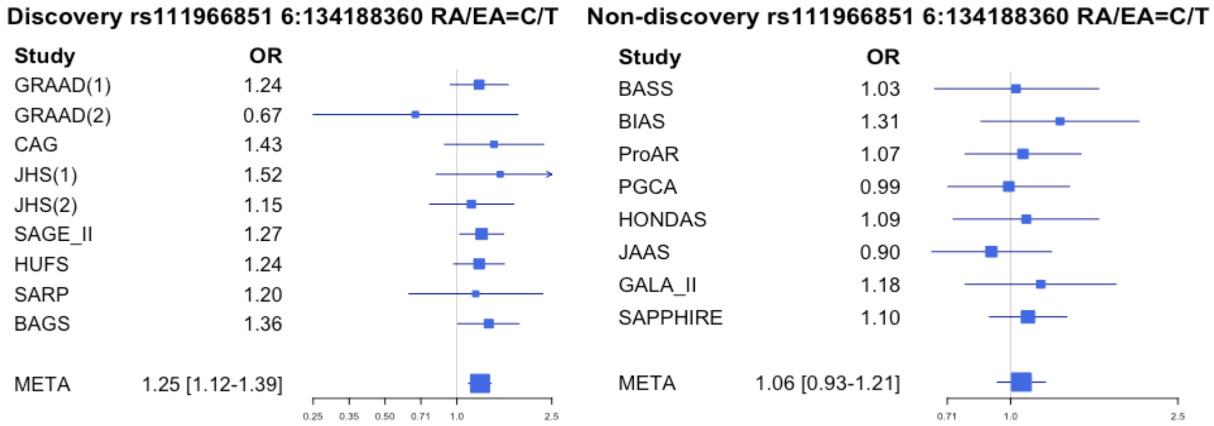

Supplementary Figures 17: Forest plots for 22 putative causal loci in the chromosome 17q12-21 locus. The 17 SNPs discussed by Stein et. al. (doi 10.1016/j.jaci.2017.12.974) were selected for this analysis, and an additional 5 SNPs from the CAAPA MR-MEGA meta-analysis, with $p < 10^{-6}$ and $r^2 < 0.8$ with all 17 selected SNPs in the 1000 Genomes Project European and African populations, were also included.

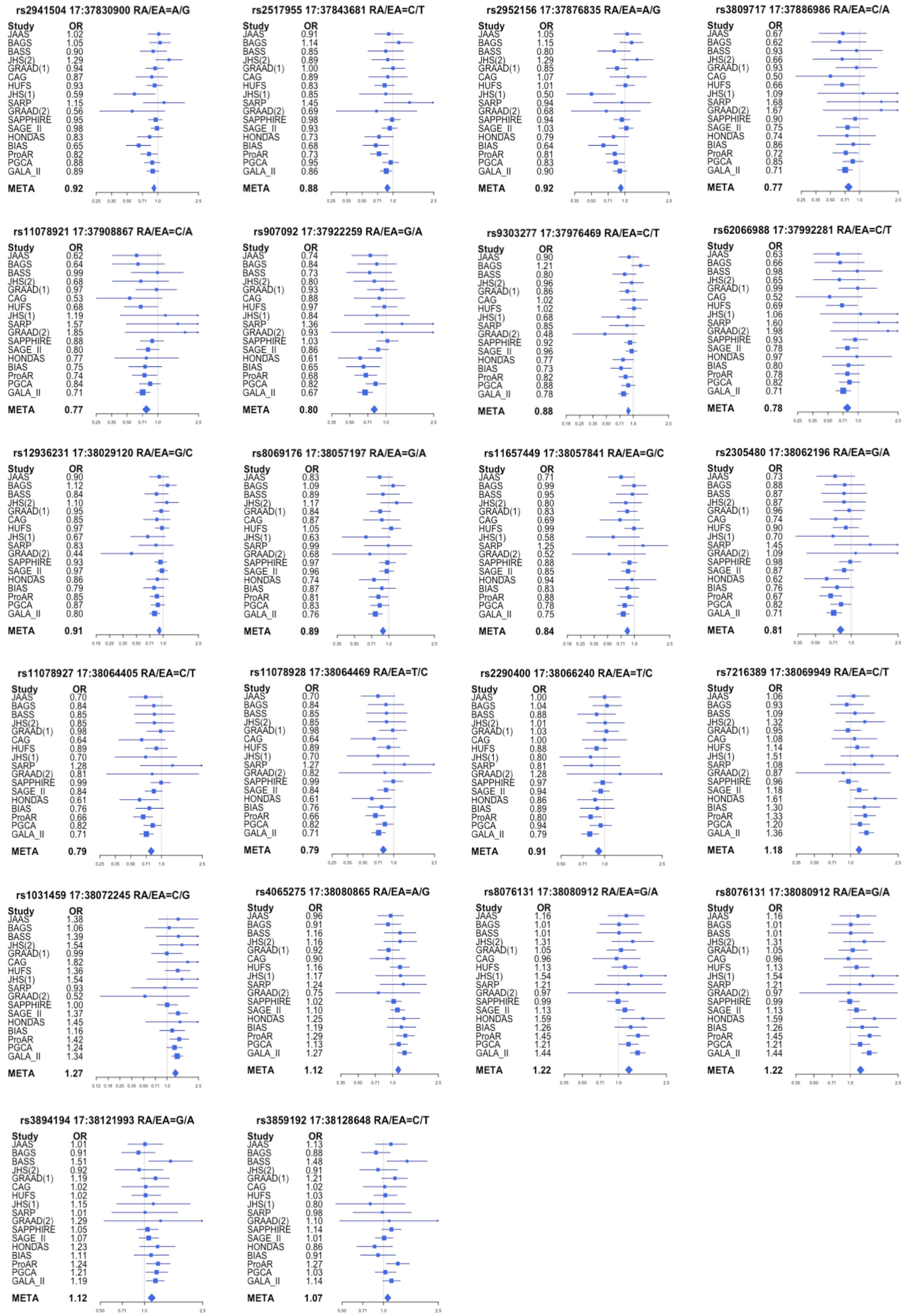

Supplementary Figures 18: Linkage disequilibrium (LD) between 22 putative causal loci in the chromosome 17q12-21 locus. The 17 SNPs discussed by Stein et. al. (doi 10.1016/j.jaci.2017.12.974) were selected for this analysis, and an additional 5 SNPs from the CAAPA MR-MEGA meta-analysis, with $p < 10^{-6}$ and $r^2 < 0.8$ with all 17 selected SNPs in the 1000 Genomes Project European (TGP) and African populations, were also included. The r^2 between each SNP is shown. CAAPA data sets have been ordered by decreasing proportion of African ancestry, estimated using ADMIXTURE. LD between SNPs is also shown for the TGP YRI and CEU populations.

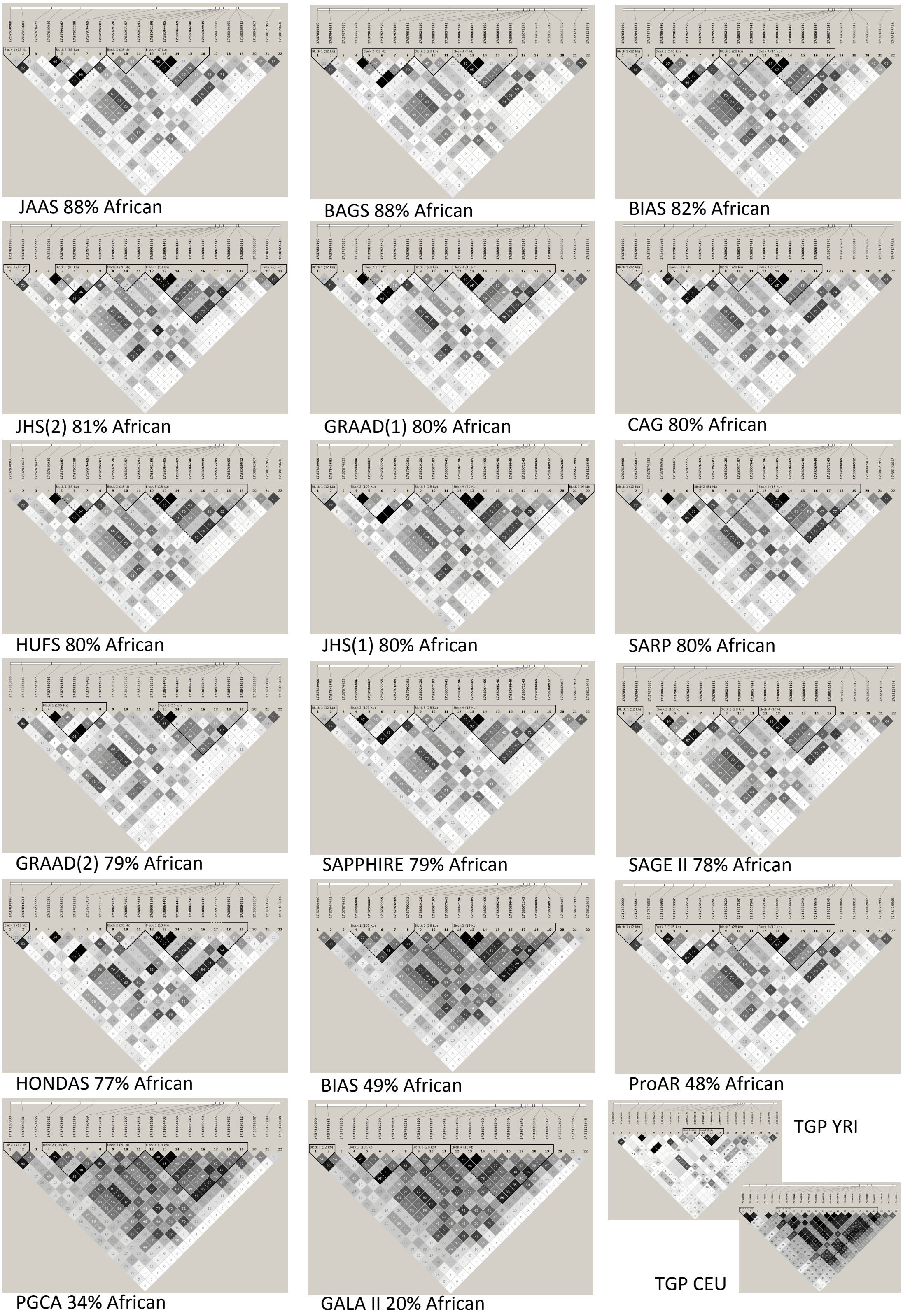

Supplementary Figures 19: Visualization of long-range chromatin interactions centered on the most significant SNP in the chromosome 8p23 CAAPA MR-MEGA meta-analysis results. This visualization was created using the Hi-C Unifying Genomic Interrogator (HUGIn) online tool. There is evidence for long-range interactions in three relevant tissue types (lymphoblastoid cells, fetal lung fibroblast cells, lung tissue).

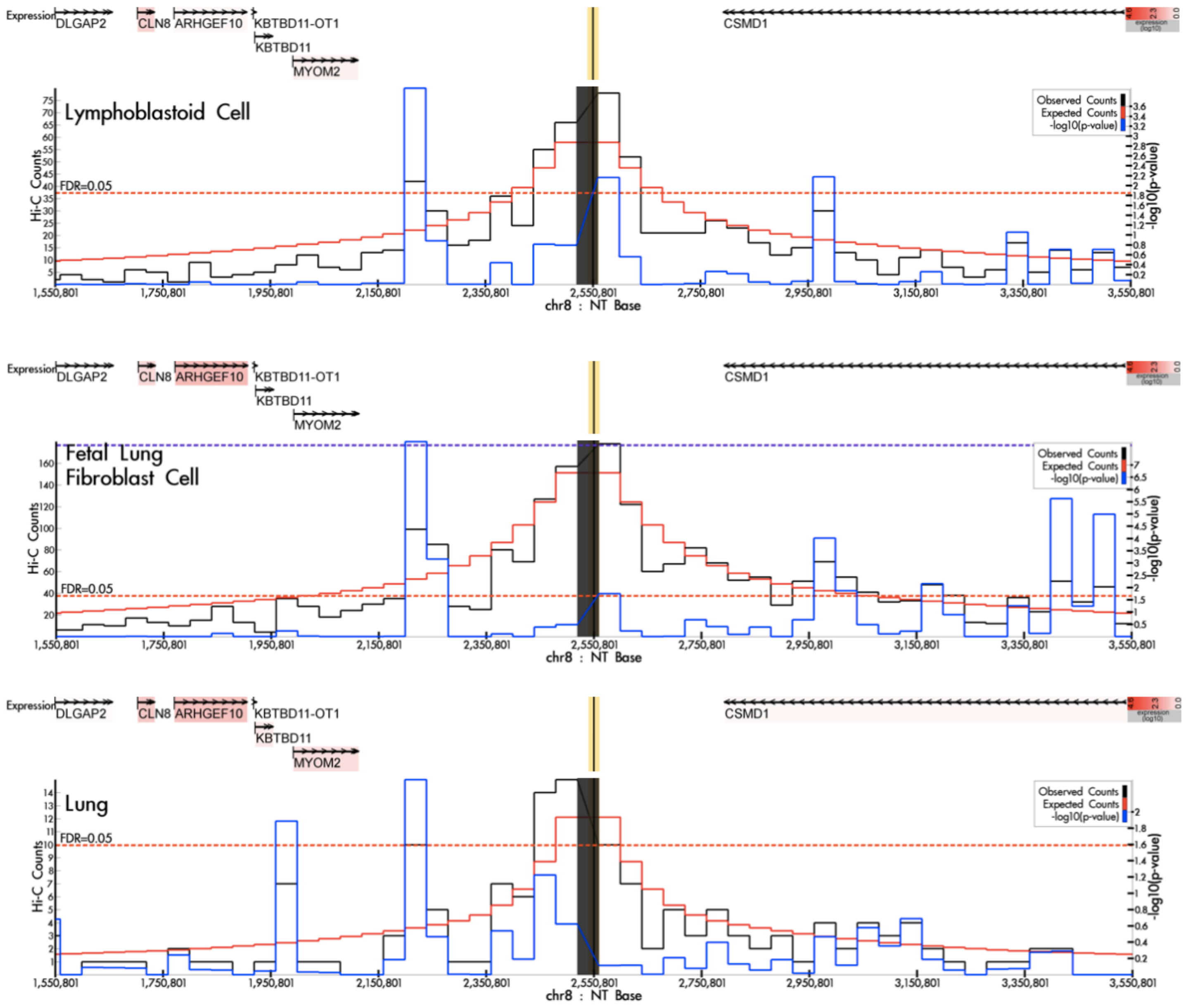

Supplementary Figure 20: Summary of the distribution of admixture mapping p-values. A QQ plot is shown in the top panel, followed by a histogram of p-values, and the Manhattan plot is shown in the bottom panel. The inflation factor was calculated by transforming p-values to 1 degree of freedom (df) Chi-square statistics, and dividing the median of these statistics by the median of the theoretical Chi-square (1 df) distribution. The vertical red line in the histogram indicates the median p-value, and the horizontal red line represent the theoretical uniform distribution that the p-values are expected to follow. The horizontal red line in the Manhattan plot represents the p-value significance threshold of 1.9×10^{-4}.

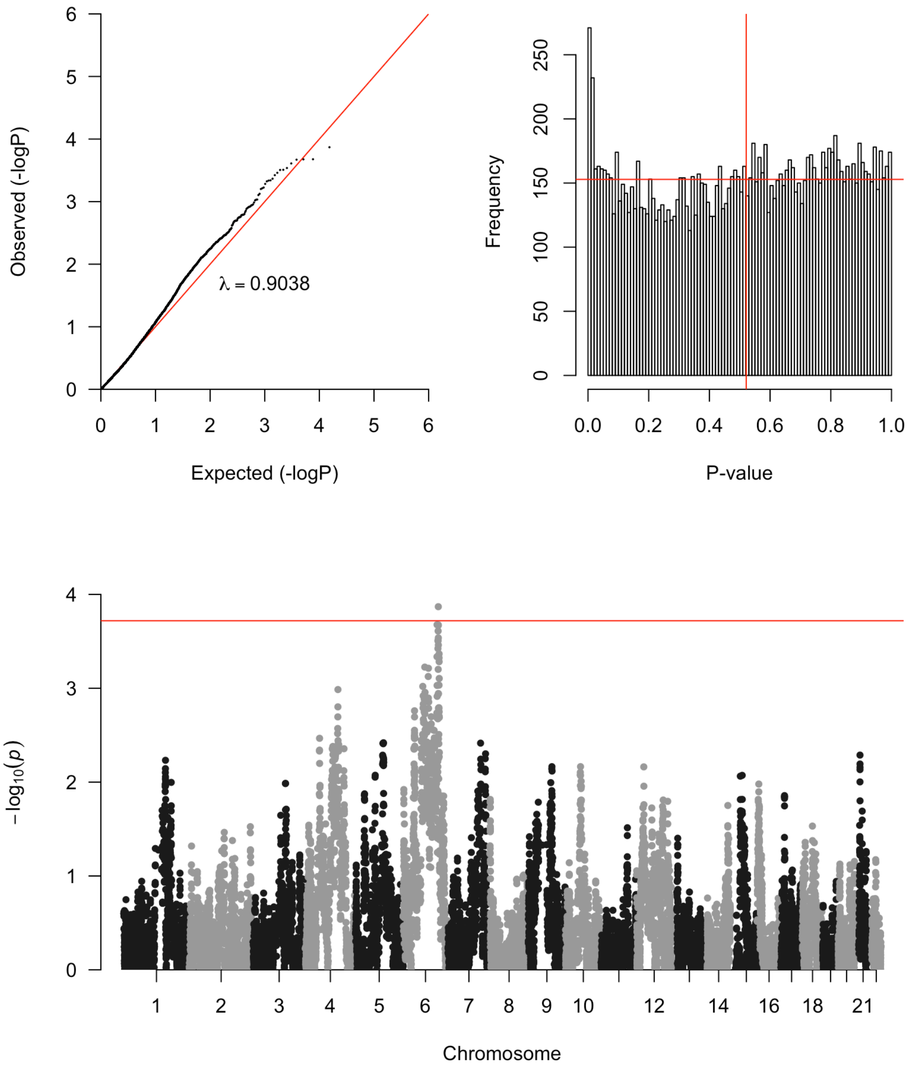

Supplementary Figure 21: The mean proportion of Africa ancestry across the genome of 7,146 CAAPA subjects, for the 15,824 local ancestry segments inferred by RFMix. The mean local African ancestry for a segment is the proportion of haplotypes called as African for that segment.

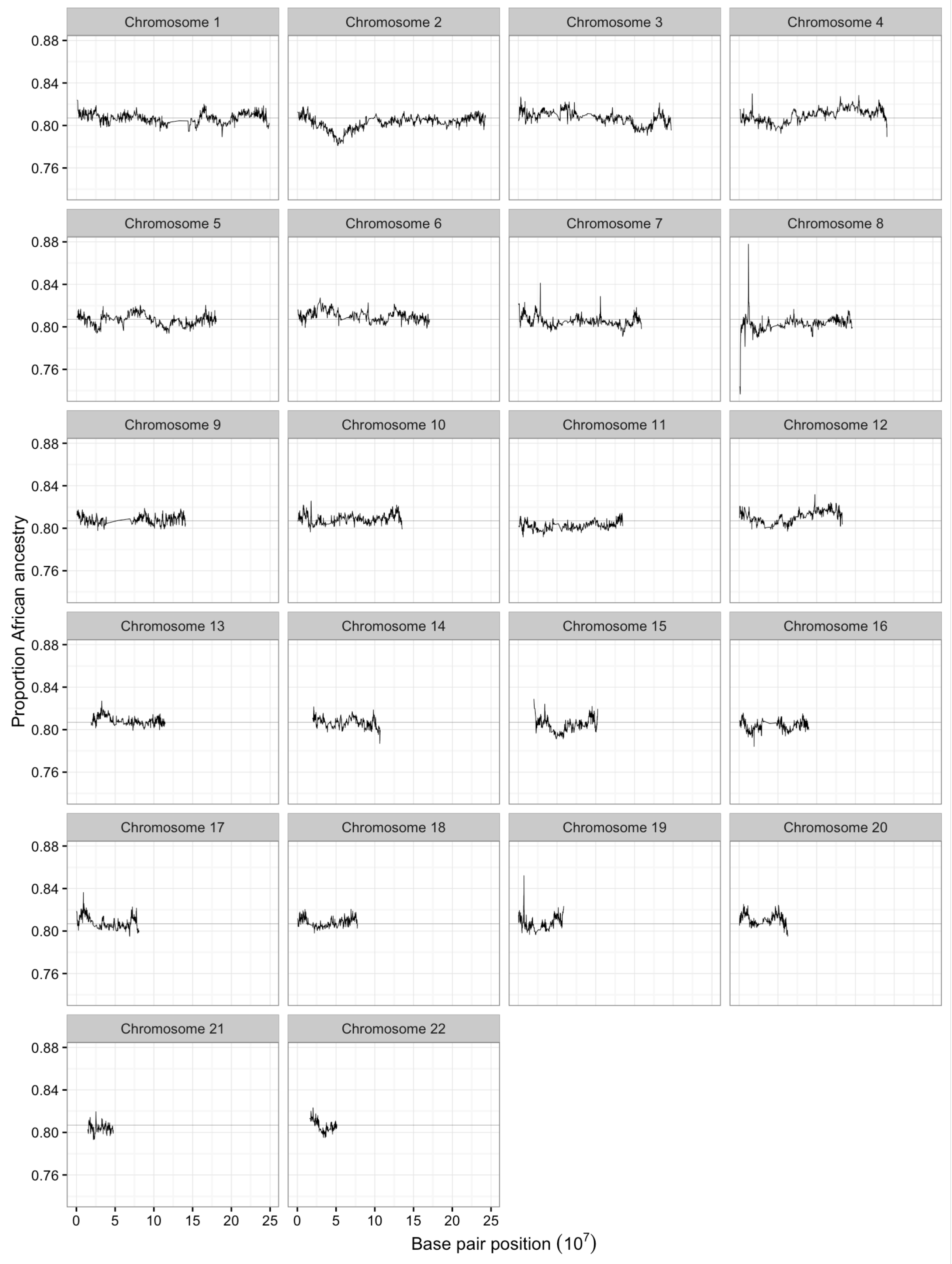

Supplementary Figure 22: Histogram of the standardized deviations between mean local African ancestry and mean genome-wide African ancestry, for the 15,824 local ancestry segments inferred by RFMix. The mean local African ancestry for a segment is the proportion of haplotypes called as African for that segment. The mean genome-wide African ancestry was calculated by dividing the number of SNPs called by RFMix as African, by the total number of SNPs for which local ancestry was called, across all genomes.

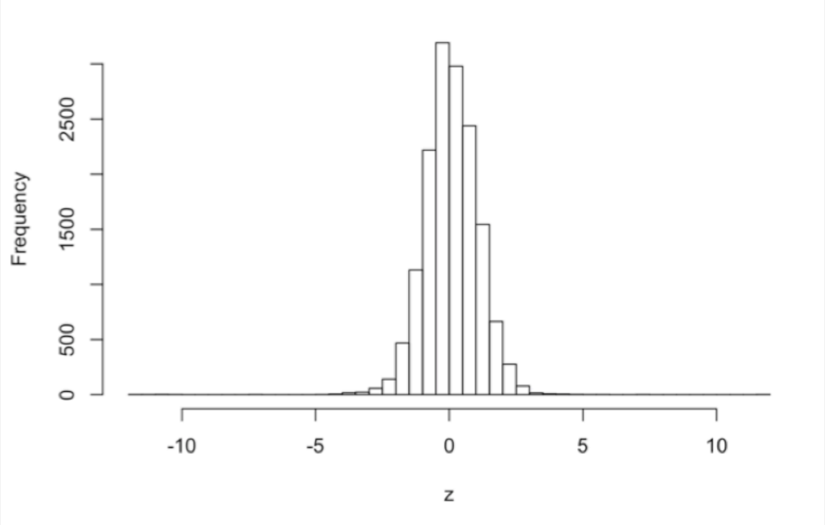

Supplementary Figure 23: Histogram of the differences between genome-wide ancestry estimated using ADMIXTURE, and genome-wide ancestry estimated using RFMix. The difference (delta) was calculated for each of the 7,416 individuals for which local ancestry was called. The per-individual RFMix mean genome-wide African ancestry was calculated by dividing the number of SNPs called by RFMix as African, by the total number of SNPs for which local ancestry was called across the genome, for that individual.

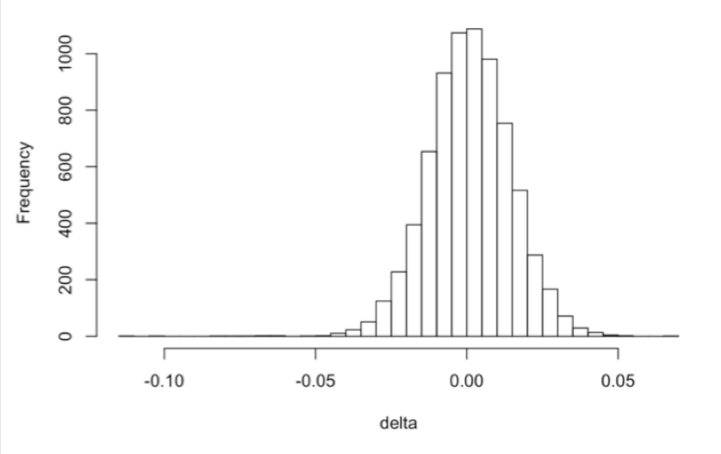

Supplementary Figure 24: Forest plot comparing genetic risk scores built using genome-wide significant SNPs from TAGC in CAAPA cases compared to controls. Genome-wide significant SNPs in TAGC Europeans (SNPs with fixed effect p-values < $5x10^{-8}$) were intersected with SNPs available in all the CAAPA studies and the lead (most significant) SNP in each of the broader 18 loci were used to build a genetic risk score in CAAPA subjects. The genetic risk score was set to the sum of the number of risk alleles that an individual carries, and the contribution of each SNP was weighted by 1/(TAGC fixed effect odds ratio). Logistic regression was used to test for the association between asthma (case or control status) and risk score separately for each study, and the results were combined using inverse-variance meta-analysis.

Study	OR [95% CI]	%YRI	%CEU	%NAT
JAAS	1.04 [0.96–1.13]	88%	11%	1%
BAGS	0.99 [0.92–1.07]	88%	11%	1%
BASS	1.09 [0.99–1.20]	82%	17%	1%
JHS(2)	1.04 [0.95–1.15]	81%	18%	1%
GRAAD(1)	1.04 [0.98–1.11]	80%	18%	1%
CAG	1.13 [1.00–1.26]	80%	18%	1%
HUFS	1.05 [1.00–1.12]	80%	19%	1%
JHS(1)	1.01 [0.87–1.16]	80%	19%	1%
SARP	1.07 [0.94–1.22]	80%	19%	1%
GRAAD(2)	0.96 [0.76–1.21]	79%	19%	2%
SAGE_II	1.06 [1.01–1.11]	77%	21%	2%
HONDAS	1.13 [1.04–1.23]	77%	3%	20%
BIAS	1.10 [1.01–1.19]	49%	40%	11%
ProAR	1.18 [1.11–1.25]	48%	46%	6%
PGCA	1.15 [1.09–1.22]	34%	37%	29%
GALA_II	1.09 [1.05–1.13]	20%	66%	14%
**META**	**1.08 [1.06–1.10]**			

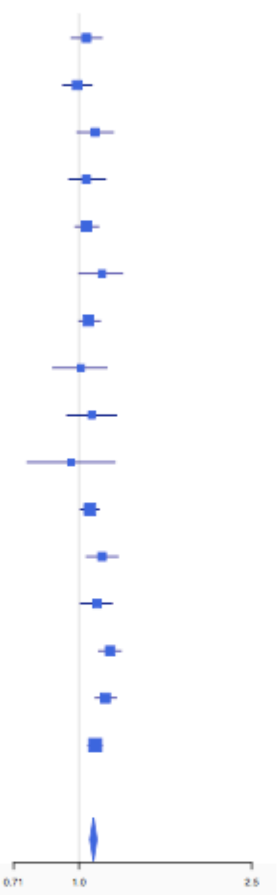

Supplementary Tables

Supplementary Table 1: Clinical characteristics summary. The distribution of age, sex, age of asthma onset and total serum IgE (where available) is summarized in this table by dataset used in the meta-analysis. SEM=standard error of the mean. Childhood onset asthma is defined as having been diagnosed with asthma before 16 years of age. The information summarized in this table was not available for all subjects included in the association analysis; therefore the number of subjects (n) used to calculate these statistics is given in brackets.

Study	Phenotype	Mean Age (+- SEM)	%Male	%Childhood onset asthma	Total Serum IgE in IU ml^{-1} (SEM) *
**BASS**	Non-asthmatic	30.6 (+-0.5) (n=216)	52.3% (n=216)		
	Asthmatic	29.9 (+-0.6) (n=135)	30.4% (n=135)	71.9% (n=128)	
**GRAAD(1)**	Non-asthmatic	35 (+-0.9) (n=385)	39.2% (n=385)		59.1 (+-1.1) (n=378)
	Asthmatic	23.5 (+-0.9) (n=396)	46.2% (n=396)	68.8% (n=109)	126.9 (+-1.1) (n=359)
**GRAAD(2)**	Non-asthmatic	38.7 (+-2.6) (n=23)	30.4% (n=23)		
	Asthmatic	44 (+-1.5) (n=64)	27.7% (n=65)	55.7% (n=61)	
**CAG**	Non-asthmatic	38 (+-0.8) (n=156)	26.3% (n=156)		29.9 (+-1.2) (n=127)
	Asthmatic	23.9 (+-1.8) (n=114)	45.6% (n=114)	85.1% (n=114)	180.7 (+-1.2) (n=102)
**SAPPHIRE**	Non-asthmatic	39.1 (+-0.6) (n=485)	34.8% (n=485)		
	Asthmatic	32.6 (+-0.4) (n=1,243)	36.0% (n=1,243)	69.0% (n=1,243)	202 (+-1.1) (n=210)
**JHS(1)**	Non-asthmatic	65.9 (+-0.3) (n=283)	29.7% (n=283)		
	Asthmatic	66 (+-0.9) (n=44)	15.9% (n=44)	26.8% (n=41)	
**JHS(2)**	Non-asthmatic	49.5 (+-0.5) (n=546)	37.7% (n=546)		
	Asthmatic	49.6 (+-1.2) (n=101)	24.8% (n=101)	48.3% (n=89)	
**SAGE II**	Non-asthmatic	18.4 (+-0.3) (n=690)	42.5% (n=691)		
	Asthmatic	14.7 (+-0.2) (n=998)	50.6% (n=1001)	97.1% (n=837)	
**HUFS**	Non-asthmatic	39.6 (+-0.4) (n=1527)	41% (n=1527)		
	Asthmatic	38.1 (+-0.9) (n=303)	31.7% (n=303)	61.1% (n=293)	
**SARP**	Non-asthmatic	36 (+-1.6) (n=45)	26.7% (n=45)		
	Asthmatic	31.2 (+-0.8) (n=302)	42.4% (n=302)	69.9% (n=296)	187.2 (+-1.1) (n=240)
**BAGS**	Non-asthmatic	37.3 (+-0.9) (n=338)	46.7% (n=338)		117.2 (+-1.1) (n=311)
	Asthmatic	22.7 (+-0.8) (n=282)	49.3% (n=282)	81.2% (n=245)	311.3 (+-1.1) (n=270)
**BIAS**	Non-asthmatic	27.5 (+-0.9) (n=426)	45.1% (n=426)		425.6 (+-1.1) (n=417)
	Asthmatic	26.9 (+-1.3) (n=194)	46.4% (n=194)		542.7 (+-1.1) (n=191)
**ProAR**	Non-asthmatic		13.9% (n=346)		
	Asthmatic		20.5% (n=761)		
**PGCA**	Non-asthmatic	32.2 (+-0.8) (n=488)	47.5% (n=488)		52.6 (+-1.1) (n=377)
	Asthmatic	30.9 (+-0.7) (n=662)	29.1% (n=664)		201.8 (+-1) (n=657)
**HONDAS**	Non-asthmatic	16.4 (+-0.8) (n=205)	32.9% (n=249)		
	Asthmatic	14.1 (+-0.8) (n=158)	38.6% (n=254)		
**JAAS**	Non-asthmatic	18.8 (+-0) (n=506)	48.5% (n=507)		50.5 (+-1.1) (n=506)
	Asthmatic	18.8 (+-0) (n=167)	46.1% (n=167)		92.9 (+-1.1) (n=167)
**GALA II**	Non-asthmatic	13.5 (+-0.1) (n=853)	46.8% (n=853)		
	Asthmatic	12.4 (+-0.1) (n=900)	55.4% (n=901)	99.7% (n=717)	

* Geometric mean and SEM

Supplementary Table 2: Summary of ancestry distribution in the CAAPA datasets. The median and interquartile range (IQR) and mean and standard error of the mean (SEM) of the proportion of African ancestry, estimated using the software program ADMIXTURE, are listed by dataset and asthma case-control status. The p-values of univariate association tests between asthma case-control status and the first and second principal component (PC1 and PC2), where the principal components were generated separately in each data set without reference populations, are also shown.

	Proportion African Ancestry					
	Cases		Controls		PC1	PC2
	IQR	Mean (± SEM)	IQR	Mean (± SEM)		
**BASS**	0.83 [0.77-0.89]	0.82 (+-0.01)	0.83 [0.77-0.87]	0.82 (+-0.01)	0.964	0.421
**GRAAD(1)**	0.82 [0.76-0.87]	0.80 (+-0.01)	0.83 [0.76-0.88]	0.81 (+-0.00)	0.235	0.189
**GRAAD(2)**	0.83 [0.76-0.86]	0.81 (+-0.01)	0.77 [0.68-0.88]	0.75 (+-0.03)	0.174	0.221
**CAG**	0.82 [0.76-0.86]	0.80 (+-0.01)	0.83 [0.76-0.87]	0.80 (+-0.01)	0.996	0.232
**SAPPHIRE**	0.81 [0.74-0.85]	0.79 (+-0.00)			0.375	0.601
**JHS(1)**	0.85 [0.79-0.88]	0.83 (+-0.01)	0.82 [0.75-0.88]	0.80 (+-0.01)	0.046	0.330
**JHS(2)**	0.84 [0.79-0.89]	0.83 (+-0.01)	0.83 [0.76-0.88]	0.81 (+-0.00)	0.022	0.104
**SAGE II**	0.81 [0.74-0.85]	0.77 (+-0.00)	0.80 [0.73-0.85]	0.77 (+-0.00)	0.499	0.633
**HUFS**	0.81 [0.75-0.87]	0.79 (+-0.01)	0.82 [0.75-0.88]	0.80 (+-0.00)	0.817	0.310
**SARP**	0.83 [0.75-0.87]	0.80 (+-0.01)	0.83 [0.78-0.87]	0.80 (+-0.02)	0.969	0.534
**BAGS**	0.90 [0.85-0.94]	0.88 (+-0.01)	0.90 [0.84-0.94]	0.88 (+-0.01)	0.896	0.823
**BIAS**	0.50 [0.42-0.62]	0.51 (+-0.02)	0.47 [0.36-0.59]	0.47 (+-0.01)	0.704	0.002
**ProAR**	0.49 [0.33-0.60]	0.47 (+-0.01)	0.50 [0.37-0.62]	0.50 (+-0.01)	0.024	0.196
**PGCA**	0.34 [0.23-0.46]	0.35 (+-0.01)	0.29 [0.19-0.43]	0.32 (+-0.01)	0.001	0.018
**HONDAS**	0.81 [0.79-0.83]	0.77 (+-0.01)	0.81 [0.79-0.82]	0.76 (+-0.01)	0.413	4e-07
**JAAS**	0.89 [0.83-0.93]	0.87 (+-0.01)	0.90 [0.84-0.94]	0.88 (+-0.00)	0.093	0.800
**GALA II**	0.18 [0.13-0.26]	0.21 (+-0.00)	0.17 [0.13-0.24]	0.20 (+-0.00)	0.004	0.027

Supplementary Table 3: CAAPA whole genome sequence (WGS) reference panel. This table summarizes the ethnicity and recruitment sites of subjects used to build the CAAPA WGS reference panel.

Ethnicity & Site	Study	Total (% Male)	Asthmatics (% Male)	Non-asthmatics (% Male)
African American (Atlanta)	COPDGene	50 (66%)	25 (64%)	25 (68%)
African American (Chicago)	CAG	50 (34%)	25 (44%)	25 (24%)
African American (Baltimore-DC)	GRAAD	48 (50%)	24 (50%)	24 (50%)
African American (Nashville)	BREATHE/VALID	49 (45%)	25 (40%)	24 (50%)
African American (NYC)	REACH	50 (54%)	20 (55%)	30 (53%)
African American (Detroit)	SAPPHIRE	49 (35%)	24 (46%)	25 (24%)
African American (San Francisco)	SAGE II	50 (60%)	25 (72%)	25 (48%)
African American (Winston-Salem)	SARP	50 (10%)	25 (8%)	25 (12%)
African American (Jackson)	JHS	50 (52%)	25 (52%)	25 (52%)
Barbados	BAGS	47 (49%)	23 (48%)	22 (50%)
Jamaica	JAAS	50 (50%)	25 (52%)	25 (48%)
Dominican Republic (New York, Texas)	GALA II	47 (40%)	22 (45%)	25 (36%)
Honduras	HONDAS	43 (51%)	22 (55%)	21 (48%)
Colombia	PGCA	50 (48%)	26 (50%)	24 (46%)
Puerto Rico	GALA II	53 (45%)	28 (61%)	25 (28%)
Brazil	BIAS	39 (44%)	7 (29%)	32 (47%)
Nigeria	AEGS	45 (49%)	23 (30%)	22 (68%)
Gabon	Leiden University	28 (50%)		
Palenque (San Basilio de Palenque)	University of Cartagena	32 (63%)		
**Total**		**880**	**394**	**424**

Abbreviations: ***BAGS***, Barbados Asthma Genetics Study; ***BIAS***, Brazilian Immunogenetics of Asthma & Schistosomiasis study (Brazil, rural); ***CAG***, Chicago Asthma Genetics; ***COPDGene***, Genetic Epidemiology of COPD study (Atlanta); ***GALA II***; Genetics of Asthma in Latino Americans (Dominican, Puerto Rican); ***GRAAD***, Genomic Research on Asthma in the African Diaspora (Baltimore); ***JAAS***, Jamaican Adolescent Asthma Study; ***JHS***, Jackson Heart Study (Jackson, Mississippi); ***PGCA***, Proyecto Genes Candidatos en Asma (Colombia); ***REACH***, Reducing Emergency Asthma Care in Harlem (NYC); ***SAGE II***, Study of African Americans, Asthma, Genes, & Environments (San Francisco); ***SAPPHIRE***, Study of Asthma Phenotypes and Pharmacogenomics Interactions by Race-ethnicity (Detroit); ***SARP***, Severe Asthma Research Program (Winston-Salem, Atlanta)

Supplementary Table 4: Suggestive asthma associations in African Americans. Logistic regression results were combined using inverse-variance meta-analysis (METAL software), and associations with p-values<10^{-6} where 3 or more datasets contributed to the association test, are shown.

Chromosome: hg19 position	RA/ EA	EAF**	OR [95% CI]	P-value	Direction*
1:234597344	T/C	0.0093	0.27 [0.16-0.45]	9.53E-07	??-???---?
1:234597720	A/G	0.0093	3.74 [2.21-6.33]	9.28E-07	??+???+++?
4:130020791	T/C	0.432	0.83 [0.77-0.89]	3.08E-07	----------
4:130022069	T/C	0.4321	1.21 [1.12-1.30]	3.20E-07	++++++++++
4:130022161	A/C	0.4321	1.21 [1.12-1.30]	3.21E-07	++++++++++
4:130022347	T/C	0.4321	1.21 [1.12-1.30]	3.34E-07	++++++++++
4:130022448	A/G	0.4322	1.21 [1.12-1.30]	3.42E-07	++++++++++
4:130022875	A/C	0.4322	1.21 [1.12-1.30]	3.56E-07	++++++++++
4:130023759	A/T	0.4323	0.83 [0.77-0.89]	3.83E-07	----------
4:130025037	T/G	0.4325	0.83 [0.77-0.89]	4.60E-07	----------
4:130025873	A/T	0.4326	1.21 [1.12-1.30]	5.25E-07	++++++++++
4:130028412	T/C	0.3052	1.22 [1.13-1.32]	9.01E-07	+++-++++++
4:130028426	T/G	0.3052	1.22 [1.13-1.32]	9.12E-07	+++-++++++
4:130029879	A/C	0.4328	0.83 [0.77-0.90]	9.99E-07	----------
4:130030652	A/G	0.4592	0.83 [0.78-0.90]	7.55E-07	----------
4:130031498	T/G	0.4592	0.83 [0.78-0.90]	8.24E-07	----------
4:130040437	T/C	0.3953	0.82 [0.76-0.88]	1.83E-07	----------
4:130042232	A/C	0.3985	0.83 [0.77-0.89]	7.01E-07	----------

* Datasets were included in the meta-analysis in the following order:
BASS, CAG, SAPPHIRE, JHS(1), JHS(2), GRAAD(2), GRAAD(1), SAGE, HUFS, SARP
RA/EA=Reference Allele/Effect Allele
EAF=Effect Allele Frequency
** EAF is the weighted MAF of the African Americans, with weights equal to the effective sample size

Supplementary Table 5: Suggestive asthma associations in Brazilians from Salvador (ProAR). Associations with p-values<10^{-6} are shown.

Chromosome: hg19 position	RA/ EA	EAF	RSQ	OR [95% CI]	P-value
6:82773485	A/T	0.1287	0.7036	0.44 [0.32-0.61]	7.86e-07
9:12163051	C/G	0.0100	0.6466	0.05 [0.02-0.16]	3.42e-07
12:99656329	G/A	0.0104	0.5272	0.05 [0.01-0.16]	9.74e-07
12:99662931	A/G	0.0106	0.5181	0.05 [0.01-0.16]	8.94e-07
12:99665733	T/C	0.0106	0.5140	0.05 [0.01-0.16]	9.83e-07
17:72963287	G/A	0.9261	0.6886	0.34 [0.22-0.53]	8.09e-07
20:4196076	G/A	0.0067	0.3898	0.01 [0.00-0.07]	7.58e-07

RA/EA=Reference Allele/Effect Allele
EAF=Effect Allele Frequency
RSQ=Imputation quality metric from Minimac; the estimated squared correlation between the imputed and true genotype

Supplementary Table 6: Suggestive asthma associations in Colombians (PGCA). Associations with p-values<10^{-6} are shown.

Chromosome: hg19 position	RA/ EA	EAF	RSQ	OR [95% CI]	P-value
5:31688809	G/A	0.1017	0.6701	0.41 [0.28-0.58]	9.72e-07
10:82625900	C/T	0.1192	0.3505	0.31 [0.20-0.49]	4.58e-07
11:4385885	C/G	0.2987	0.5211	1.95 [1.51-2.52]	3.31e-07
11:24231483	A/G	0.0314	0.8528	0.25 [0.14-0.43]	5.37e-07
11:24242691	A/G	0.0305	0.8441	0.25 [0.14-0.43]	8.86e-07
11:24244647	G/C	0.0313	0.8831	0.26 [0.15-0.44]	7.69e-07
11:24250241	G/A	0.0464	0.9166	0.34 [0.23-0.52]	6.46e-07
15:101949607	A/G	0.0394	0.6982	0.26 [0.15-0.44]	7.81e-07

RA/EA=Reference Allele/Effect Allele
EAF=Effect Allele Frequency
RSQ=Imputation quality metric from Minimac; the estimated squared correlation between the imputed and true genotype

Supplementary Table 7: Suggestive asthma associations in Jamaicans (JAAS). Associations with p-values<10^{-6} are shown.

Chromosome: hg19 position	RA/ EA	EAF	RSQ	OR [95% CI]	P-value
1:231925824	G/A	0.9813	0.8759	0.05 [0.02-0.13]	1.04e-09
1:231927903	A/G	0.0185	0.8908	20.39 [7.69-54.04]	1.33e-09
1:231935538	C/T	0.0338	0.4450	22.63 [8.20-62.45]	1.72e-09
1:243674772	A/G	0.6900	0.9798	0.51 [0.39-0.67]	7.88e-07
1:243677834	G/A	0.6738	0.9787	0.51 [0.39-0.67]	7.02e-07
1:243686913	T/C	0.6934	0.9746	0.51 [0.39-0.67]	1.00e-06
2:51810688	G/C	0.0337	0.3408	19.60 [6.24-61.53]	3.46e-07
3:5462116	A/G	0.0193	0.9990	11.79 [4.75-29.23]	1.01e-07
4:54570700	G/A	0.0463	0.7808	5.07 [2.67-9.64]	7.21e-07
4:54585603	G/A	0.0449	0.6924	5.66 [2.84-11.27]	7.94e-07
4:62148294	T/A	0.0065	0.6713	136.39 [19.85-937.30]	5.78e-07
4:119333755	G/A	0.0096	0.5399	58.37 [11.78-289.16]	6.32e-07
5:149345213	T/C	0.1088	0.8752	3.45 [2.24-5.32]	2.06e-08
5:149353143	G/T	0.1075	0.9463	3.36 [2.21-5.12]	1.54e-08
5:149369835	C/G	0.1113	0.9442	3.21 [2.13-4.85]	2.93e-08
5:149376444	T/C	0.1074	0.9511	3.32 [2.18-5.05]	2.09e-08
5:149381419	A/T	0.1103	0.9613	3.39 [2.24-5.12]	7.64e-09
5:149389647	C/T	0.1165	0.9991	3.09 [2.07-4.60]	2.99e-08
5:149396004	T/C	0.1097	0.9427	3.21 [2.12-4.87]	3.71e-08
5:149419591	C/T	0.1102	0.9719	3.32 [2.20-5.02]	1.08e-08
5:149423839	G/A	0.1105	0.9600	3.36 [2.22-5.08]	9.08e-09
5:149424222	C/T	0.1105	0.9588	3.37 [2.23-5.09]	8.90e-09
5:149428096	C/T	0.1118	0.9463	3.35 [2.22-5.08]	1.04e-08
5:149428398	G/A	0.1118	0.9456	3.36 [2.22-5.08]	1.02e-08
5:149429256	T/C	0.1150	0.9234	3.30 [2.18-4.99]	1.57e-08
8:18489949	G/C	0.0097	0.9259	28.23 [7.44-107.12]	9.13e-07
8:106260968	C/A	0.0237	0.9366	8.48 [3.62-19.85]	8.32e-07
9:2391798	A/C	0.2689	0.9637	2.34 [1.75-3.13]	8.67e-09
9:2398806	A/C	0.2676	0.9749	2.27 [1.71-3.03]	2.01e-08
9:2399820	A/C	0.2331	0.9659	2.60 [1.92-3.53]	6.46e-10
9:2404016	T/G	0.2253	0.9648	2.55 [1.88-3.47]	1.85e-09
9:2407784	G/T	0.2657	0.9918	2.22 [1.67-2.95]	3.60e-08
9:2408221	G/T	0.2580	0.9943	2.17 [1.63-2.89]	1.04e-07
9:2408299	G/A	0.2579	0.9947	2.17 [1.63-2.89]	1.04e-07
9:2408685	C/G	0.2579	0.9961	2.17 [1.63-2.89]	1.04e-07
9:2408770	C/T	0.2723	0.9996	2.15 [1.62-2.86]	9.89e-08
9:2409037	C/G	0.2619	0.9971	2.17 [1.63-2.87]	8.51e-08
9:2410062	C/T	0.2621	0.9951	2.16 [1.62-2.86]	1.04e-07
9:2414058	C/T	0.2555	0.9944	2.35 [1.75-3.15]	1.04e-08
9:2414127	G/A	0.2181	0.9999	2.27 [1.67-3.09]	1.98e-07
9:2414288	T/C	0.2581	0.9616	2.12 [1.58-2.83]	3.89e-07
9:2417307	T/A	0.2278	0.9521	2.39 [1.75-3.26]	4.89e-08
9:2418370	C/A	0.2321	0.9468	2.31 [1.69-3.15]	1.33e-07
9:2421084	G/A	0.2347	0.9163	2.23 [1.63-3.06]	4.89e-07
9:2421273	G/A	0.2357	0.9083	2.24 [1.63-3.06]	4.89e-07
12:46542295	T/C	0.2339	0.9138	2.09 [1.56-2.81]	9.76e-07
12:126283645	T/C	0.2228	0.8622	2.20 [1.61-3.02]	9.63e-07
13:51345649	C/T	0.0536	0.5067	7.79 [3.58-16.93]	2.24e-07
13:51389076	G/C	0.0091	0.8171	38.08 [9.13-158.83]	5.88e-07
13:51425812	G/T	0.0093	0.7614	44.75 [10.42-192.11]	3.17e-07
15:36247851	C/T	0.0158	0.7933	18.09 [5.94-55.08]	3.45e-07
15:36251876	C/T	0.0158	0.7957	17.54 [5.76-53.37]	4.52e-07
15:36252279	G/A	0.0155	0.8046	17.72 [5.80-54.16]	4.57e-07
15:82458849	T/C	0.0086	0.8680	37.43 [8.79-159.44]	9.60e-07
15:82474184	C/A	0.0092	0.7982	40.20 [9.41-171.73]	6.15e-07
15:82481322	C/T	0.0095	0.7810	40.76 [9.59-173.23]	5.10e-07
15:83241267	A/G	0.0105	0.7286	44.43 [10.73-183.96]	1.67e-07
15:83260546	C/T	0.0109	0.7135	44.39 [10.84-181.82]	1.35e-07
15:83264378	A/C	0.0109	0.7101	43.81 [10.72-179.10]	1.43e-07
15:83268679	A/C	0.0110	0.7086	43.49 [10.65-177.59]	1.47e-07
15:83275248	C/A	0.0110	0.7060	42.65 [10.49-173.52]	1.58e-07
15:83324896	C/T	0.0124	0.5920	45.29 [10.80-189.90]	1.85e-07

Chromosome: hg19 position	RA/ EA	EAF	RSQ	OR [95% CI]	P-value
15:83329482	C/A	0.0121	0.5948	47.08 [11.04-200.66]	1.92e-07
15:83341607	A/G	0.0151	0.4759	45.38 [10.73-191.97]	2.17e-07
15:83379048	C/T	0.0126	0.5436	44.78 [10.25-195.57]	4.31e-07
15:83383545	C/T	0.0160	0.4546	41.62 [10.04-172.52]	2.76e-07
17:28516471	T/C	0.0088	0.4015	281.62 [33.05-2399.84]	2.47e-07
17:71813734	A/C	0.0267	0.9868	7.43 [3.36-16.41]	7.00e-07
21:28438192	A/C	0.0116	0.7272	38.92 [9.77-155.08]	2.09e-07
21:28438550	C/T	0.0108	0.7448	44.36 [10.74-183.25]	1.61e-07

RA/EA=Reference Allele/Effect Allele
EAF=Effect Allele Frequency
RSQ=Imputation quality metric from Minimac; the estimated squared correlation between the imputed and true genotype

Supplementary Table 8: Suggestive asthma associations in Puerto Ricans (GALA II). Associations with p-values<10^{-6} are shown.

Chromosome: hg19 position	RA/ EA	EAF	RSQ	OR [95% CI]	P-value
8:19172217	G/C	0.7426	0.9396	1.48 [1.27-1.73]	8.75e-07
17:37912377	C/T	0.3328	0.9804	0.68 [0.59-0.79]	2.65e-07
17:37922259	G/A	0.3236	0.9981	0.67 [0.58-0.77]	6.25e-08
17:37938047	C/T	0.3461	1.0003	0.70 [0.60-0.81]	9.81e-07
17:37939839	C/T	0.3461	0.9985	0.70 [0.60-0.81]	9.79e-07
17:37940808	C/T	0.3386	0.9970	0.69 [0.59-0.79]	4.32e-07
17:37962987	T/A	0.3454	0.9953	0.69 [0.60-0.80]	7.30e-07
17:37970149	A/G	0.3452	1.0002	0.69 [0.60-0.80]	7.35e-07
17:38080912	G/A	0.6613	0.9888	1.44 [1.25-1.67]	8.99e-07

RA/EA=Reference Allele/Effect Allele
EAF=Effect Allele Frequency
RSQ=Imputation quality metric from Minimac; the estimated squared correlation between the imputed and true genotype

Supplementary Table 9: Selected CAAPA meta-analysis results. Associations with p-value<10^{-6} in the CAAPA meta-analysis, lead SNPs from the TAGC meta-analysis, the 2 African-ancestry asthma associated variants identified by EVE and 1 African-ancestry asthma associated variant identified by eMERGE are listed in this table.
RA/EA=Reference Allele/Effect Allele
EAF=Effect Allele Frequency
SE=Standard Error
Effect Direction: ? denotes a missing value. Order of CAAPA studies: BASS, BIAS, CAG, PGCA, SAPPHIRE, HONDAS, JHS(1), JHS(2), JAAS, GRAAD(2), BAGS, GRAAD(1), ProAR, GALA II, SAGE II, HUDS, SARP
Passoc: Association P-value
Panc_het: Ancestry heterogeneity P-value
Presid_het: Residual heterogeneity P-value

Chromosome: hg19 position	RA/EA	EAF	Effect direction	$ß_0$	SE_0	$ß_1$	SE_1	P_{assoc}	P_{anc_het}	P_{resid_het}
1:158932555	C/T	0.779	-++-+-+-+-+++++++-	0.0762	0.0602	1.059	1.440	6.38E-03	4.19E-01	2.56E-01
1:158932907	G/A	0.779	-++-+-+-+-+++++++-	0.0758	0.0602	1.073	1.440	6.25E-03	4.13E-01	2.55E-01
2:102957716	C/T	0.330	--++++--+++++++++-	0.0416	0.0396	0.656	0.668	2.86E-02	3.44E-01	5.31E-01
5:110404999	A/C	0.116	-+++-+--++-+++++-+	0.0532	0.0645	0.850	0.824	5.03E-02	3.62E-01	6.98E-01
5:131995964	A/G	0.792	+-+-+++++++------+	-0.0250	0.0392	-0.530	0.642	2.67E-01	5.01E-01	8.23E-01
5:141492419	C/A	0.759	-+++-+--++-++++--+	0.0034	0.0443	1.149	0.706	5.94E-02	1.19E-01	5.46E-01
6:28712247	A/G	0.359	--++-+--++--+++--	-0.0157	0.0404	0.965	0.738	3.21E-01	2.02E-01	5.05E-01
6:32604372	G/A	0.552	-+-++++++-++-++-++	0.0522	0.0479	0.410	0.832	2.98E-02	5.49E-01	1.03E-01
6:90986686	G/A	0.145	+----++-----+++--	-0.1150	0.0478	1.091	0.674	1.28E-01	1.75E-01	7.86E-01
6:145053377	T/C	0.117	+--------------+-	-0.3275	0.0813	0.588	1.260	8.33E-07	6.54E-01	5.34E-01
8:2548759	G/A	0.118	++++++-+-+++++++++	0.1455	0.0517	1.683	0.909	9.67E-07	1.27E-01	8.07E-01
8:2548821	T/C	0.129	++++++-+-+++++++++	0.1452	0.0524	1.757	0.937	1.71E-07	9.83E-02	7.04E-01
8:2550131	T/C	0.154	++++++-+-+++++++++	0.1307	0.0517	1.807	0.960	1.26E-07	7.74E-02	5.87E-01
8:2550275	G/A	0.154	++++++-+-+++++++++	0.1307	0.0516	1.810	0.959	1.24E-07	7.71E-02	5.90E-01
8:2550492	C/T	0.197	+++++--+++++++++++	0.1137	0.0354	1.616	0.679	5.10E-07	9.29E-02	9.43E-01
8:2550802	C/T	0.125	+++++--+++++++++++	0.1658	0.0602	1.690	1.079	3.15E-08	1.19E-01	4.58E-01
8:2551365	C/T	0.126	+++++--+++++++++++	0.1581	0.0605	1.813	1.082	4.30E-08	9.49E-02	4.58E-01
8:81278885	G/A	0.574	--+-----+-+---+-+	-0.0423	0.0347	0.117	0.586	3.95E-01	8.63E-01	7.41E-01
8:125491675	G/A	0.015	------?--?-+-----	-0.4993	0.1759	-8.210	4.934	9.95E-07	1.83E-01	8.22E-01

Chromosome: hg19 position	RA/EA	EAF	Effect direction	ß$_0$	SE$_0$	ß$_1$	SE$_1$	P$_{assoc}$	P$_{anc_het}$	P$_{resid_het}$
8:125494270	A/T	0.015	------?--?-+-----	-0.5035	0.1775	-8.162	4.973	8.75E-07	1.85E-01	8.10E-01
8:125494894	G/A	0.015	------?--?-+-----	-0.5040	0.1777	-8.161	4.978	8.57E-07	1.85E-01	8.09E-01
8:125501992	C/T	0.014	------?--?-------	-0.5832	0.1610	-5.893	4.353	2.79E-07	3.03E-01	8.73E-01
8:125531353	C/T	0.013	------?--?------?	-0.6063	0.1554	-4.398	4.107	2.70E-07	4.10E-01	8.50E-01
8:125542823	T/C	0.013	------?--?------?	-0.6142	0.1522	-3.731	3.987	2.85E-07	4.73E-01	8.53E-01
8:125543356	T/C	0.013	------?--?------?	-0.6142	0.1520	-3.698	3.978	2.86E-07	4.76E-01	8.53E-01
8:125546339	A/C	0.013	------?--?------?	-0.6136	0.1509	-3.484	3.937	3.16E-07	4.97E-01	8.53E-01
8:125546613	C/T	0.013	------?--?------?	-0.6134	0.1510	-3.465	3.938	3.16E-07	4.99E-01	8.51E-01
8:125546839	G/A	0.013	------?--?------?	-0.6134	0.1512	-3.438	3.941	3.24E-07	5.02E-01	8.50E-01
8:125551377	A/G	0.012	------?--?------?	-0.6059	0.1537	-3.352	3.990	4.20E-07	5.08E-01	8.26E-01
8:125563159	G/C	0.012	------?+-?-------	-0.6281	0.1631	-2.213	4.197	5.10E-07	6.58E-01	7.60E-01
8:125588848	A/C	0.012	-----+?+-?-------	-0.6372	0.1738	-1.278	4.417	4.88E-07	7.89E-01	6.01E-01
8:125591449	C/T	0.012	-----+?+-?-------	-0.6324	0.1749	-1.342	4.438	5.76E-07	7.79E-01	5.91E-01
8:125594428	A/T	0.012	-----+?+-?-------	-0.6281	0.1756	-1.415	4.450	6.33E-07	7.67E-01	5.83E-01
8:125675028	G/A	0.014	-----+?--?-------	-0.6710	0.1583	1.133	3.975	8.85E-07	8.02E-01	6.85E-01
9:6209697	A/G	0.695	--+-+------------	-0.1350	0.0365	0.299	0.636	1.13E-04	6.83E-01	7.34E-01
9:132502801	G/A	0.092	+++-+-+-+-++++-++	0.1064	0.0761	-0.768	1.700	2.26E-01	6.38E-01	3.66E-01
10:9046645	A/G	0.869	++---+---+++-----	-0.0848	0.0719	-0.181	1.281	8.49E-02	8.67E-01	1.36E-01
11:76301316	C/T	0.340	-++++--++--++-+--	0.1221	0.0576	-1.077	0.954	8.93E-03	1.15E-01	1.53E-02
12:57503775	C/T	0.208	+-++++---+-++++-+	0.0206	0.0609	1.707	0.911	6.87E-04	2.43E-02	1.16E-01
12:57509055	T/C	0.518	+++++++--++++++--	0.0955	0.0450	1.211	0.748	1.20E-07	7.44E-02	2.53E-01
12:57509102	C/T	0.262	+-++++++-++++++--	0.0854	0.0480	1.426	0.743	9.08E-07	4.67E-02	3.76E-01
15:61049569	A/C	0.311	-------------+--+	-0.2238	0.0469	2.004	0.864	2.14E-07	1.10E-02	2.61E-01
15:61068347	G/A	0.298	-+-------+-------	-0.2072	0.0420	1.199	0.791	1.82E-07	1.46E-01	5.39E-01
15:61068704	G/A	0.346	-----------------	-0.1976	0.0339	1.261	0.636	7.09E-07	1.16E-01	8.53E-01
15:61068954	T/C	0.291	-+-------+------+	-0.1938	0.0330	0.844	0.597	3.25E-07	2.84E-01	8.96E-01
15:67449660	A/G	0.420	+++--++--++++++--	0.0271	0.0420	0.388	0.718	2.31E-01	5.67E-01	3.28E-01
16:11199980	G/A	0.072	+-+-+++--?+--+---	-0.1011	0.0708	1.653	0.934	2.81E-01	1.12E-01	6.63E-01
17:37876835	A/G	0.584	--+----++-+---++-	-0.0809	0.0586	-0.131	0.995	1.03E-02	8.54E-01	1.45E-02
17:37886986	C/A	0.105	------+--+------+	-0.1932	0.0529	-1.240	0.692	5.30E-08	1.91E-01	9.24E-01
17:37908867	C/A	0.108	------+--+------+	-0.1946	0.0515	-1.113	0.679	1.63E-08	2.12E-01	8.92E-01
17:37912377	C/T	0.224	--+-------------+	-0.1032	0.0470	-2.139	0.733	4.29E-11	4.06E-03	4.85E-01
17:37922259	G/A	0.194	----+-----------+	-0.1217	0.0527	-2.118	0.793	4.38E-12	5.08E-03	3.49E-01
17:37938047	C/T	0.230	----------------+	-0.1078	0.0493	-1.897	0.765	1.97E-10	9.65E-03	3.60E-01
17:37939839	C/T	0.230	----------------+	-0.1079	0.0492	-1.899	0.765	1.98E-10	9.66E-03	3.63E-01
17:37940808	C/T	0.227	----------------+	-0.1039	0.0499	-2.039	0.776	8.70E-11	5.68E-03	3.43E-01
17:37962987	T/A	0.230	----------------+	-0.1082	0.0485	-1.935	0.754	1.35E-10	8.63E-03	4.01E-01
17:37970149	A/G	0.229	----------------+	-0.1080	0.0480	-1.926	0.748	1.38E-10	8.76E-03	4.13E-01
17:37992281	C/T	0.108	------+--+------+	-0.1747	0.0554	-1.307	0.738	4.70E-08	1.47E-01	8.16E-01
17:38023745	A/G	0.239	----------------+	-0.0773	0.0457	-2.174	0.721	1.38E-09	2.97E-03	4.84E-01
17:38024626	C/T	0.184	---------+------+	-0.1096	0.0456	-1.912	0.679	8.58E-10	1.20E-02	6.83E-01
17:38025208	T/C	0.184	---------+------+	-0.1100	0.0459	-1.915	0.684	7.65E-10	1.18E-02	6.71E-01
17:38025417	G/A	0.107	+----+---+------+	-0.1801	0.0545	-1.217	0.738	1.95E-08	1.68E-01	7.88E-01
17:38026035	G/A	0.169	----------------+	-0.1613	0.0472	-1.420	0.691	1.52E-10	7.13E-02	7.11E-01
17:38031674	C/T	0.180	-----------+----+	-0.1226	0.0493	-1.799	0.727	4.47E-10	1.88E-02	5.63E-01
17:38031857	T/G	0.184	---------+------+	-0.1104	0.0457	-1.915	0.682	7.08E-10	1.18E-02	6.76E-01
17:38032460	C/T	0.184	---------+------+	-0.1103	0.0457	-1.914	0.682	7.21E-10	1.19E-02	6.76E-01
17:38032680	T/C	0.175	----------------+	-0.1489	0.0485	-1.665	0.710	2.40E-11	3.01E-02	6.14E-01
17:38035624	C/T	0.179	-----------+----+	-0.1241	0.0492	-1.779	0.724	4.42E-10	2.02E-02	5.71E-01
17:38038179	C/T	0.179	-----------+----+	-0.1232	0.0491	-1.784	0.722	4.80E-10	1.98E-02	5.75E-01
17:38040119	T/C	0.175	----------------+	-0.1481	0.0484	-1.670	0.708	2.53E-11	2.94E-02	6.20E-01
17:38043649	C/T	0.179	-----------+----+	-0.1237	0.0490	-1.779	0.721	4.65E-10	2.01E-02	5.79E-01
17:38050094	G/A	0.247	----------+----++	-0.1041	0.0527	-1.517	0.848	2.22E-08	4.04E-02	1.84E-01
17:38056116	T/C	0.259	----------+----++	-0.1108	0.0507	-1.488	0.831	4.30E-09	3.99E-02	1.83E-01
17:38057189	G/A	0.174	----------------+	-0.1505	0.0482	-1.626	0.703	2.98E-11	3.44E-02	6.37E-01

Chromosome: hg19 position	RA/EA	EAF	Effect direction	ß$_0$	SE$_0$	ß$_1$	SE$_1$	P_{assoc}	P_{anc_het}	P_{resid_het}
17:38057841	G/C	0.182	----------------+	-0.1347	0.0406	-0.993	0.654	6.14E-07	2.22E-01	8.38E-01
17:38061439	T/C	0.178	---------+------+	-0.1321	0.0467	-1.613	0.695	7.49E-10	3.57E-02	6.59E-01
17:38062196	G/A	0.178	---------+------+	-0.1321	0.0467	-1.612	0.694	7.47E-10	3.58E-02	6.59E-01
17:38062217	C/T	0.209	----+----+-+----+	-0.1056	0.0503	-1.520	0.766	3.26E-08	3.97E-02	3.73E-01
17:38062944	A/C	0.199	----+----+-+----+	-0.1069	0.0518	-1.721	0.795	5.90E-09	2.27E-02	3.40E-01
17:38062976	G/A	0.178	---------+------+	-0.1324	0.0466	-1.609	0.692	7.54E-10	3.63E-02	6.66E-01
17:38063381	C/T	0.209	----+----+-+----+	-0.1053	0.0503	-1.533	0.767	3.10E-08	3.83E-02	3.74E-01
17:38063738	C/T	0.209	----+----+-+----+	-0.1053	0.0502	-1.535	0.765	3.09E-08	3.81E-02	3.79E-01
17:38063929	T/C	0.209	----+----+-+----+	-0.1052	0.0501	-1.536	0.764	3.09E-08	3.80E-02	3.84E-01
17:38064405	C/T	0.173	----------------+	-0.1556	0.0483	-1.486	0.710	7.81E-11	5.50E-02	6.31E-01
17:38064469	T/C	0.173	----------------+	-0.1548	0.0483	-1.491	0.710	8.37E-11	5.41E-02	6.30E-01
17:38064876	G/A	0.173	----------------+	-0.1546	0.0483	-1.492	0.710	8.62E-11	5.40E-02	6.31E-01
17:38067020	C/T	0.191	----+----+------+	-0.1085	0.0473	-1.782	0.717	3.00E-09	1.85E-02	5.67E-01
17:38068043	T/C	0.775	++++-++++---++++-	0.0810	0.0487	1.783	0.751	5.35E-08	1.39E-02	3.77E-01
17:38069076	G/A	0.768	++++-++++---+++++	0.0734	0.0446	1.875	0.692	5.34E-08	9.23E-03	5.37E-01
17:38069274	C/T	0.768	++++-++++---+++++	0.0740	0.0445	1.871	0.690	5.09E-08	9.36E-03	5.43E-01
17:38069809	C/T	0.768	++++-++++---+++++	0.0772	0.0438	1.845	0.679	3.83E-08	1.03E-02	5.73E-01
17:38069949	C/T	0.768	++++-++++---+++++	0.0775	0.0437	1.843	0.678	3.73E-08	1.04E-02	5.76E-01
17:38070789	C/T	0.781	++-+-++++---+++++	0.0823	0.0549	1.673	0.836	1.89E-07	2.13E-02	1.78E-01
17:38072173	G/T	0.781	++-+-++++---+++++	0.0825	0.0550	1.671	0.836	1.87E-07	2.14E-02	1.77E-01
17:38072245	C/G	0.899	+++++++++-+-++++-	0.1913	0.0514	0.868	0.664	3.00E-07	3.37E-01	9.20E-01
17:38072247	T/G	0.781	++-+-++++---+++++	0.0823	0.0550	1.667	0.836	2.03E-07	2.18E-02	1.76E-01
17:38072402	C/T	0.781	++-+-++++---+++++	0.0826	0.0550	1.664	0.837	2.01E-07	2.20E-02	1.76E-01
17:38072727	C/T	0.812	++-+-+++++-++++++	0.1076	0.0547	1.844	0.812	2.17E-09	1.49E-02	3.04E-01
17:38073968	G/C	0.824	+++++++++++-++++-	0.1537	0.0469	1.634	0.687	1.36E-11	3.34E-02	6.78E-01
17:38075016	T/C	0.625	++-++++++---+++++	0.0571	0.0336	1.657	0.567	7.55E-07	1.46E-02	7.88E-01
17:38080912	G/A	0.793	++-+-++++-+++++++	0.0855	0.0406	2.257	0.617	3.08E-10	2.50E-03	8.04E-01
17:38089717	A/G	0.793	++-++++++++++++++	0.0901	0.0389	1.979	0.592	1.23E-08	1.00E-02	8.82E-01
17:47461433	G/A	0.115	-+++-+-+-++++--++	0.1007	0.0910	-1.822	1.135	1.16E-01	3.98E-02	5.56E-02
18:42607527	T/G	0.021	+??++++++?++---++	0.9724	0.1711	-17.766	3.401	7.95E-07	2.41E-06	6.36E-01
19:54192692	T/G	0.039	---------?-------	-0.5441	0.1118	3.870	2.526	5.41E-07	1.61E-01	6.28E-01

Supplementary Table 10: Taqman genotyping results for low frequency variants in CAAPA meta-analysis with p-values < 10^{-6}. These SNPs were genotyped in subjects from BAGS and JAAS. See Supplementary Note 11. HWE=Hardy-Weinberg equilibrium. MAF=minor allele frequency

Chromosome:hg19 position	rsID	A1	A2	Genotypes (TT/CT/CC)	HWE p-value	MAF	Call rate
**BAGS Founders**							
8:125531353	rs114647118	T	C	0/6/279	1	0.011	0.968
18:42607527	rs73952947	T	G	5/14/266	3E-05	0.042	0.961
19:54192692	rs73595000	T	G	0/26/248	1	0.047	0.937
**JAAS Controls**							
8:125531353	rs114647118	T	C	0/11/376	1	0.014	0.878
18:42607527	rs73952947	T	G	2/18/359	3E-02	0.029	0.859
19:54192692	rs73595000	T	G	0/56/315	2E-01	0.075	0.841
**JAAS Asthmatics**							
8:125531353	rs114647118	T	C	0/0/99	1	0.000	0.728
18:42607527	rs73952947	T	G	2/2/113	8E-04	0.026	0.860
19:54192692	rs73595000	T	G	0/14/82	1	0.073	0.706
		**% Concordance**					
**BAGS**							
8:125531353	rs114647118	99.3					
18:42607527	rs73952947	94.9					
19:54192692	rs73595000	91.6					
**JAAS**							
8:125531353	rs114647118	99.2					
18:42607527	rs73952947	95.3					
19:54192692	rs73595000	85.4					

Supplementary Table 11: Comparison of case and control counts for rs114647118 in BAGS and JAAS. This SNP is a low frequency variant associated with protection against asthma in the CAAPA meta-analysis. Taqman genotyping was performed to verify imputation quality. See Supplementary Table S10 and Supplementary Note 11.

Study	Total number controls	Total number cases	Control imputation mismatch	Case imputation mismatch	Number Taqman control heterozygotes	Number Taqman case heterozygotes
BAGS	338	282	2 CC genotypes imputed as CT	2 CC genotypes imputed as CT	15	3
JAAS	507	167	3 CT genotypes imputed as CC	None	11	0

Supplementary Table 12: Previous GWAS results not replicated in CAAPA. Genome-wide significant associations reported by previous asthma GWAS (TAGC, EVE, and eMERGE) that did not replicate in CAAPA are listed. Because some of the CAAPA studies were included in the TAGC asthma meta-analysis, and in order to contrast African and European asthma susceptibility loci, the table summarizes the associations in TAGC Europeans only (statistics from the TAGC random effects analysis, reported for the CAAPA and TAGC results).

						CAAPA			Prior GWAS		
Locus	Descr.	rsID	hg19 position	Genes	RA/EA	EAF	OR [95% CI]	P	EAF	OR [95% CI]	P
					TAGC						
5q31.3	new	rs7705042	141,492,419	*NDFIP1, GNDPA1,SPRY4*	C/A	0.76	1.06 [0.99-1.13]	0.06	0.63	1.08 [1.05-1.11]	$1.6x10^{-6}$
6p21	anc	rs2855812	31,472,720	*MICB,HCP5, MCCD1*	G/T				0.23	1.10 [1.06-1.13]	$1.7x10^{-8}$
6q15	new	rs2325291	90,986,686	*BACH2,GJA10, MAP3K7*	G/A	0.15	0.94 [0.88-1.02]	0.13	0.33	0.91 [0.89-0.93]	$8.6x10^{-13}$
8q21	a+h	rs12543811	81,278,885	*TPD52,ZBTB10*	G/A	0.57	0.96 [0.91-1.02]	0.40	0.66	0.93 [0.91-0.95]	$3.4x10^{-8}$
10p14	anc	rs2589561	9,046,645	*GATA3,CELF2*	A/G	0.87	0.91 [0.84-0.99]	0.09	0.82	0.90 [0.87-0.94]	$1.4x10^{-8}$
16p13	a+h	rs17806299	11,199,980	*CLEC16A,DEXI, SOCS1*	G/A	0.07	0.99 [0.90-1.10]	0.28	0.20	0.90 [0.88-0.93]	$2.1x10^{-10}$
17q21.33	new	rs17637472	47,461,433	*ZNF652,PHB*	G/A	0.12	0.99 [0.90-1.08]	0.12	0.39	1.08 [1.05-1.11]	$3.3x10^{-9}$
					EVE						
1q23	new	rs1101999	158,932,555	*PYHIN1*	C/T	0.78	1.12 [1.04-1.20]	$6.4x10^{-3}$	0.79	*	$4.0x10^{-9}$
				CAAPA studies in EVE		0.78	1.17 [1.09-1.26]	$8.1x10^{-3}$			
				CAAPA studies not in EVE		0.79	1.07 [0.99-1.14]	0.45			
					eMERGE						
9q34	new	rs11788591	132,502,801	*PTGES*	G/A	0.09	1.08 [0.99-1.19]	0.23		**	$4.5x10^{-8}$

* No effect size available, but the major allele is more frequent in EVE African cases compared to controls, consistent with the direction of effect in CAAPA
** Effect size and effect allele information not available
new: asthma GWAS result not reported prior to the corresponding GWAS
a+h: asthma+hayfever GWAS result reported prior to the TAGC GWAS
anc: asthma GWAS result reported as ancestry specific prior to the TAGC GWAS
RA=reference allele
EA=effect allele
EAF=effect allele frequency; Thousand Genomes Project phase III European allele frequencies are reported as the estimated EAF for TAGC Europeans
P=P asthma association

Supplementary Table 13: Replication of significant associations in TAGC Europeans in the CAAPA meta-analysis. The TAGC European summary statistics were merged with the CAAPA MR-MEGA meta-analysis results, and associations (fixed effects p-values) in TAGC that pass a Bonferroni correction for the number of merged SNPs were selected for replication in CAAPA (810 SNPs). There were 52 associations in CAAPA that pass a Bonferroni correction for 810 tests, and they are listed in this table.

Chromosome	hg19 position	TAGC European fixed effects p-value	CAAPA p-value
9	6144333	1.21E-08	1.24E-05
9	6197392	9.52E-27	1.78E-05
15	61042867	9.80E-09	2.19E-06
15	61047144	5.04E-09	3.53E-06
15	61048200	5.13E-09	4.03E-06
15	61048213	5.08E-09	1.04E-06
15	61048578	4.87E-09	4.03E-06
15	61055411	6.39E-09	7.10E-06
15	61056035	4.03E-09	1.22E-06
15	61065553	1.86E-09	1.33E-06
15	61066516	2.16E-09	1.49E-06
15	61068347	1.49E-10	1.82E-07
15	61068954	1.47E-09	3.25E-07
17	37770005	9.56E-14	1.26E-05
17	37784990	4.27E-14	1.25E-05
17	37790371	1.28E-12	9.39E-06
17	37790939	4.64E-10	9.49E-06
17	37829604	1.43E-24	2.23E-05
17	37921742	1.12E-36	1.87E-05
17	37922259	2.16E-32	4.38E-12
17	37938047	3.99E-34	1.97E-10
17	37970149	4.16E-34	1.38E-10
17	37976469	1.18E-40	6.36E-06
17	38023745	2.15E-27	1.38E-09
17	38024626	6.18E-32	8.58E-10
17	38025208	1.01E-33	7.65E-10
17	38025417	1.12E-19	1.95E-08
17	38028634	1.39E-29	4.86E-05
17	38031714	2.00E-26	1.10E-05
17	38033277	8.20E-34	1.13E-05
17	38035116	6.85E-34	4.64E-06
17	38040119	5.47E-34	2.53E-11
17	38040763	5.24E-34	4.59E-06
17	38043343	1.87E-40	5.81E-05
17	38049589	2.16E-37	3.33E-05
17	38057197	2.92E-36	1.08E-05
17	38062196	3.18E-36	7.47E-10
17	38062217	1.00E-42	3.26E-08
17	38062976	7.13E-29	7.54E-10
17	38064405	4.21E-36	7.81E-11
17	38066267	6.29E-42	3.39E-05
17	38067020	1.25E-35	3.00E-09
17	38068043	8.01E-41	5.35E-08
17	38069949	2.51E-42	3.73E-08
17	38074031	1.38E-38	1.07E-05
17	38074518	2.81E-42	2.06E-05
17	38075426	2.45E-42	2.12E-05
17	38080865	2.09E-40	1.66E-05
17	38080912	2.44E-34	3.08E-10
17	38085722	6.87E-11	5.21E-05
17	38088417	1.39E-39	1.66E-06
17	38114598	2.27E-35	5.93E-05

Supplementary Table 14: Comparison of the association between rs11020000 and asthma in studies included in the EVE and CAAPA meta-analyses. The p-value for this SNP was $3.6x10^{-7}$ in the EVE discovery meta-analysis, and $8.1x10^{-3}$ in the CAAPA meta-analysis. Summary statistics for this SNP was extracted from the EVE supplementary material, and corresponding statistics were extracted in CAAPA. The reduced association strength in CAAPA can be explained by a smaller sample size for two of the studies that have a large effect size (BAGS and GRAAD), and the larger sample size but small effect size of SAPPHIRE in CAAPA.

	EVE				CAAPA				
	Nr cases	Frequency* Control	Frequency Cases	Rsq**	Nr cases	Frequency Control	Frequency Cases	Rsq	OR [95%CI]
**BAGS**	355	0.724	0.776	1.00	282	0.729	0.778	0.75	1.28 [0.94-1.75]
**GRAAD(1)**	464	0.708	0.774	1.00	396	0.719	0.780	0.75	1.45 [1.11-1.90]
**SAPPHIRE**	149	0.735	0.785	0.42	1325			0.71	1.05 [0.86-1.27]
**CAG**					114	0.740	0.763	0.83	1.09 [0.69-1.70]
**SARP**					302	0.767	0.733	0.78	0.87 [0.48-1.56]
**CAG+SARP**	644	0.723	0.757	0.97					

* Frequency of the T allele

** Estimated squared correlation between the imputed and true allelic dose

.

Supplementary Table 15: Sensitivity analysis of age of asthma onset in high African ancestry studies and associations with asthma in chr17q12-21. 17 putatively causal SNPs reported in the Stein et al. review (doi 10.1016/j.jaci.2017.12.974), as well as an additional 5 SNPs from the CAAPA meta-analysis, with p-values < 10^{-6} and r^2 < 0.8 with all 17 SNPs in TGP European and African populations, were extracted. Logistic regression was used to test for association between imputed allelic dose and asthma, when including all asthmatics (All), and when including only childhood onset asthmatics. The originating CAAPA data set was included as covariate in the models. Only studies with high African ancestry (mean African ancestry > 75%), that had age of onset information available or that were pediatric studies were included in the analysis (JHS, BAGS, GRAAD, CAG, HUFS, JAAS, SAGE II, SARP, BASS 4,717 controls, 2,910 adult and childhood onset asthmatics, 1,876 childhood onset asthmatics).

		All		Childhood onset	
**SNP**	**RA/EA**	**OR [95% CI]**	**P**	**OR [95% CI]**	**P**
17:37830900	A/G	0.97 [0.90-1.04]	3.58E-01	1.02 [0.93-1.11]	7.32E-01
17:37843681	C/T	0.92 [0.84-1.01]	8.49E-02	0.96 [0.87-1.07]	4.95E-01
17:37876835	A/G	0.99 [0.91-1.07]	7.27E-01	1.04 [0.95-1.14]	4.02E-01
17:37886986	C/A	0.75 [0.63-0.88]	4.30E-04	0.73 [0.60-0.88]	1.08E-03
17:37908867	C/A	0.78 [0.67-0.90]	1.07E-03	0.75 [0.63-0.90]	1.60E-03
17:37922259	G/A	0.87 [0.79-0.96]	7.92E-03	0.86 [0.76-0.97]	1.45E-02
17:37976469	C/T	0.95 [0.89-1.03]	2.14E-01	0.94 [0.87-1.03]	1.88E-01
17:37992281	C/T	0.77 [0.67-0.89]	5.74E-04	0.74 [0.62-0.88]	7.57E-04
17:38029120	C/G	0.95 [0.89-1.03]	1.98E-01	0.92 [0.85-1.01]	6.92E-02
17:38057197	G/A	0.95 [0.88-1.02]	1.82E-01	0.93 [0.85-1.02]	1.15E-01
17:38057841	G/C	0.86 [0.78-0.95]	2.93E-03	0.85 [0.76-0.95]	5.95E-03
17:38062196	G/A	0.87 [0.79-0.97]	1.10E-02	0.84 [0.74-0.95]	6.25E-03
17:38064405	C/T	0.84 [0.76-0.94]	2.19E-03	0.81 [0.71-0.92]	1.50E-03
17:38064469	T/C	0.85 [0.76-0.94]	2.34E-03	0.81 [0.71-0.92]	1.59E-03
17:38066240	T/C	0.96 [0.89-1.03]	2.13E-01	0.93 [0.85-1.01]	8.40E-02
17:38069949	C/T	1.11 [1.01-1.21]	3.20E-02	1.13 [1.02-1.26]	2.47E-02
17:38072245	C/G	1.30 [1.11-1.52]	1.19E-03	1.33 [1.10-1.60]	3.11E-03
17:38080865	A/G	1.05 [0.98-1.14]	1.89E-01	1.08 [0.99-1.18]	9.55E-02
17:38080912	G/A	1.12 [1.01-1.24]	2.54E-02	1.17 [1.04-1.32]	8.25E-03
17:38082807	T/C	1.05 [0.97-1.13]	2.36E-01	1.05 [0.96-1.15]	2.64E-01
17:38121993	G/A	1.06 [0.98-1.16]	1.31E-01	1.09 [0.99-1.20]	7.23E-02
17:38128648	C/T	1.04 [0.96-1.12]	3.31E-01	1.07 [0.98-1.17]	1.52E-01

Supplementary Table 16: Single SNP and haplotype association analysis of rs12936231 and rs4065275. In Yoruban lymphoblastoid cell lines, the rs12936231-C and rs4065275-G alleles are associated with high expression of ORMDL3, but the rs4065275-G allele is not associated with expression of ORMDL3. The most probable imputed genotypes were used for single SNP association tests. The R haplostats package was used to infer the most likely haplotypes from the most probable genotypes, and to test for association between haplotypes and asthma. Additive allelic logistic regression models were used for all association tests, and the originating CAAPA data set was included as covariate.

Unrelated asthmatics with 2 African haplotypes (1,717 cases + 2,598 controls)						
**Single SNP**						
**MARKERID**	**POS**	**REF**	**EFF**	**EAF**	**OR [95% CI]**	**P**
rs12936231	38029120	G	C	0.45	1.07 [0.97-1.18]	1.80E-01
rs4065275	38080865	A	G	0.67	1.04 [0.94-1.15]	4.80E-01
**Haplotype**						
rs12936231-rs4065275	38029120+38080865	G-A	C-A	0.04	0.98 [0.73-1.32]	9.10E-01
		G-A	C-G	0.41	1.07 [0.95-1.20]	2.57E-01
		G-A	G-G	0.26	1.07 [0.94-1.22]	8.33E-01
**Unrelated asthmatics with 2 African haplotypes - childhood onset asthmatics (age of asthma onset < 16 years) only (1,118 cases + 2,598 controls)**						
**Single SNP**						
**MARKERID**	**POS**	**REF**	**EFF**	**EAF**	**OR [95% CI]**	**P**
rs12936231	38029120	G	C	0.45	1.09 [0.97-1.22]	1.37E-01
rs4065275	38080865	A	G	0.67	1.07 [0.95-1.20]	2.96E-01
**Haplotype**						
rs12936231-rs4065275	38029120+38080865	G-A	C-A	0.04	1.01 [0.72-1.42]	9.51E-01
		G-A	C-G	0.41	1.10 [0.96-1.26]	1.52E-01
		G-A	G-G	0.26	1.01 [0.86-1.18]	8.89E-01

REF=Reference allele
EFF=Effect allele
EAF=Effect allele frequency

Supplementary Table 17: Sensitivity analysis of age of asthma onset and associations with asthma in 8p23. Logistic regression was used to test for association between imputed allelic dose of SNP rs13277810 and asthma, when including all asthmatics (All), and when including only childhood onset asthmatics. The originating CAAPA data set was included as covariate in the model. Only studies that had age of onset information available or that were pediatric studies were included in the analysis (JHS, BAGS, GRAAD, CAG, HUFS, JAAS, SAGE II, SARP, BASS, GALA II; 5,570 controls, 3,811 adult and childhood onset asthmatics, 2,591 childhood onset asthmatics).

		All		Childhood onset	
**SNP**	**RA/EA**	**OR [95% CI]**	**P**	**OR [95% CI]**	**P**
8:2550802	C/T	1.32 [1.19-1.46]	5.97E-08	1.28 [1.14-1.45]	3.75E-05

Supplementary Table 18: LD Block comparison between the loci reported by TAGC and the CAAPA meta-analysis. SNPs within the same linkage disequilibrium (LD) block and in high LD with (*r2* > 0.8) the TAGC lead SNPs in Europeans from the 1000 Genomes Project (TGP) were selected for the LD block comparison (because the European population is the largest population in TAGC, and because some of the CAAPA studies were included in TAGC, the LD block comparison was done considering European LD only). P-values <0.05 are highlighted.

RA=Reference Allele, EA=Affect Allele, EAF=Effect Allele Frequency

CAAPA Meta-regression P-values: P_{assoc}=P-value association, P_{anc}=P-value ancestry heterogeneity, P_{resid}=P-value residual heterogeneity

rs1420101 2q12

	hg19 position	r^2	RA/ EA	TGP EAF	CAAPA EAF	P_{assoc}	P_{anc}	P_{resid}
rs12712142	102960584	0.84	C/A	0.3748	0.3409	1.78E-02	3.99E-01	6.00E-01
rs6543119	102963072	0.85	A/T	0.3777	0.3409	1.80E-02	4.12E-01	6.20E-01
rs13017455	102964742	0.85	C/T	0.3777	0.3414	1.77E-02	4.16E-01	6.31E-01
rs11123923	102967844	0.84	C/A	0.3767	0.1959	9.93E-02	2.60E-01	6.97E-01

rs10455025 5q22.1

	hg19 position	r^2	RA/ EA	TGP EAF	CAAPA EAF	P_{assoc}	P_{anc}	P_{resid}
rs1898671	110408002	0.98	C/T	0.3628	0.1302	2.54E-01	3.47E-01	3.02E-01

rs7705042 5q31.3

	hg19 position	r^2	RA/ EA	TGP EAF	CAAPA EAF	P_{assoc}	P_{anc}	P_{resid}
rs6580223	141489027	1	T/G	0.3797	0.2356	7.77E-02	1.04E-01	5.28E-01
rs6580224	141489306	1	G/A	0.3797	0.2282	6.71E-02	1.28E-01	5.71E-01
rs6580225	141489381	1	C/G	0.3797	0.2282	6.72E-02	1.29E-01	5.69E-01
rs12653848	141489853	1	T/C	0.3797	0.2361	7.90E-02	1.09E-01	5.11E-01
rs12653866	141489921	1	T/C	0.3797	0.2282	6.83E-02	1.31E-01	5.56E-01
rs12656877	141490091	1	C/T	0.3797	0.2282	6.72E-02	1.31E-01	5.53E-01
rs4912622	141490587	1	A/G	0.3797	0.2361	7.58E-02	1.08E-01	5.00E-01
rs7700687	141491985	1	T/C	0.3807	0.2371	7.06E-02	1.01E-01	5.00E-01
rs4912804	141493614	1	C/T	0.3797	0.237	6.50E-02	9.44E-02	5.01E-01
rs4912805	141493685	1	G/A	0.3797	0.229	5.64E-02	1.14E-01	5.46E-01
rs13184323	141494719	1	G/T	0.3797	0.2282	5.39E-02	1.10E-01	5.46E-01
rs10068717	141494934	1	T/C	0.3797	0.2361	6.10E-02	8.94E-02	5.02E-01
rs10079513	141495092	1	C/T	0.3797	0.2278	5.24E-02	1.08E-01	5.53E-01
rs13188700	141495139	1	C/T	0.3797	0.2526	8.34E-02	1.11E-01	3.22E-01
rs10875596	141495715	1	C/T	0.3797	0.2288	5.19E-02	1.06E-01	5.45E-01
rs12515668	141496090	1	T/G	0.3797	0.2366	5.51E-02	8.45E-02	5.13E-01
rs7712237	141496464	1	C/T	0.3797	0.2361	5.55E-02	8.43E-02	5.06E-01
rs9324866	141497650	1	G/A	0.3797	0.2367	5.46E-02	8.28E-02	5.08E-01
rs1036209	141498632	1	A/T	0.3797	0.2367	5.39E-02	8.19E-02	5.09E-01
rs1036207	141499041	1	G/A	0.3797	0.2367	5.37E-02	8.16E-02	5.10E-01
rs11749731	141500436	1	C/A	0.3797	0.2367	5.26E-02	7.90E-02	5.12E-01
rs11750521	141500704	1	C/T	0.3797	0.2333	5.44E-02	6.67E-02	3.49E-01
rs1835966	141501508	1	T/C	0.3797	0.2254	4.92E-02	7.90E-02	4.10E-01
rs1835965	141501771	1	A/G	0.3797	0.2333	5.43E-02	6.62E-02	3.47E-01
rs10463348	141502035	1	T/G	0.3797	0.2333	5.44E-02	6.62E-02	3.46E-01

rs20541 5q31

	hg19 position	r^2	RA/ EA	TGP EAF	CAAPA EAF	P_{assoc}	P_{anc}	P_{resid}
rs1295686	131995843	0.96	C/T	0.2137	0.6308	2.17E-03	4.75E-01	6.71E-01

rs1295685	131996445	0.98	G/A	0.2097	0.08494	7.35E-02	9.77E-01	7.22E-01
rs848	131996500	0.96	C/A	0.2127	0.4904	8.11E-02	4.65E-01	2.42E-01
rs847	131996669	0.98	C/T	0.2107	0.1514	1.50E-01	4.36E-01	8.06E-01

rs12335781 6p22

No SNPs met LD inclusion criteria

rs2855812 6p21

SNP not present in TGP

rs2325291 6q15

	hg19 position	r^2	RA/ EA	TGP EAF	CAAPA EAF	P_{assoc}	P_{anc}	P_{resid}
rs62408235	90978383	1	T/C	0.3618	0.1076	2.50E-02	3.90E-02	6.14E-01
rs62408236	90980148	1	C/T	0.3638	0.1761	3.86E-01	5.61E-01	6.98E-01
rs62408237	90980345	1	C/G	0.3638	0.1829	6.09E-01	7.57E-01	6.63E-01
rs55771973	90981319	1	T/G	0.3628	0.1274	1.42E-01	1.90E-01	7.90E-01
rs45553631	90981657	1	C/T	0.3638	0.1761	3.78E-01	5.48E-01	6.98E-01
rs969577	90982387	0.99	T/C	0.3628	0.1076	2.37E-02	3.57E-02	6.17E-01
rs905671	90983850	1	A/T	0.3638	0.1772	3.70E-01	5.29E-01	6.96E-01
rs943689	90984035	1	C/T	0.3638	0.1772	3.69E-01	5.27E-01	6.96E-01
rs58521088	90985198	1	A/T	0.3638	0.184	5.86E-01	7.17E-01	6.59E-01
rs6454805	90986353	1	A/G	0.3638	0.2152	3.68E-01	4.39E-01	7.84E-01
rs6899623	90986559	1	A/G	0.3638	0.2152	3.67E-01	4.39E-01	7.84E-01
rs2325292	90986749	0.99	T/C	0.3648	0.1846	5.71E-01	7.09E-01	6.59E-01
rs2134814	90987512	1	C/G	0.3638	0.1523	4.76E-01	5.59E-01	8.45E-01

rs12543811 8q21.13

	hg19 position	r^2	RA/ EA	TGP EAF	CAAPA EAF	P_{assoc}	P_{anc}	P_{resid}
rs6991991	81271432	0.97	C/T	0.3588	0.5289	2.63E-01	9.24E-01	5.70E-01
rs6996407	81271836	0.9	C/T	0.3787	0.7724	1.30E-01	3.17E-01	7.94E-01
rs13282260	81273135	0.97	A/T	0.3588	0.5284	2.65E-01	9.28E-01	5.63E-01
rs11994858	81273210	0.98	G/A	0.3519	0.4252	3.14E-01	8.72E-01	7.51E-01
rs6987042	81273883	0.9	G/A	0.3767	0.6557	8.15E-02	4.36E-01	6.35E-01
rs2202749	81274338	0.98	G/A	0.3519	0.4251	3.22E-01	8.68E-01	7.52E-01
rs11783496	81275652	0.98	C/T	0.3579	0.5278	2.76E-01	9.29E-01	5.56E-01
rs11786685	81275835	0.98	G/A	0.3579	0.5278	2.77E-01	9.29E-01	5.56E-01
rs11786704	81275860	0.9	C/A	0.3777	0.6897	7.86E-02	4.27E-01	7.55E-01
rs13275449	81276113	0.98	A/G	0.3499	0.4253	3.28E-01	8.64E-01	7.51E-01
rs4739736	81277369	0.98	C/A	0.3579	0.5277	2.82E-01	9.28E-01	5.47E-01
rs13272328	81280496	0.98	C/T	0.3569	0.5272	2.63E-01	9.67E-01	4.89E-01
rs13270496	81280666	0.9	C/A	0.3777	0.7356	7.04E-02	2.95E-01	8.22E-01
rs7826521	81280778	0.9	G/A	0.3748	0.6546	6.92E-02	3.79E-01	5.83E-01
rs6473225	81281007	0.9	A/G	0.3777	0.7361	6.97E-02	2.99E-01	8.17E-01
rs13282455	81282397	0.89	G/A	0.3767	0.7458	9.21E-02	3.09E-01	8.71E-01
rs7837219	81283175	0.99	A/G	0.3499	0.4277	4.02E-01	8.25E-01	7.58E-01
rs7837153	81283376	0.99	C/A	0.3499	0.425	4.05E-01	8.47E-01	7.32E-01
rs10957978	81285139	0.89	T/G	0.3559	0.5357	3.78E-01	8.22E-01	4.76E-01
rs11784337	81285487	0.82	T/C	0.3757	0.693	6.11E-02	2.03E-01	8.58E-01
rs6473226	81285759	0.82	C/T	0.3748	0.6144	7.28E-02	1.66E-01	7.53E-01
rs6473227	81285892	0.82	A/C	0.3748	0.6149	7.76E-02	1.56E-01	7.65E-01
rs13263709	81287175	0.88	C/T	0.3519	0.4723	5.99E-01	8.26E-01	6.05E-01
rs952559	81288634	0.89	G/A	0.3549	0.4853	3.66E-01	6.20E-01	7.52E-01

rs952558	81288734	0.82	T/A	0.3748	0.6155	8.05E-02	1.56E-01	7.72E-01
rs952557	81288925	0.88	T/G	0.3519	0.4745	5.41E-01	7.91E-01	5.72E-01

rs992969 9p24

	hg19 position	r^2	RA/ EA	TGP EAF	CAAPA EAF	P_{assoc}	P_{anc}	P_{resid}
rs3939286	6210099	0.99	C/T	0.2525	0.4518	3.60E-04	9.66E-01	2.36E-01
rs928412	6213148	0.97	G/A	0.2575	0.3001	1.60E-03	8.05E-01	7.85E-01
rs928413	6213387	0.96	A/G	0.2594	0.4824	5.06E-03	9.92E-01	2.63E-01
rs7848215	6213468	0.93	C/T	0.2604	0.5696	3.88E-01	4.71E-01	8.39E-02

rs2589561 10p14

	hg19 position	r^2	RA/ EA	TGP EAF	CAAPA EAF	P_{assoc}	P_{anc}	P_{resid}
rs4116669	9040700	0.99	T/C	0.1879	0.1212	3.78E-02	9.71E-01	1.03E-01
rs4379740	9043304	0.99	C/T	0.1879	0.2549	2.15E-01	8.95E-01	1.45E-01
rs11256017	9043919	0.99	C/T	0.1879	0.1331	7.07E-02	7.43E-01	1.13E-01
rs2440781	9051328	0.99	G/A	0.1889	0.2531	2.73E-01	8.18E-01	1.42E-01
rs1775550	9052742	1	A/G	0.1899	0.1302	1.47E-01	8.18E-01	1.26E-01
rs2797288	9053173	1	A/G	0.1899	0.1336	1.48E-01	8.93E-01	1.19E-01

rs7927894 11q13
No SNPs met LD inclusion criteria

rs167769 12q13.3

	hg19 position	r^2	RA/ EA	TGP EAF	CAAPA EAF	P_{assoc}	P_{anc}	P_{resid}
rs3122929	57509102	0.93	C/T	0.34	0.2398	**9.13E-07**	4.70E-02	3.75E-01
rs3001425	57509569	0.92	C/T	0.3429	0.4173	**1.05E-05**	3.76E-03	2.97E-01

rs11071558 15q22.2

	hg19 position	r^2	RA/ EA	TGP EAF	CAAPA EAF	P_{assoc}	P_{anc}	P_{resid}
rs10519067	61068347	0.93	G/A	0.1352	0.3288	1.84E-07	1.48E-01	5.37E-01
rs10519068	61068704	0.92	G/A	0.1362	0.3787	7.14E-07	1.17E-01	8.52E-01
rs11071557	61068954	0.99	T/C	0.1412	0.3137	3.27E-07	2.86E-01	8.96E-01
rs34753162	61069177	0.99	T/C	0.1412	0.407	9.88E-04	8.87E-01	7.01E-01
rs34986765	61069201	1	T/C	0.1402	0.2055	6.28E-05	3.84E-01	7.73E-01
rs11071559	61069988	0.93	C/T	0.1342	0.3998	1.25E-03	9.78E-01	7.22E-01

rs2033784 15q22.33

	hg19 position	r^2	RA/ EA	TGP EAF	CAAPA EAF	P_{assoc}	P_{anc}	P_{resid}
rs1866316	67441997	0.99	T/C	0.2793	0.457	3.66E-01	4.66E-01	4.55E-01
rs8024330	67443926	0.98	C/T	0.2783	0.4717	2.70E-01	4.15E-01	2.65E-01
rs28434383	67448181	1	C/T	0.2813	0.4666	2.84E-01	4.09E-01	2.24E-01
rs8032739	67448899	1	A/G	0.2813	0.4722	2.69E-01	4.61E-01	2.80E-01
rs7173698	67450893	0.99	A/G	0.2813	0.4745	2.43E-01	4.63E-01	3.03E-01
rs7174445	67451215	0.99	C/G	0.2813	0.4711	2.71E-01	4.42E-01	2.89E-01
rs9920190	67452988	0.98	A/G	0.2793	0.4728	2.32E-01	4.74E-01	2.86E-01
rs7164786	67453042	0.98	G/A	0.2793	0.4727	2.31E-01	4.75E-01	2.85E-01

rs17806299 16p13.13

	hg19 position	r^2	RA/ EA	TGP EAF	CAAPA EAF	P_{assoc}	P_{anc}	P_{resid}
rs17806056	11192499	0.8	T/A	0.2306	0.0678	2.77E-01	1.25E-01	6.29E-01
rs35383879	11194823	0.96	G/A	0.1978	0.1648	7.18E-01	4.16E-01	3.16E-01
rs12923829	11197263	0.97	C/G	0.1958	0.0986	2.58E-01	1.06E-01	9.07E-01

	hg19 position	r^2	RA/ EA	TGP EAF	CAAPA EAF	P_{assoc}	P_{anc}	P_{resid}
rs35300161	11198932	0.99	C/T	0.1938	0.0906	1.53E-01	6.04E-02	9.06E-01
rs36110069	11207817	0.82	T/G	0.2286	0.0521	2.57E-02	1.49E-02	1.25E-01

rs17637472 17q21.33

	hg19 position	r^2	RA/ EA	TGP EAF	CAAPA EAF	P_{assoc}	P_{anc}	P_{resid}
rs28406364	47454507	0.83	C/T	0.3757	0.248	4.60E-02	9.32E-02	7.09E-01
rs28412876	47454515	0.83	G/T	0.3757	0.248	4.60E-02	9.33E-02	7.05E-01

Supplementary Table 19: Associations with tIgE. Lead SNPs associated with asthma in Table 2 was tested for association with total serum IgE (tIgE), separately in asthmatics and non-asthmatics (see Online Methods). All associations with P < 0.05 (in bold font) had a direction of effect consistent with the asthma association effect direction (higher tIgE = increased risk of asthma, lower tIgE = decreased risk of asthma).

			Non-asthmatics		Asthmatics		All	
**Locus**	**rsID**	**RA/EA**	**Beta [95%CI]**	**P**	**Beta [95%CI]**	**P**	**Beta [95%CI]**	**P**
**CAAPA**								
8p23	rs13277810	C/T	0.03 [-0.02-0.08]	0.261	0.02 [-0.03-0.07]	0.430	0.02 [-0.01-0.06]	0.180
8q24	rs114647118	C/T	-0.01 [-0.17-0.14]	0.857	-0.13 [-0.32-0.06]	0.164	-0.06 [-0.19-0.06]	0.303
12q13	rs3122929	C/T	-0.02 [-0.06-0.03]	0.482	0 [-0.04-0.03]	0.803	-0.01 [-0.04-0.02]	0.513
15q22	rs10519067	G/A	-0.01 [-0.05-0.03]	0.495	**-0.04 [-0.08--0.01]**	**0.022**	**-0.03 [-0.06-0.00]**	**0.033**
17q12-21	rs907092	G/A	0.01 [-0.03-0.06]	0.592	**-0.06 [-0.1--0.02]**	**0.003**	-0.03 [-0.06-0.00]	0.07
**TAGC**								
2q12	rs1420101	C/T	0.00 [-0.03-0.04]	0.836	**0.04 [0.00-0.07]**	**0.039**	0.02 [0.00-0.05]	0.098
5q22.1	rs10455025	A/C	0.04 [-0.03-0.11]	0.255	-0.01 [-0.07-0.04]	0.627	0.01 [-0.04-0.05]	0.731
5q31	rs20541	A/G	0.02 [-0.03-0.06]	0.452	0.00 [-0.04-0.03]	0.818	0 [-0.02-0.03]	0.746
6p22	rs1233578	A/G	0.00 [-0.04-0.03]	0.848	0.03 [-0.01-0.06]	0.145	0.01 [-0.01-0.04]	0.349
6p21	rs9272346	G/A	**0.04 [0.00-0.07]**	**0.039**	0.01 [-0.03-0.04]	0.714	0.02 [0.00-0.05]	0.092
9p24	rs992969	A/G	0.01 [-0.03-0.05]	0.701	0.01 [-0.02-0.05]	0.519	0.01 [-0.02-0.03]	0.463
11q13	rs7927894	C/T	0.01 [-0.03-0.05]	0.600	-0.01 [-0.05-0.02]	0.520	0.00 [-0.03-0.02]	0.894
12q13	rs167769	C/T	-0.01 [-0.05-0.04]	0.794	0.01 [-0.03-0.05]	0.485	0.01 [-0.02-0.03]	0.731
15q22	rs2033784	A/G	-0.01 [-0.05-0.02]	0.498	-0.02 [-0.06-0.01]	0.146	-0.02 [-0.04-0.01]	0.127
17q12-21	rs2952156	A/G	0.01 [-0.02-0.05]	0.486	**-0.04 [-0.07-0.00]**	**0.026**	-0.02 [-0.04-0.01]	0.245

Supplementary Table 20: Summary of the admixture mapping peaks on chromosome 6 in the admixture mapping discovery data set. A linear mixed model (EMMAX software) was used to test for association between the number of copies of African ancestry ("dosage" value of 0, 1, 2) and case-control status (2,608 cases and 3,994 controls). Since ADPC data was only available for SAPPHIRE cases, the study was not included in the model. The most significant segment is highlighted in bold font. Z is the standardized deviation between the mean African ancestry of a local ancestry segment in cases/controls/SAPPHIRE, and the grand African ancestry mean in cases/controls/SAPPHIRE (the means and Z were calculated separately for each of these 3 groups).

Chr:start-end hg19 position	Linear model Beta	P-value	Proportion African ancestry Case mean	Control mean	SAPPHIRE mean	Case Z	Control Z	SAPPHIRE Z
6:132,167,290-132,217,687	0.0261	0.0078	0.8240	0.8078	0.8130	1.2247	-0.0302	1.0881
6:132,253,941-132,475,231	0.0246	0.0124	0.8230	0.8075	0.8103	1.0782	-0.0909	0.8581
6:132,509,041-132,647,736	0.0267	0.0069	0.8259	0.8095	0.8116	1.5178	0.2328	0.9731
6:132,664,724-132,753,715	0.0303	0.0021	0.8265	0.8080	0.8171	1.6058	-0.0100	1.4331
6:132,765,427-132,889,619	0.0345	0.0004	0.8244	0.8037	0.8123	1.2833	-0.6978	1.0306
6:132,891,570-132,908,750	0.0349	0.0003	0.8230	0.8017	0.8089	1.0782	-1.0215	0.7432
6:132,928,872-133,036,860	0.0341	0.0005	0.8236	0.8030	0.8151	1.1661	-0.8192	1.2606
6:133,052,616-133,334,308	0.0354	0.0003	0.8242	0.8046	0.8219	1.2540	-0.5562	1.8355
6:133,370,646-133,727,983	0.0362	0.0002	0.8250	0.8052	0.8253	1.3713	-0.4551	2.1230
6:133,731,403-133,892,116	0.0336	0.0006	0.8221	0.8028	0.8253	0.9316	-0.8394	2.1230
6:133,897,107-134,149,081	0.0359	0.0002	0.8211	0.8008	0.8212	0.7850	-1.1631	1.7780
**6:134,149,974-134,300,365**	**0.0370**	**0.0001**	**0.8230**	**0.8013**	**0.8274**	**1.0782**	**-1.0822**	**2.2955**
6:134,319,531-134,377,316	0.0334	0.0003	0.8131	0.7917	0.8144	-0.4461	-2.6399	1.2031
6:134,380,130-134,413,537	0.0339	0.0002	0.8119	0.7904	0.8075	-0.6220	-2.8423	0.6282
6:134,416,282-134,470,574	0.0329	0.0003	0.8133	0.7924	0.8075	-0.4168	-2.5186	0.6282
6:134,485,169-134,517,263	0.0301	0.0011	0.8137	0.7936	0.8055	-0.3581	-2.3365	0.4557
6:134,538,881-134,629,970	0.0290	0.0018	0.8144	0.7952	0.8103	-0.2409	-2.0735	0.8581
6:134,685,508-134,737,470	0.0310	0.0011	0.8219	0.8017	0.8192	0.9023	-1.0215	1.6056
6:134,740,669-134,845,333	0.0301	0.0016	0.8209	0.8016	0.8171	0.7557	-1.0418	1.4331
6:134,873,987-135,015,339	0.0282	0.0035	0.8213	0.8040	0.8158	0.8143	-0.6574	1.3181
6:135,051,669-135,237,744	0.0265	0.0056	0.8175	0.8023	0.8144	0.2281	-0.9204	1.2031
6:135,243,026-135,559,215	0.0237	0.0135	0.8163	0.8046	0.8185	0.0522	-0.5562	1.5481

Supplementary Table 21: Summary of the association between selected local ancestry segments and asthma in the admixture mapping replication data set. The segments including the start and end position of the admixture mapping discovery peak (chr6:134,149,974-134,300,365) were selected for replication. Logistic regression was used to test for association between the number of copies of African ancestry ("dosage" value of 0, 1, 2) and case-control status, separately for each replication data set (BASS, BAGS, JAAS, BioMe). The results for the segment including/closest to the midpoint of the admixture mapping discovery peak (134,225,170) was combined using inverse-variance meta-analysis.

Study	Chr:start-end hg19 position	OR [95% CI]	P-value
BASS (135 cases + 216 controls)	6:133,993,445-134,237,057	0.91 [0.59-1.40]	0.6692
	6:134,248,060-134,324,616	0.87 [0.57-1.32]	0.5030
BAGS (180 cases + 213 controls)	6:134,096,486-134,243,420	0.83 [0.51-1.33]	0.4298
	6:134,248,060-134,325,029	0.85 [0.53-1.36]	0.4936
JAAS (167 cases + 507 controls)	6:133,993,445-134,237,057	0.81 [0.55-1.20]	0.2982
	6:134,245,229-134,321,449	0.78 [0.53-1.16]	0.2196
BioME (363 cases + 1,407 controls)	6:133,869,809-133,869,809	1.07 [0.83-1.38]	0.5991
	6:134,431,870-134,431,870	1.06 [0.82-1.37]	0.6365
Combined (845 cases + 2,343 controls)		0.95 [0.79-1.13]	0.5423

Supplementary Table 22: 99% credible set of SNPs for the novel CAAPA asthma associations on chromosome 8p23. SNPs +-10KB from the lead SNP at hg19 position 2,550,802 were included in this analysis. The posterior probability of association for each SNP is the ratio of its Bayes Factor over the sum of the Bayes Factors of all the SNPs included in the analysis. SNPs were then ranked according to their Bayes Factors, and SNPs were selected for inclusion in the 99% credible set until their cumulative posterior probability attained or exceeded 0.99.

rsID	hg19 position	P-value association	Bayes Factor	Posterior probability	Cumulative posterior probability
rs13277810	2550802	3.10E-08	14.4408	0.3893	0.3893
rs13279298	2551365	4.30E-08	14.1303	0.2854	0.6747
rs13266991	2550275	1.20E-07	13.0761	0.0995	0.7742
rs13277427	2550131	1.30E-07	13.0584	0.0977	0.8719
rs34015808	2548821	1.70E-07	12.7525	0.072	0.9438
rs13269769	2550492	5.10E-07	11.658	0.0241	0.9679
rs79954731	2548759	9.60E-07	11.0197	0.0127	0.9806
rs71501048	2551896	2.40E-06	10.1271	0.0052	0.9858
rs13439672	2548916	3.00E-06	9.8852	0.0041	0.9899
rs13439436	2548898	3.40e-06	9.7708	0.0036	0.9936

Supplementary Table 23: The proportion of SNPs deleted and retained in the imputed datasets by dataset and MAF category. SNPs with MAF ≤ 0.005 were deleted if Rsq ≤ 0.5 and SNPs with MAF > 0.005 were deleted if Rsq ≤ 0.3.

	Nr MAF ≤ 0.005 SNPs deleted (%)	Nr MAF ≤ 0.005 SNPs retained (%)	Nr MAF > 0.005 SNPs deleted (%)	Nr MAF > 0.005 SNPs retained (%)	Nr SNPs remaining
**BASS**	8,604,300 (76%)	2,763,691 (24%)	1,099,421 (6%)	16,187,099 (94%)	18,950,790
**GRAAD(1)**	8,614,896 (69%)	3,851,948 (31%)	931,948 (5%)	16,424,959 (95%)	20,276,907
**GRAAD(2)**	11,666,786 (96%)	446,637 (4%)	543,562 (3%)	17,166,766 (97%)	17,613,403
**CAG**	9,440,080 (76%)	2,988,080 (24%)	669,391 (4%)	16,726,200 (96%)	19,714,280
**SAPPHIRE***	8,593,122 (69%)	3,863,294 (31%)	843,889 (5%)	16,523,446 (95%)	20,386,740
**JHS(1)**	9,898,914 (79%)	2,666,990 (21%)	1,116,492 (6%)	16,124,611 (94%)	18,791,601
**JHS(2)**	8,793,769 (71%)	3,657,343 (29%)	952,901 (5%)	16,402,994 (95%)	20,060,337
**SAGE II**	8,073,662 (65%)	4,396,838 (35%)	887,008 (5%)	16,466,243 (95%)	20,863,081
**HUFS**	8,290,986 (67%)	4,146,355 (33%)	956,849 (6%)	16,429,561 (94%)	20,575,916
**SARP**	10,029,987 (80%)	2,578,587 (20%)	1,194,584 (7%)	16,003,849 (93%)	18,582,436
**BAGS**	9,567,906 (76%)	3,069,717 (24%)	947,981 (6%)	16,221,403 (94%)	19,291,120
**BIAS**	8,815,134 (81%)	2,134,915 (19%)	1,134,324 (7%)	15,097,128 (93%)	17,232,043
**ProAR**	7,951,397 (66%)	4,031,692 (34%)	1,079,904 (6%)	15,676,573 (94%)	19,708,265
**PGCA**	8,283,260 (66%)	4,340,258 (34%)	1,175,488 (7%)	14,690,685 (93%)	19,030,943
**HONDAS**	8,926,343 (83%)	1,843,078 (17%)	1,067,135 (7%)	14,236,230 (93%)	16,079,30
**JAAS**	7,953,650 (68%)	3,694,588 (32%)	1,014,375 (6%)	16,189,824 (94%)	19,884,412
**GALA II**	10,453,163 (70%)	4,406,580 (30%)	1,218,368 (8%)	13,745,640 (92%)	18,152,220

* Using ADPC data of 730 cases

Supplementary Table 24: Software used for data analysis. With the exception of software packages used for calling genotype data, this table summarizes the software packages used for processing and analyzing data.

Software package	URL	Application	Parameters
ADMIXTURE 1.3.0	https://www.genetics.ucla.edu/software/admixture/	Estimating genome-wide proportions of ancestry	K=3
ANNOVAR	http://annovar.openbioinformatics.org	Gene, filter and region- based annotation. All reported chromosome position (e.g. chr8p12) are based on ANNOVAR annotations.	
CrossMap v0.2.1	http://crossmap.sourceforge.net	Lifting over genomic coordinates of the ARIC and JHS data set	Chain file used: *hg18ToHg19.over.chain*
EMMAX 07Mar2010	http://genetics.cs.ucla.edu/emmax/	Tests for associations in admixture mapping discovery	Balding-Nichols kinship matrix estimation
GAS Power Calculator	http://csg.sph.umich.edu/abecasis/cats/gas_power_calculator/		
Genesis	http://www.bioinf.wits.ac.za/software/genesis/	PC and ADMIXTURE plots	
Haploview 4.2	https://www.broadinstitute.org/haploview/downloads	LD plots for chr17q12-21 candidate SNPs; LD block and r^2 estimated between TAGC lead SNPs and surrounding SNPs in TGP Europeans and Africans.	Color scheme=R-squared, Blocks defined using Gabriel confidence intervals
HUGIn	https://yunliweb.its.unc.edu/hugin/index.php	Visualization of chromatin organizations surrounding the novel genome-wide CAAPA SNP on chr8p23	
ImpG-Summary v1.0.1	http://bogdan.bioinformatics.ucla.edu/software/impg/	Imputation of genotyped asthma associations in BioMe for the purposes of replication	The CAAPA WSG reference panel was used
KING 1.4	http://people.virginia.edu/~wc9c/KING/index.html	Estimating kinship matrixes for inclusion in association tests	Default parameters were used
LocusZoom 1.4 (05/01/2017)	https://statgen.sph.umich.edu/locuszoom/download/locuszoom_1.4_srconly.tgz	Locus zoom plots	CAAPA African American controls in WGS reference panel were used to estimate r^2 between SNPs
Minimac3	https://imputationserver.sph.umich.edu	Imputation of CAAPA data sets	*Reference Panel:* CAAPA – African American Panel<br>*Phasing:* ShapeIT<br>*Population:* AA (CAAPA)<br>Imputations for GWAS+ADPC data were run March-April 2016, and imputations with masked genotypes (to assess imputation accuracy) were run July 2016. Imputation for MEGA data sets were run September 2017.
METAL 2011-03-25	http://csg.sph.umich.edu/abecasis/Metal/download/	Inverse-variance meta-analysis for estimating effect sizes, input for locus zoom plots of admixture mapping peak region, chr17q12-21 locus zoom plots of associations in studies with high and low African ancestry associations, meta-analysis of African American studies	
MR-MEGA v.0.1.2	https://www.geenivaramu.ee/en/tools/mr-mega	Meta-analysis of CAAPA data sets	
PLINK v1.9	https://www.cog-genomics.org/plink2/	Sample and SNP QC, LD filtering, MAF filtering, strand flipping, data set merging, logistic regression association tests for imputed SAPPHIRE data and Bio*Me* genotyped da-a.	
R 3.2.5 – 3.4.2	https://cran.r-project.org	Data visualization and data set merging; statistical tests	
forestplot R	https://cran.r-project.org	Forest plots	

Software package	URL	Application	Parameters
package 1.7.2			
haplo.stats R package 1.7.7	https://cran.r-project.org	Haplotupe association of rs12936231 and rs4065275	
GENESIS R Bioconductor package 2.4.0	https://www.bioconductor.org/packages/devel/bioc/html/GENESIS.html	Tests for association in the CAAPA data sets, Principal component analysis	
RFMix v1.5.4	https://sites.google.com/site/rfmixlocalancestryinference/	Local ancestry inference	Default parameters were used
SeattleSeqAnnotation138	http://snp.gs.washington.edu/SeattleSeqAnnotation138	Used to obtain rsIDs for SNPs from chromosome and hg19 base pair positions	
ShapeIt v2.r790 v2.r837	https://mathgen.stats.ox.ac.uk/genetics_software/shapeit/shapeit.html	v2.r790 was used for phasing of the CAAPA WGS imputation reference panel, v2.r837 was used for genotype phasing for local ancestry inference, and v2.r837 was used for phasing of the CAAPA WGS reference panel for ImpG imputation of BioMe summary statistics	b37 genetic map files were used
VCFtools 0.1.13	https://vcftools.github.io/index.html	SNP filtering based on Rsq values.	

Supplementary Notes

Supplementary Note 1: Participants and disease definitions of studies used in CAAPA discovery meta-analysis

CAAPA investigators previously recruited African ancestry participants into asthma genetics studies across 14 geographical sites (Table 1). Participants in these studies were unrelated cases and controls, except for subjects from the Barbados Asthma Genetics Study (BAGS), where a family-based study design was used, the Brazilian Immunogenetics of Asthma and Schistosomiasis (BIAS) study, where a whole-population ascertainment was used (including families), and the Howard University Family Study (HUFS) where participants were a mixture of unrelated and related subjects. Definition of asthma was based on a doctor's diagnosis and/or standardized questionnaires; controls were defined as a reported negative history of asthma. The distributions of age, sex and age of asthma onset are summarized in Supplementary Table S1.

The **Baltimore Asthma Severity Study (BASS)** is a community-based convenience sample of African American Baltimore City residents, including physician-diagnosed asthmatic cases and control subjects [1]. Participants responded to a standardized, interviewer-administered questionnaire that includes a modified version of the 1987 American Thoracic Society Division of Lung Disease Epidemiology Questionnaire to collect information on asthma and allergy symptoms. The questionnaire was used to determine whether a subject has a history of both self-reported and physician-diagnosed asthma (asthma case), or had no history of asthma (healthy control).

The **Genomic Research on Asthma in the African Diaspora (GRAAD)** consortium represent 8 studies of asthma in African American children and adults, and one additional study on healthy African Americans. GRAAD study subjects were recruited from the Baltimore-Washington DC metropolitan area, and all subjects used in this genome-wide association study (GWAS) self-reported as African American. Case-control status was defined as previously described [1]. Briefly, standardized questionnaires administered by a clinical coordinator were used to determine whether a subject has a history of both self-reported and physician-diagnosed asthma (asthma case), or had no history of asthma (healthy control). Adult controls were favored to minimize the inclusion of subjects that may still develop asthma.

The **Chicago Asthma Genetics (CAG)** study comprises European American and African American families ascertained through affected sib pairs, case-parent trios (through affected offspring), adults and children with

severe persistent asthma, and non-asthmatic control subjects (> 18 years) [2]. As described previously in the supplementary note of [3], asthma cases and families were recruited in the adult and/or pediatric asthma clinics at University of Chicago Hospital; controls were recruited from the medical center at large. Asthma was defined based on the following criteria: physician's diagnosis, the presence of at least 2 self-reported symptoms (cough, wheeze, shortness of breath), current use of asthma medications, and either bronchial hyperresponsiveness or reversibility to inhaled bronchodilator. Subjects with a birthweight below 4.4 lb or with > 3 pack-years of cigarette smoking were excluded. Controls had no self-reported personal or family history of asthma among first- degree relatives.

The **Study of Asthma Phenotypes and Pharmacogenomics Interactions by Race-ethnicity (SAPPHIRE)** is a longitudinal, population-based to identify the genetic predictors of asthma medication treatment response and the genetic underpinnings of asthma-related phenotypes [4]. Eligible patients receive(d) care from a large healthcare system serving southeast Michigan and the greater Detroit metropolitan area. At the time of study enrollment, case patients were ≥12 years of age, had a documented physician diagnosis of asthma in the medical record, and had no prior diagnosis of congestive heart failure or chronic obstructive pulmonary disease [5, 6]. Control patients had the same enrollment criteria as case patients with the exception of having no prior diagnosis of asthma.

The **Jackson Heart Study (JHS)** [7] is a prospective population-based study to seek the causes of the high prevalence of common complex diseases among African Americans in the Jackson, Mississippi metropolitan area. During the baseline examination period (2000-2004) 5,301 self-identified African Americans were recruited from four sources, including (1) randomly sampled households from a commercial listing; (2) ARIC participants; (3) a structured volunteer sample that was designed to mirror the eligible population; and (4) a nested family cohort. Unrelated participants were between 35 and 84 years old, and members of the family cohort were ≥ 21 years old when consent for genetic testing was obtained and blood was drawn for DNA extraction. Asthma status was defined using a combination of self-report of current asthma or ever being diagnosed with asthma, and asthma medication status. Asthma controls were further required to have FEV1 predicted > 90%.

The **Study of African Americans, Asthma, Genes & Environments (SAGE II)** is an ongoing population-based, case-control study recruiting African American participants from clinics in the San Francisco Bay Area [8]. Subjects were eligible if they were 8-21 years of age, self-identified all four grandparents as African Americans, and had <10 pack-years of smoking history. Asthma was defined by physician diagnosis and report of symptoms in the 2 years preceding enrollment. Controls had no reported history of asthma, eczema, hives, hay fever, allergic rhinitis, no reported use of medication for allergies and no reported symptoms of wheezing or shortness of breath during their lifetime

The **Howard University Family Study (HUFS)** is a population-based family study of African Americans in the Washington metropolitan area. African American families, as well as unrelated individuals, were randomly ascertained to study the genetic and environmental basis of common complex diseases [9]. Asthma was defined based on self-reported physician diagnosis and medication history [10]. Controls were defined based on absence of physician-diagnosed asthma and no history of asthma medication.

The **NHLBI-sponsored Severe Asthma Research Program (SARP1-2)** is a multi-ethnic cohort of comprehensively characterized subjects with mild to severe asthma enriched for severe disease. SARP1-2 is an integrated study of the clinical and biological features of asthma with the goal of understanding the cellular and molecular mechanisms underlying asthma and its endotypes. Study participants that self-reported as African American were included in the CAAPA GWAS. A diagnosis of asthma in SARP was based on evidence of methacholine bronchial hyperresponsiveness or bronchodilator reversibility in combination with asthma symptoms [11]. On the other hand, controls in SARP were defined by not having chronic lung disease or collateral allergic disease and the absence of a significant FEV1 bronchodilator response.

Probands from the **Barbados Asthma Genetics Study (BAGS)** were recruited through referrals as described previously [1, 12, 13]. Nuclear and extended family members were also enrolled in the study. Asthma was defined based on a history of both self-reported and physician-diagnosed asthma, as well as a history of wheezing without an upper respiratory tract inflection [1]. Controls were selected based on no history of asthma.

The **Brazilian Immunogenetics of Asthma and Schistosomiasis (BIAS)** study is a whole-population ascertainment designed study of asthma and schistosomiasis in the rural district of Conde, Bahia, located 200 km north of Salvador, Brazil [14-16]. Subjects were recruited July – September, 2004, from five communities (Buri, Camarao, Genipapo, Sempre Viva, and Cobo), and follow-up visits continue. To date, 822 subjects have been enrolled from an estimated population of 1,700. The dataset is comprised of 2 large pedigrees with 535 and 310 members collapsed into 318 nuclear families (aged 5-85 years). Asthmatics/non-asthmatics were identified on the basis of a modified ISAAC questionnaire [17-19], and as infection with schistosomiasis may mask some of the symptoms of asthma [20], the presence of wheezing was the primary criteria for defining asthma.

A **Program for Control of Asthma (ProAR)** was developed in Salvador, Bahia, Brazil to manage the most severe forms of asthma, previously untreated, from 2003. In 2013, we selected a sample of 544 subjects (≥18 years old) followed up for more than 6 months, performed spirometry, skin prick test for relevant aeroallergens, Total IgE, blood cell counts, a full panel of other blood tests to investigate comorbidities, and a chest radiography, having a diagnosis of asthma validated by two experts who excluded those with comorbidities that could interfere with the assessment of asthma. All subjects lived in the City of Salvador or its metropolitan area, attended regularly the clinic and filled out their prescriptions. This sample have been followed up closely and reviewed meticulously on two additional occasions. The cohort of subjects with previously untreated severe asthma has been compared to a control cohort of 452 adult unrelated subjects with a medical diagnosis of mild to moderate asthma and recruited from the same communities, who have undergone the same study procedures. Another control group (n=450), unrelated and with no asthma, has been evaluated using the same protocol. For the CAAPA meta-analysis, subjects with a medical diagnosis of asthma were classified as cases, and subjects with no asthma were classified as controls.

The **Proyecto Genes Candidatos en Asma (PGCA) study** is a population-based study conducted by the Institute for Immunological Research of The University of Cartagena (Colombia) to identify environmental and genetic risk factors for asthma and allergies [16, 19, 21, 22]. A total of 836 unrelated asthma cases and 574 non-asthmatic controls were recruited from the Social Security Clinic and outpatient health centers in Cartagena from 2002 to 2005. As described previously [19], asthma was defined using GINA criteria [23] by using a standardized questionnaire previously tested [24, 25] in patients with a history of physician-diagnosed asthma. The diagnosis was confirmed by a physician belonging to the research staff, sustained on a clear clinical history with clinical symptoms and the presence of reversible airways disease on pulmonary function testing. The control group comprised unrelated individuals without a personal or familiar history of asthma or allergies [26].

The **Honduras Genetics of Asthma in Non-European Populations (HONDAS)** study is a population-based study of asthma and population dynamics, structure, and phylogenetic relations of the Garífuna (Black Carib) people from the northern coast of Honduras, a population of African and Red Carib Native Amerindian ancestry [27, 28]; and of Honduran autochthonous Amerindian populations of Mesoamerican or South American ancestry. To date, 858 subjects have been recruited from 12 villages (Bajamar, Travesía, Corozal, Sambo Creek, Alfonzo Lacayo, Belén Gualcho, San Juan, Tornabé, Triunfo de la Cruz, Cristales, Río Negro, and Santa Fe ; aged 5-85). Participants responded to a standardized, interviewer-administered questionnaire that includes a modified version of the 1987 American Thoracic Society Division of Lung Disease Epidemiology Questionnaire to collect information on asthma and allergy symptoms. The questionnaire was used to determine whether a subject has a history of both self-reported and physician-diagnosed asthma (asthma case), or had no history of asthma (healthy control).

The **Jamaican Adolescent Asthma Study (JAAS)** is a cross-sectional study on the prevalence of asthma and allergies in 897 Jamaican adolescents [29]. Participants' mothers were initially recruited into the Jamaica Perinatal Mortality Survey of 1986, which included all children born in Jamaica in September—October 1986. Children from Kingston, St. Andrew, and St. Catherine were contacted at ages 11 and 16 as part of a child development study, and again at age 18. As described previously [29], a modified International Study of Asthma and Allergies in Childhood (ISAAC) questionnaire which had been validated in the Jamaican population was administered. Participants who responded positively to the question regarding a doctor's diagnosis of asthma were diagnosed as asthmatic. Adults had been followed in the clinic since birth, and those whose medical records indicated a history of at least three episodes of wheezing, at least one of which occurred after the age of six years, were also classified as having asthma.

The **Genes-environments & Admixture in Latino Americans (GALA II**) is an ongoing population-based, case-control study recruiting Latino children from five centers (Chicago, Illinois; Bronx, New York; Houston, Texas; San Francisco Bay Area, California; and Puerto Rico) [30]. Subjects were eligible if they were 8-21 years of age and all four grandparents self-identified as Latino. The definition of asthma cases and controls followed the same protocols as SAGE II. Only subjects recruited in Puerto Rico were included in the CAAPA GWAS.

Supplementary Note 2: Participants and disease definitions of replication studies

CARDIA (Coronary Artery Risk Development in Young Adults)

The CARDIA [31] study is a prospective, multi-center investigation of the natural history and etiology of cardiovascular disease in African Americans and whites 18-30 years of age at the time of initial examination (http://www.cardia.dopm.uab.edu/index.htm). The CARDIA sample was recruited at random during 1985-86 primarily from geographically based populations in Birmingham AL, Chicago IL, and Minneapolis MN and, in Oakland, CA, from the membership of the Kaiser-Permanente Health Plan. The initial examination included 5,115 participants selectively recruited to represent proportionate racial, gender, age, and education groups from each of the four communities. Each participant's age, race, and sex were self-reported during the recruitment phase and verified during the baseline clinic visit. Details of the study design and procedures for data collection have been published [32]. From the time of initiation of the study in 1985-1986 (baseline examination), six follow-up examinations have been conducted at years 2, 5, 7, 10, 15, 20, and 25. Asthma case and control status was defined based on self-report of ever having asthma, ascertained at the first study visit.

MESA (Multi-Ethnic Study of Atherosclerosis)

MESA [33] is a National Heart, Lung and Blood Institute-sponsored, population-based investigation of subclinical cardiovascular disease and its progression. A total of 6,814 individuals, aged 45 to 84 years, were recruited from six US communities (Baltimore City and County, MD; Chicago, IL; Forsyth County, NC; Los Angeles County, CA; New York, NY; and St. Paul, MN) between July 2000 and August 2002. Participants were excluded if they had physician-diagnosed cardiovascular disease prior to enrollment, including angina, myocardial infarction, heart failure, stroke or TIA, resuscitated cardiac arrest or a cardiovascular intervention (e.g., CABG, angioplasty, valve replacement, or pacemaker/defibrillator placement). Pre-specified recruitment plans identified four racial/ethnic groups (White European-American, African-American, Hispanic-American, and Chinese-American) for enrollment, with targeted oversampling of minority groups to enhance statistical power. Asthma case and control status was defined based on self-report of ever having asthma, ascertained at the first study visit.

ARIC (Atherosclerosis Risk in Communities Study)

The ARIC [34] study is a multi-center prospective investigation of atherosclerotic disease in a predominantly bi-racial population. Men and women aged 45-64 years at baseline were recruited from 4 communities: Forsyth

County, North Carolina; Jackson, Mississippi; suburban areas of Minneapolis, Minnesota; and Washington County, Maryland. A total of 15,792 individuals participated in the baseline examination in 1987-1989, with follow-up examinations in approximate 3-year intervals, during 1990-1992, 1993-1995, and 1996-1998. While the Jackson sample includes African Americans only, the other field centers samples are representative of the populations in these communities (i.e., mostly White in Minneapolis and Washington County, and about 15% African American in Forsyth County). Approximately 4,000 participants were recruited from each community. The resulting study population examined at the ARIC baseline examination (15,792 subjects) was 55% female and 27% African American. Asthma case and control status was defined based on self-report of ever having been diagnosed with asthma by a doctor, ascertained at the first study visit.

Bio*Me*

Asthma case-control status in BioMe was defined using a modified version of the eMERGE asthma case-definition algorithm [35]. Cases were defined by either the presence of ICD9 '493.xx' or self-reporting based on the BioMe questionnaire. Individuals who self-reported being smokers and those who's EHR contained ICD9 codes for other respiratory diseases were excluded from 'case' status. Controls were defined as individuals who's EHR did not contain the ICD9 code '493.xx' and who self-reported that they did not have asthma. Individuals who self-reported being smokers and those who's EHR contained ICD9 codes for other respiratory diseases or blood cancers were excluded from 'control' status. African American and Hispanic subjects with GWAS array data were used to test for replication of the CAAPA GWAS meta-analysis results, and African American subjects with local ancestry calls were used for admixture mapping replication. Most of the Hispanic subjects are Caribbean, with 704/2288 identifying Puerto Rico. The other largest groups identify as "Mixed" (N=387), Ecuador (N=155), Dominican Republic (N=547), and Mexico (N=142). The remaining participants have ancestry from South and Central America (Peru, Argentina, Brazil, El Salvador, Guatemala, Panama, Colombia, and more).

Supplementary Note 3: CAAPA WGS reference panel participants and disease definitions

The whole genome sequence (WGS) reference panel that was used for genotype imputation is comprised of 880 unrelated subjects. Participant selection and disease definition is summarized in detail in Supplementary Appendix of Mathias et al. [36], but the note only describes the 642 subjects in the data freeze used for that study's analysis. The final data freeze of 880 subjects used for genotype imputation in our association study is summarized in Supplementary Table S3. Samples collected from the JHS, Gabon and Palenque were not available in the previous data freeze (642 subjects), but are available in the final data freeze (880 subjects). The JHS is described above (under CAAPA ADPC Participants and Disease Definitions). Asthma was not defined for the subjects from Gabon and Palenque.

Supplementary Note 4: GWAS backbone genotyping, SNP calling and quality control

Many of the CAAPA studies were genotyped on a variety of GWAS backbones (Table 1). Each of the study sites was responsible for and performed their own genotype calling and genomic coordinate transformation where applicable, and for most datasets, this has been described previously (GRAAD(1)+BAGS: [1], CAG: [37], SAPPHIRE: [4] ,JHS: [19], SAGE II: [38], HUFS: [39], GALA II [30]). Exceptions are described below.

GRAAD(2)

Samples from GRAAD participants that were recruited after the initial GWAS study [1] were genotyped on the Illumina Omni2.5 BeadChip at the Lowe Family Genomics core, Johns Hopkins. Genotypes were called following recommendations from Illumina Infinium Technical Notes implemented in Illumina's GenomeStudio software (v2011.1).

SNPs that were out of HWE (p-value < 10^{-6}) and SNPs with a missing call rate > 0.1 were dropped from the dataset. Samples with a missing call rate > 0.01 and samples with gender discrepancies (using F-statistics estimated by PLINK, samples with F(inbreeding males)<0.2 or F(inbreeding females)>0.5 defined as miss-classified) were dropped from the dataset. PLINK was used to estimate IBD between all pairs of subjects, using a set of linkage disequilibrium (LD)-pruned SNPs. Duplicates (PLINK PI_HAT > 0.9, remove both individuals) and related individuals (PLINK PI_HAT > 0.35, remove at least one individual) were then dropped from the dataset.

JHS

After receiving the JHS datasets from the study coordinators, genomic coordinates were translated using CrossMap (http://crossmap.sourceforge.net) and the hg18tohg19 chain file published with this software.

SARP

Genotypes were called using the GenomeStudio clustering algorithm. DNA samples with call rates <0.90 were excluded. SNPs with call frequencies <0.90 were excluded and clustering was repeated followed by exclusion of samples with a call rate <0.98. After updating SNP statistics, a second round of SNP exclusion was performed for cluster separation scores <0.20 followed by SNPs with score <0.30 and a GenTrain score <0.75. After clustering and genotype calling, QC was performed using PLINK. SNPs with a MAF >0.01 with HWE p<0.001 in unrelated subjects were removed. Sex was determined for all subjects using chromosome X data and subjects with a mismatch with self-reported sex data were excluded. IBD tests were also performed to exclude identical or related samples.

Supplementary Note 5: ADPC genotyping, SNP calling and quality control

10,411 CAAPA samples, 346 HapMap controls and 217 internal samples were genotyped at Illumina on the ADPC. 306 samples failed QC at Illumina. For the remaining 9,888 CAAPA samples, sample quality control was performed in the following order:
1. Remove IBD duplicates (n=193; performed on 113,324 LD-pruned SNPs filtered for SNPs that have Hardy-Weinberg equilibrium (HWE) p-values > 1e-6 and MAF > 0.05; samples with PLINK PI_HAT < 0.9 were classified as IBD duplicates)
2. Remove samples with a high proportion of missing genotypes (n=0, more than 1% missing genotypes)
3. Remove samples with gender discrepancies (n=28, using F-statistics estimated by PLINK, samples with F(inbreeding males)<0.2 or F(inbreeding females)>0.5 were removed)
4. Remove samples with excess heterozygosity (n=2, using F-statistics estimated by PLINK, samples with F(inbreeding)>0.25).
An additional 364 samples without existing GWAS data, and 71 samples from two studies too small for accurate imputation (the imputation server requires at least 50 samples) were also removed, leaving 9230 samples.

Preliminary SNP QC was performed using GenomeStudio. 130,637 SNPs that failed one or more of the following criteria were removed, as recommended by Illumina:
1. Call frequency < 0.97
2. Replicate errors > 2
3. Parent-parent-child (PPC) errors > 2
4. Cluster separation <= 0.3
5. Mean normalized intensity of first homozygote cluster (AA_R_Mean) <= 0.2 and/or mean normalized intensity of heterozygote cluster (AB_R_Mean) <= 0.2 and/or mean normalized intensity of second homozygote cluster (BB_R_Mean) <=0.2
6. Mean of normalized theta value for first homozygote cluster (AA_T_Mean) > 0.3
7. Standard deviation of normalized theta value for first homozygote cluster (AA_D_Mean) > 0.06

8. Mean of normalized theta value for second homozygote cluster (BB_T_Mean) < 0.7
9. Standard deviation of normalized theta value for second homozygote cluster (BB_D_Mean) > 0.06.

Supplementary Note 6: Merging and imputation of GWAS and ADPC datasets

After receiving GWAS SNP array data from the various study centers, a pipeline of custom scripts was used to apply consistent quality control (QC) metrics to the GWAS datasets and to merge autosomal content from the GWAS and ADPC datasets. The pipeline was applied separately to each of the GWAS datasets listed in Table 1 (for the JHS, genotyping on the Affymetrix 6.0 array was performed in two batches, and these batches were therefore treated as separate datasets). Each of the datasets were uploaded separately to the imputation server hosted by the University of Michigan (https://imputationserver.sph.umich.edu; for the imputation configuration, the CAAPA - African American reference panel was used, the configured population was AA (CAAPA), and ShapeIT was used for phasing).

The steps applied in the pipeline were as follows:
1. Non-autosomal SNPs and SNPs with duplicate positions were removed from the GWAS dataset. Samples that were not typed successfully on the ADPC array were also removed. SNPs that failed a test for Hardy-Weinberg equilibrium (HWE; p-value < 0.0001), monomorphic SNPs and SNPs that had more than 5% missing genotypes were then removed from the GWAS dataset.
2. Non-autosomal SNPs and four SNPs with duplicate positions were removed from the ADPC dataset. SNPs that failed a test for Hardy-Weinberg equilibrium (p-value < 0.0001), monomorphic SNPs and SNPs that had more than 5% missing genotypes were removed.
3. AT/CG SNPs were handled as follows, in both the GWAS and ADPC datasets: SNPs not in the reference panel were deleted; SNPs with MAF > 0.4 in either the dataset or reference panel were deleted; SNPs that differ in MAF more than 1% compared to the reference panel were deleted; for the remaining SNPs, if the minor allele was different from the reference panel minor allele, the strand of the SNP was flipped. These thresholds are stringent and a large number of SNPs were deleted (roughly 30 000 SNPs on the ADPC); however this strategy was preferred as incorrect AT/CG SNPs could potentially have a severe impact on imputation quality.
4. Next, the GWAS and ADPC datasets were uploaded to the imputation server for pre-imputation QC (Quality Control Only option). Based on the QC report downloaded from the server, SNPs typed on alternate strands compared to the reference panel were identified, and the alleles of these SNPs were flipped.
5. SNPs with common positions in the GWAS and ADPC datasets were extracted in order to identify discordant samples, and to verify the genotype concordance between the datasets. The proportion of alleles shared identical by-descent (IBD) between all pairs of samples were estimated using PLINK (PI_HAT output column), and GWAS and ADPC samples with identical sample identifiers but sharing less than 0.9 alleles IBD were removed from the GWAS and ADPC datasets (21 samples). The concordance between the remaining GWAS and ADPC samples was then checked (concordance was > 99.7% in all datasets).
6. Next, genotypes for corresponding GWAS and ADPC samples were merged into a single file. Discordant SNPs were handled as follows (same SNP in the ADPC and GWAS datasets, for the same sample, with different genotype calls): if a SNP was discordant in >= 5% of samples, the SNP was deleted from the dataset, else the SNP value was set to missing for the sample(s) it was discordant for.
7. Merged genotype files were uploaded to the imputation server. Imputed datasets and reports were downloaded after the job runs completed.
8. Unrelated phase 3 1000 Genomes Project (TGP) subjects of European ancestry (CEU, n=84) and African ancestry (YRI, n=84), as well as unrelated Native American (NAT) subjects from Mao et al. [40] (n=43), were merged with a MAF <1% and LD pruned data set of overlapping genotyped SNPs in all the GWAS+ADPC data sets. Principal components were then formed from the genotypes of the CAAPA and reference subjects by the R Bioconductor package. Based on this analysis, three outlier individuals that clustered close to the TGP CEU (European) subjects were identified, and these subjects were removed from the data sets prior to association testing.

Supplementary Note 7: Imputation pipeline

Creating the reference panel

1. Subject removal: Identity-by-descent (IBD) tests were performed on 917 CAAPA samples to test for unexpected relatedness. IBD tests were generated from the multi-sample VCF by KNOME, Inc. Subjects were removed in order to ensure that no duplicate, parent-offspring or sibling relationships are present in the data.
2. Variant filtering: Variants with genotyping quality scores (GQ) less than 20, depth (DP) less than 7, and in regions of segmental duplication were removed. Only bi-allelic variants with call rates of at least 95% were retained.
3. Phasing: Phasing was performed using ShapeIT (v2.r790) using 0.5 Mb windows.

As part of collaboration with the University of Michigan, this phased reference panel was uploaded to the Michigan imputation server.

Imputation

Full details of the pipeline are documented at https://imputationserver.sph.umich.edu/index.html#!pages/pipeline. Briefly, the steps in the pipeline is as follows:

1. Create chunks of size 20 Mb.
2. Do quality checks on chunks. For chunks, the amount of valid variants, the amount of variants found in the reference panel, and the sample call rate are checked. For variants, valid alleles (A/C/G/T), SNP call rate, allele frequency differences between study and reference panel, and allele switches and strand flips are checked.
3. Phase genotypes. 3 phasing algorithms are available: ShapeIt, HapiUR and Eagle. For this study, we used ShapeIt to phase genotypes.
4. Impute genotypes per chunk using Minimac3 and a window size of 500 Kb.
5. Merge chunks into a single file, per chromosome.

Supplementary Note 8: Quantifying imputation accuracy in the GWAS and ADPC data sets

To gauge imputation accuracy, 15% of the overlapping SNPs genotyped in the GRAAD(2), HUFS and GALA II datasets on commercial arrays (i.e. not on the ADPC) were selected at random and omitted from the combined datasets (10,751 SNPs in total that are also present in the CAAPA reference panel), and the imputations were run again. In terms of sample size, the first two datasets are the smallest and largest African American sample sizes respectively, and the GRAAD(2) dataset had the highest density of genotyped SNPs. GALA II participants are from Puerto Rico, and this dataset was examined in detail due to differences in the population structure of Puerto Rico compared to the other studies (Figure 1 and Supplementary Figure S1). Imputation error can be quantified by comparing typed SNPs and most probable imputed genotype at these masked SNPs. This comparison was done per SNP, yielding a concordance proportion per SNP (number of imputed genotypes that matched the typed genotypes exactly, divided by the number of total genotypes that were compared). The median and interquartile range concordance (i.e. the proportion of matching imputed and typed genotypes for a particular SNP) for GRAAD(2), GALA II and HUFS were 0.9886 [0.9545 – 1.0000], 0.9618 [0.9236 – 0.9829] and 0.9610 [0.9194 – 0.9838], respectively, indicating high imputation accuracy.

Supplementary Note 9: MEGA genotyping, SNP calling and quality control

6,371 CAAPA samples and 210 HapMap controls (additionally 12 samples were resequenced and 1 sample was sequenced in triplicate) were genotyped at Illumina on the Infinium Multi-Ethnic Global Array (MEGA) .

Preliminary SNP QC was performed using GenomeStudio. 319,491 SNPs that failed one or more of the following criteria were removed, as recommended by Illumina:

1. Call Frequency < 99% (we made this more stringent than Illumina's Call Frequency of 97%)
2. Cluster separation <= 0.3
3. Mean normalized intensity of first homozygote cluster (AA_R_Mean) <= 0.2 and/or mean normalized intensity of heterozygote cluster (AB_R_Mean) <= 0.2 and/or mean normalized intensity of second homozygote cluster (BB_R_Mean) <=0.2
4. Mean of normalized theta value for first homozygote cluster (AA_T_Mean) > 0.3
5. Standard deviation of normalized theta value for first homozygote cluster (AA_D_Mean) > 0.06
6. Mean of normalized theta value for second homozygote cluster (BB_T_Mean) < 0.7.
7. Standard deviation of normalized theta value for second homozygote cluster (BB_D_mean) > 0.06

Upon removal of the 319,491 SNPs (see above), 257 CAAPA samples were immediately discarded due to low genotype call rates of < 98.5%. All gender mismatches or ambiguities (remove females that has an F-statistic (calculated by PLINK v1.9) > 0.65 and remove all males that have have an F-statistic (calculated by PLINK v1.9) < 0.80) were also removed (n=509) from the remaining CAAPA samples.

For the remaining 5,605 CAAPA samples, sample quality control was performed independently for each sample cohort using an automated pipeline, as follows:

1. Remove samples with excess heterozygosity of > 3 standard deviations from mean of heterozygosity score calculated as: 1-[observed(HOM)/total] (n=77; performed on LD-pruned SNPs filtered for SNPs that have Hardy-Weinberg equilibrium (HWE) p-values > 1e-6 and MAF > 0.01)
2. Remove IBD duplicate samples with PLINK PI_HAT > 0.9 (n=166; performed on LD-pruned SNPs filtered for SNPs that have Hardy-Weinberg equilibrium (HWE) p-values > 1e-6 and MAF > 0.01)
3. Calculate kinship coefficient matrices using KING software
4. Remove PCA outliers > 6 standard deviations from mean based upon unrelated samples from kinship coefficient matrix to cluster PCA (n=13; performed on LD-pruned SNPs filtered for SNPs that have Hardy-Weinberg equilibrium (HWE) p-values > 1e-6 and MAF > 0.01)

One individual was also removed from BIAS due to high instances of Mendel errors (estimated using PLINK).

5,349 CAAPA samples passed all sample and SNP QC. This includes 743 samples from Peru, which were not used in the CAAPA meta-analysis, as these samples had low African ancestry.

Supplementary Note 10: Imputation of MEGA data sets.

Full details of the pipeline is documented at https://imputationserver.sph.umich.edu/index.html#!pages/pipeline. Imputation QC and imputation was performed within each sample cohort independently (BASS, BIAS, PGCA, HONDAS, JAAS, ProAR). All imputation was performed against the CAAPA-African American Panel reference genome provided by the Michigan Imputation Server. Briefly, the steps in the pipeline is as follows:

1. Split genotypes by each individual autosomal chromosome.
2. Perform quality checks on chromosomes. For each chromosome, the amount of valid variants, the amount of variants found in the reference panel, and the sample call rate are checked. For variants, valid alleles (A/C/G/T), SNP call rate, allele frequency differences between study and reference panel, and allele switches and strand flips are checked.
3. Phase genotypes. 3 phasing algorithms are available: ShapeIt, HapiUR and Eagle. For this study, we used ShapeIt to phase genotypes.
4. Impute genotypes per chromosome using Minimac3 and a window size of 500 Kb.

Supplementary Note 11: TaqMan genotyping and imputation data concordance

Genotyping was performed on the ABI 7900HT Sequence Detection System on 3 single nucleotide polymorphisms (SNPs). Using samples from BAGS and JAAS, the associations of low frequency SNPs were genotyped in order to check for concordance with imputed genotypes (rs114647118, rs73952947, rs73595000).

SNP Genotyping
1. Genomic DNA was extracted from peripheral blood samples.
2. Genotyping was performed using the TaqMan™ probe based, 5' nuclease assay with minor groove binder (MGB) chemistry (ABI, Foster City,CA). Genotyping was performed at a total volume of 5 µL per well, in 384-well MicroAmp Optical plates, using real-time detection followed by an endpoint read (Allelic discrimination) using the ABI 7900 HT Sequence Detection System using software SDS2.4 (Applied Biosystems, Foster City, CA, USA).
3. Fluorescence data files from each plate were analyzed on scatter plots in the allelic discrimination window by automated allele calling software SDS 2.4 (ABI, Foster City, CA) and visually inspected.
3. Quality control: 5 - 10% of the randomly selected samples were repeated as routine quality control procedure.

Genotype concordance
TaqMan genotype call quality was assessed by means of manual inspection of the allelic discrimination plots, checking the proportion of successful genotype calls, and tests for HWE. The imputed genotype calls for corresponding samples were then converted to "hard" genotype calls. The percentage of matching TaqMan and imputed genotype calls was then calculated. These results are summarized in Supplementary Table S10.

Supplementary Note 12: Local ancestry inference

Local ancestry of CAAPA study subjects were inferred using RFMix [41]. Input files for RMFix was created as follows: First, GWAS array and ADPC genotyped autosomal SNPs that overlapped in 7,416 African American and Barbados subjects were merged in order to perform haplotype phasing using the ShapeIT software package (361,411 SNPs). After performing phasing, the 7,416 genomes were merged with African and European TGP phase 3 genomes (99 CEU and 108 YRI subjects, files downloaded from ftp.1000genomes.ebi.ac.uk/vol1/ftp/release/20130502/ to data/raw/tgp_release_20130502). The genetic map required as input for local ancestry inference was downloaded from https://github.com/joepickrell/1000-genomes-genetic-maps/tree/master/interpolated_from_hapmap. Custom scripts were used to create input files for local ancestry inference according to the RFMix input file specification. The final input files for RFMix comprised 273,738 SNPs.

The same pipeline was used to infer local ancestry of the CAAPA BASS and JAAS studies, genotyped on the MEGA, and an additional 393 BAGS subjects, genotyped on the Omni 2.5 array but not genotyped on the ADPC. 964,332, 987,203 and 2,164,369 SNPs were phased together, for BASS, JAAS and BAGS, respectively. The final input files for RFMix comprised 849,958, 862,586 and 1,823,321 SNPs respectively.

Local ancestry calling of BioMe subjects was performed as follows: Phasing was performed per chromosome with the EAGLEv2.0.5 software using the genetic map (hg19) that is included in the EAGLEv2.0.5 package (url: https://data.broadinstitute.org/alkesgroup/Eagle/downloads/Eagle_v2.0.tar.gz). Three-way local ancestry calls were then generated for self-reported African American (AA, n=6568) and Hispanic/Latino individuals (H/L, n=9578) using the RFMix software over all 377,799 SNPs and using the default parameters. Reference panels were constructed to represent the three main continental-level ancestral populations likely to be present in BioMe AA and H/L populations, namely: European, African and Native American ancestry. We selected n=50 CEU and n=50 IBS samples from the Thousand Genomes Project representing European, n=50 YRI and n=50 LWK samples to represent African and, for representation of Native American haplotypes, indigenous Honduran and Columbian samples (n=25), Andean samples from Peru (n=25), Mayan samples from HGDP (n=25), and samples from Oaxaca, Mexico (n=25). When constructing the reference panels we selected

unrelated samples within each population that exhibited the least amount of admixture (quantified via running the ADMIXTURE software unsupervised at k=3 putative ancestral populations).

CAAPA studies genotyped on the ADPC (admixture mapping discovery)

RFMix inferred 15,824 local ancestry segments across the CAAPA genomes. The mean proportion of African ancestry inferred for each of these segments is summarized graphically in Supplementary Figure S21. Deviations between mean local African ancestry and mean genome-wide African ancestry are normally distributed, as expected (Supplementary Figure S22). Estimates of genome-wide ancestry from ADMIXTURE and RFMix were also highly concordant (Supplementary Figure S23), with a mean estimated difference of only 0.0009 (less than 0.1%).

CAAPA studies genotyped on the MEGA (admixture mapping replication)

RFMix inferred 15,999, 16,006 and 16,253 local ancestry segments across the BASS, JAAS and BAGS genomes, respectively. Deviations between mean local African ancestry and mean genome-wide African ancestry were normally distributed. Estimates of genome-wide ancestry from ADMIXTURE and RFMix were also highly concordant with a mean estimated difference of only -0.0078, -0.0132, -0.027 for BASS, JAAS and BAGS, respectively.

Supplementary Note 13: Analysis of chromosome 8p23 asthma associations

To fine-map the novel asthma association on chromosome 8p23 in the CAAPA meta-analysis, a set of credible SNPs were constructed using the Bayes Factor reported by the MR-MEGA software. SNPs +- 10KB from the lead SNP at hg19 position 2,550,802 were included in this analysis. The posterior probability of association for each SNP is the ratio of its Bayes Factor over the sum of the Bayes Factors of all the SNPs included in the analysis. SNPs were then ranked according to their Bayes Factors, and SNPs were selected for inclusion in the 99% credible set until their cumulative posterior probability attained or exceeded 0.99 (see Supplementary Table S22).

The following databases were searched in order to determine if any of the SNPs in the chromosome 8p23 region associated with asthma (SNPs in the credible set as well as SNPs with p < 10-5) are expression quantitative trait loci for one or more genes in relevant cell and tissue types: 1) whole blood, lung and EBV_transformed lymphocytes tissues in the GTEx eQTL Browser [42], 2) the gene expression data of 777 lymphoblastoid cell lines (LCLs) from the MuTHER database [43], 3) the transcription profiles of 405 and 550 lymphoblastoid cell lines from UK asthma (MRCA) and eczema (MRCE) family members [44], 4) eQTL data from monocytes of 1,490, subjects included in the GHS-express database [45], 5) transcriptional profiles from lung tissues of 1,111 subjects [46]. Only one SNP, rs13439436 (the 10th SNP in the credible set), was found to be an eQTL, for an uncharacterized gene in the GHS-express database (Illumina probe ID ILMN_1896420, Illumina gene ID HS.545993). ENCODE (Encyclopedia of DNA elements) chromHMM predictions also did not reveal any relevant transcriptional elements (the SNPs were located in low signal heterochromatin regions in the Gm12878, H1hesc, Hepg2, Hmec, Hsmm, Huvec, Nhek, Nhlf cell lines, and located in a weak transcribed region in the K562 cell line; annotations provided by the ANNOVAR software package). However, as these databases do not fully represent the spectrum of cell and tissue types relevant to asthma, and only minimally represent subjects of African ancestry, the lack of identification of eQTLs does not necessarily imply that the SNPs in this region are not transcriptionally active.

Because the SNPs in the credible set are intronic to a long non-coding RNA gene (LOC101927815) with no characterized function, the Hi-C Unifying Genomic Interrogator (HUGIn) online tool was used to visualize possible long-range chromatin interactions in three relevant tissue types (lymphoblastoid cells, fetal lung fibroblast cells, lung tissue) [47]. HUGIn displays data from multiple sources, including chromatin organization features (including long-range chromatin interactions from the analysis of Hi-C data) and gene expression data

(from tissue or cell line specific RNA-Seq data). In this way, should the association be due to the regulation of genes not in the immediate vicinity of the association signal, candidate genes explaining the association signal can be identified. These results are shown in Supplementary Figure S19. Two genes downstream from the lead SNP at position 2,550,801 are expressed in the investigated tissues and are possible asthma candidate genes: ARHGEF10 has been associated with exacerbations in chronic obstructive pulmonary disease [48], and this gene may also be a target of the well-known type 2 inflammation cytokine interleukin 33 [49], while genetic variants in MYOM2 are predictive of lung function in an isolated European ancestry (Hutterite) population [50]. The block surrounding the lead SNP at position 2,550,801 are possibly interacting with a downstream block at ~ 2.2 MB (FDR $p<0.05$ in all three tissue types and Bonferroni $p<0.05$ in fetal lung fibroblast cells) as well as a block at ~1.95 MB (FDR $p<0.05$ in lung). Interestingly, the latter block overlaps with the transcription start site of the MYOM2 gene.

Supplementary References

1. Mathias, R.A., et al., *A genome-wide association study on African-ancestry populations for asthma.* J Allergy Clin Immunol, 2010. **125**(2): p. 336-346 e4.
2. Torgerson, D.G., et al., *Genome-wide ancestry association testing identifies a common European variant on 6q14.1 as a risk factor for asthma in African American subjects.* J Allergy Clin Immunol, 2012. **130**(3): p. 622-629 e9.
3. Torgerson, D.G., et al., *Meta-analysis of genome-wide association studies of asthma in ethnically diverse North American populations.* Nat Genet, 2011. **43**(9): p. 887-92.
4. Padhukasahasram, B., et al., *Gene-based association identifies SPATA13-AS1 as a pharmacogenomic predictor of inhaled short-acting beta-agonist response in multiple population groups.* Pharmacogenomics J, 2014. **14**(4): p. 365-71.
5. Gould, W., et al., *Factors predicting inhaled corticosteroid responsiveness in African American patients with asthma.* J Allergy Clin Immunol, 2010. **126**(6): p. 1131-8.
6. Rumpel, J.A., et al., *Genetic ancestry and its association with asthma exacerbations among African American subjects with asthma.* J Allergy Clin Immunol, 2012. **130**(6): p. 1302-6.
7. Taylor, H.A., Jr., et al., *Toward resolution of cardiovascular health disparities in African Americans: design and methods of the Jackson Heart Study.* Ethn Dis, 2005. **15**(4 Suppl 6): p. S6-4-17.
8. Flores, C., et al., *African ancestry is associated with asthma risk in African Americans.* PLoS One, 2012. **7**(1): p. e26807.
9. Adeyemo, A., et al., *A genome-wide association study of hypertension and blood pressure in African Americans.* PLoS Genet, 2009. **5**(7): p. e1000564.
10. Faruque, M.U., et al., *Transferability of genome-wide associated loci for asthma in African Americans.* J Asthma, 2017. **54**(1): p. 1-8.
11. Moore, W.C., et al., *Identification of asthma phenotypes using cluster analysis in the Severe Asthma Research Program.* Am J Respir Crit Care Med, 2010. **181**(4): p. 315-23.
12. Barnes, K.C., et al., *Linkage of asthma and total serum IgE concentration to markers on chromosome 12q: evidence from Afro-Caribbean and Caucasian populations.* Genomics, 1996. **37**(1): p. 41-50.
13. Zambelli-Weiner, A., et al., *Evaluation of the CD14/-260 polymorphism and house dust endotoxin exposure in the Barbados Asthma Genetics Study.* J Allergy Clin Immunol, 2005. **115**(6): p. 1203-9.
14. Oliveira, R.R., et al., *Factors associated with resistance to Schistosoma mansoni infection in an endemic area of Bahia, Brazil.* Am J Trop Med Hyg, 2012. **86**(2): p. 296-305.
15. Grant, A.V., et al., *High heritability but uncertain mode of inheritance for total serum IgE level and Schistosoma mansoni infection intensity in a schistosomiasis-endemic Brazilian population.* J Infect Dis, 2008. **198**(8): p. 1227-36.
16. Vergara, C., et al., *African ancestry is a risk factor for asthma and high total IgE levels in African admixed populations.* Genet Epidemiol, 2013. **37**(4): p. 393-401.
17. Mallol, J., et al., *Prevalence of asthma symptoms in Latin America: the International Study of Asthma and Allergies in Childhood (ISAAC).* Pediatr Pulmonol, 2000. **30**(6): p. 439-44.

18. Sole, D., et al., *International Study of Asthma and Allergies in Childhood (ISAAC) written questionnaire: validation of the asthma component among Brazilian children.* J Investig Allergol Clin Immunol, 1998. **8**(6): p. 376-82.
19. Vergara, C., et al., *Gene encoding Duffy antigen/receptor for chemokines is associated with asthma and IgE in three populations.* Am J Respir Crit Care Med, 2008. **178**(10): p. 1017-22.
20. Oliveira, R.R., et al., *Schistosoma mansoni infection alters co-stimulatory molecule expression and cell activation in asthma.* Microbes Infect, 2009. **11**(2): p. 223-9.
21. Vergara, C., et al., *Association of G-protein-coupled receptor 154 with asthma and total IgE in a population of the Caribbean coast of Colombia.* Clin Exp Allergy, 2009. **39**(10): p. 1558-68.
22. Vergara, C. and L. Caraballo, *Asthma mortality in Columbia.* Ann Allergy Asthma Immunol, 1998. **80**(1): p. 55-60.
23. Bateman, E.D., et al., *Global strategy for asthma management and prevention: GINA executive summary.* Eur Respir J, 2008. **31**(1): p. 143-78.
24. Dennis, R., et al., *Asthma and other allergic conditions in Colombia: a study in 6 cities.* Ann Allergy Asthma Immunol, 2004. **93**(6): p. 568-74.
25. Dennis, R.J., et al., *Prevalence of asthma and other allergic conditions in Colombia 2009-2010: a cross-sectional study.* BMC Pulm Med, 2012. **12**: p. 17.
26. Vergara, C., et al., *African ancestry is associated with risk of asthma and high total serum IgE in a population from the Caribbean Coast of Colombia.* Hum Genet, 2009. **125**(5-6): p. 565-79.
27. Herrera-Paz, E.F., et al., *Allele frequencies distributions for 13 autosomal STR loci in 3 Black Carib (Garifuna) populations of the Honduran Caribbean coasts.* Forensic Sci Int Genet, 2008. **3**(1): p. e5-10.
28. Herrera-Paz, E.F., M. Matamoros, and A. Carracedo, *The Garifuna (Black Carib) people of the Atlantic coasts of Honduras: Population dynamics, structure, and phylogenetic relations inferred from genetic data, migration matrices, and isonymy.* Am J Hum Biol, 2010. **22**(1): p. 36-44.
29. Knight-Madden, J., et al., *Skin test reactivity to aeroallergens in Jamaicans: relationship to asthma.* West Indian Med J, 2006. **55**(3): p. 142-7.
30. Kumar, R., et al., *Factors associated with degree of atopy in Latino children in a nationwide pediatric sample: the Genes-environments and Admixture in Latino Asthmatics (GALA II) study.* J Allergy Clin Immunol, 2013. **132**(4): p. 896-905 e1.
31. Friedman, G.D., et al., *CARDIA: study design, recruitment, and some characteristics of the examined subjects.* J Clin Epidemiol, 1988. **41**(11): p. 1105-16.
32. Lettre, G., et al., *Genome-wide association study of coronary heart disease and its risk factors in 8,090 African Americans: the NHLBI CARe Project.* PLoS Genet, 2011. **7**(2): p. e1001300.
33. Bild, D.E., et al., *Multi-Ethnic Study of Atherosclerosis: objectives and design.* Am J Epidemiol, 2002. **156**(9): p. 871-81.
34. *The Atherosclerosis Risk in Communities (ARIC) Study: design and objectives. The ARIC investigators.* Am J Epidemiol, 1989. **129**(4): p. 687-702.
35. Pacheco, J.A., et al., *A highly specific algorithm for identifying asthma cases and controls for genome-wide association studies.* AMIA Annu Symp Proc, 2009. **2009**: p. 497-501.
36. Mathias, R.A., et al., *A continuum of admixture in the Western Hemisphere revealed by the African Diaspora genome.* Nat Commun, 2016. **7**: p. 12522.
37. Li, X., et al., *Genome-wide association studies of asthma indicate opposite immunopathogenesis direction from autoimmune diseases.* J Allergy Clin Immunol, 2012. **130**(4): p. 861-8 e7.
38. White, M.J., et al., *Novel genetic risk factors for asthma in African American children: Precision Medicine and the SAGE II Study.* Immunogenetics, 2016. **68**(6-7): p. 391-400.
39. Chen, G., et al., *Development of admixture mapping panels for African Americans from commercial high-density SNP arrays.* BMC Genomics, 2010. **11**: p. 417.
40. Mao, X., et al., *A genomewide admixture mapping panel for Hispanic/Latino populations.* Am J Hum Genet, 2007. **80**(6): p. 1171-8.

41. Maples, B.K., et al., *RFMix: a discriminative modeling approach for rapid and robust local-ancestry inference.* Am J Hum Genet, 2013. **93**(2): p. 278-88.
42. Consortium, G.T., *Human genomics. The Genotype-Tissue Expression (GTEx) pilot analysis: multitissue gene regulation in humans.* Science, 2015. **348**(6235): p. 648-60.
43. Grundberg, E., et al., *Mapping cis- and trans-regulatory effects across multiple tissues in twins.* Nat Genet, 2012. **44**(10): p. 1084-9.
44. Liang, L., et al., *A cross-platform analysis of 14,177 expression quantitative trait loci derived from lymphoblastoid cell lines.* Genome Res, 2013. **23**(4): p. 716-26.
45. Zeller, T., et al., *Genetics and beyond--the transcriptome of human monocytes and disease susceptibility.* PLoS One, 2010. **5**(5): p. e10693.
46. Hao, K., et al., *Lung eQTLs to help reveal the molecular underpinnings of asthma.* PLoS Genet, 2012. **8**(11): p. e1003029.
47. Martin, J.S., et al., *HUGIn: Hi-C Unifying Genomic Interrogator.* Bioinformatics, 2017. **33**(23): p. 3793-3795.
48. Singh, D., et al., *Altered gene expression in blood and sputum in COPD frequent exacerbators in the ECLIPSE cohort.* PLoS One, 2014. **9**(9): p. e107381.
49. Pinto, S.M., et al., *Quantitative phosphoproteomic analysis of IL-33-mediated signaling.* Proteomics, 2015. **15**(2-3): p. 532-44.
50. Yao, T.C., et al., *Genome-wide association study of lung function phenotypes in a founder population.* J Allergy Clin Immunol, 2014. **133**(1): p. 248-55 e1-10.
