# Supplementary material for: Association study in African-admixed populations across the Americas recapitulates asthma risk loci in non-African populations
Source: Nat Commun. 2019 Feb 20;10:880. doi: 10.1038/s41467-019-08469-7 (PMC6382865; doi:10.1038/s41467-019-08469-7)
Supplement: Supplementary file 3 — Description of Additional Supplementary Files [file 41467_2019_8469_MOESM3_ESM.pdf]

Supplementary Data 1:

Association and imputation quality summaries by individual study for selected SNPs

Supplementary Data 2:

Sensitivity analysis of 22 putatively causal SNPs on chr17q12-21.
